# Supplementary material for: Efficacy and safety of moderate-intensity rosuvastatin plus ezetimibe versus high-intensity rosuvastatin monotherapy in the treatment of composite cardiovascular events with hypercholesterolemia: A meta-analysis
Source: PLoS One. 2024 Nov 13;19(11):e0310696. doi: 10.1371/journal.pone.0310696 (PMC11559983; doi:10.1371/journal.pone.0310696)
Supplement: S1 Table — (DOC) [file pone.0310696.s001.doc]

**Supplementary tables**

**S1 Table. A meta-analysis of the general characteristics of the studies is included.**

**S2 Table. Test of heterogeneity Publication bias.**

**S3 Table. A meta-analysis of the of Outcome indicators the studies is included.**

**S1 Table. A meta-analysis of the general characteristics of the studies is included.**

| **Author year** | **Country** | **Trial Duration** | **Patient Comparison** | **R10+EZ10(n)** | **R20(n)** | **Age (years)** | | **Male (%)** | | **outcome indicator** | **Jadad**  **score** |
| --- | --- | --- | --- | --- | --- | --- | --- | --- | --- | --- | --- |
|  |  |  |  |  |  | **R10+EZ10** | **R20** | **R10+EZ10** | **R20** |  |  |
| **Bomlee2023** | Korea | 3-year | ASCVD | 1894 | 1886 | 63.98 ±10.12 | 64.58±10.24 | 1420  (75) | 1406  (74.5) | ①⑦⑧ | 6 |
| **Choi2023** | Korea | Week24 | ASCVD | 126 | 132 | 58.93±26.99 | 53.66±36.73 | 91  (72.2) | 104  (78.8) | ②⑧ | 6 |
| **Du2021** | China | 1-year | ASCVD | 35 | 35 | 56.54±6.49 | 57.03±6.46 | 20  ( 57.14) | 19  (54.29) | ② | 3 |
| **Feng2019** | China | Week24 | CAD | 35 | 34 | 59±9 | 61±8 | 28  ( 80) | 24  ( 71) | ②③④⑤⑧ | 3 |
| **Hong2018** | Korea | Week 8 | Hypercholesterolemia | 66 | 64 | 62.5 ±8.9 | 64.2±8.3 | 39  (59.1) | 40  (62.5) | ②⑧ | 4 |
| **Hyup lee2023** | Korea | 3-year | ASCVD | 273 | 301 | 77 ±2 | 77±2 | 173  (63.4) | 180 (59.8) | ①②⑧ | 6 |
|  |  |  |  | 1621 | 1585 | 61 ± 8 | 62 ±8 | 1247  (76.9) | 1226 (77.4) |  | 6 |
| **Joon lee2023** | Korea | 3-year | ASCVD+DM | 701 | 697 | 64±9 | 65±9 | 545  (77.7) | 515 (73.9) | ①②③④⑤⑦⑧ | 6 |
|  |  |  | ASCVD | 1193 | 1189 | 63±10 | 63±10 | 875  (73.3) | 891 (74.9) |  | 6 |
| **Kim2016** | Korea | Week 8 | Hypercholesterolemia | 203 | 204 | 64.2±7.9 | 64.3±9.3 | 113  (55.7) | 118  (57.8) | ②③④⑤⑥ | 6 |
| **Kim2018** | Korea | Week 8 | Hypercholesterolemia | 60 | 63 | 61.77±9.92 | 59.33±9.13 | 31  (51.7) | 39  (61.9) | ②③④⑤⑥⑧ | 6 |
| **Kim2022** | Korea | 3-year | ASCVD | 1894 | 1886 | 64±10 | 64±10 | 1420  (75) | 1406  (75) | ①②⑦⑧ | 5 |
| **Kim2023** | Korea | 3-year | ASCVD | 474 | 480 | 67.1±8.4 | 67.8±8.5 | Female | | ①②③④⑤⑦⑧ | 5 |
|  |  |  |  | 1420 | 1406 | 62.4±9.6 | 62.8±9.7 | Male | |  | 5 |
| **Lee2023** | Korea | 3-year | ASCVD | 757 | 754 | 63.6 ±9.9 | 64.3±10.3 | 616  (81.4) | 600 (79.6) | ①②⑦⑧ | 6 |
|  |  |  |  | 1137 | 1132 | 63.5 ±9.3 | 63.9 ±9.2 | 804  (70.7) | 806 (71.2) |  | 6 |
| **Li2020** | China | Week12 | LAA | 92 | 92 | 73.4±6.25 | 71.0±3.62 | 47  ( 51.09) | 49  (53.27) | ⑧ | 5 |
| **Ma2015** | China | Week16 | Hypercholesterolemia | 40 | 40 | 63. 5±9. 6 | 62. 8±9. 9 | NA | NA | ②③④⑤ | 3 |
| **Moon2023** | Korea | Week24 | ASCVD + DM | 48 | 51 | 61.88±6.47 | 61.16±7.09 | 28  (58.33) | 37 (72.55) | ②⑧ | 4 |
| **Ran2017** | China | Week 12 | ACS | 42 | 41 | 60.4 ±8.2 | 60.5±10.0 | 32  (76.2) | 30 (73.2) | ②③④⑤⑥⑧ | 5 |
| **Su2016** | China |  | CAD | 48 | 48 | 53.18±4.32 | 53.2±4.09 | 30  ( 62.5) | 31  (64.58) | ②③④⑤ | 4 |
| **Wang2018** | China | Week12 | Hypercholesterolemia+ DM | 26 | 26 | 57.3±10.4 | | NA | NA | ②③④⑤⑧ | 3 |
| **Xu2013** | China | Week16 | cerebral infarction | 29 | 27 | 60±11 | 60±0.9 | 16  ( 55.2) | 13  ( 48.1) | ②③④⑤ | 3 |
| **Yang2016** | Korea | Week 12 | CAD | 38 | 39 | 62.1 ±9.5 | 62.7 ±9.6 | 24  (63.2) | 26 (66.7) | ②③④⑤ | 5 |
| **Zhang2018** | China | 1-year | STEMI | 78 | 50 | 61.01±7. 57 | 60.9±8. 36 | 56  ( 71. 8) | 34  ( 68) | ⑧ | 3 |

Note:R: Rosuvastatin;EZ: ezetimibe;ASCVD:atherosclerotic cardiovascular disease;CAD:coronary artery disease;DM:diabetes mellitus；LAA:Large-artery atherosclerosis;ACS:acute coronary syndrome;STEMI:ST-segment elevation myocardial infarction.①long-term composite cardiovascular events②LDL-C③HDL-C④TC⑤TG⑥non-HDL-C⑦ the proportion of patients whose LDL-C levels were below 70 or 55 mg/dL ⑧the occurrence of adverse events

**S2 Table. Test of heterogeneity Publication bias.**

| **outcome** | **No. of study** | **95%CI** | **P value** | **I^2^(%)** |
| --- | --- | --- | --- | --- |
| **ACE** | Kim2023(2)[18] | [0.90, 0.97] | 0.0009 | 0 |
| **LDL-C** | Ma2015[21] | [-9.80, -7.31] | 0.0001 | 48 |
| **HDL-C** | Ma2015[21] | [-1.56, -0.49] | 0.0002 | 0 |
| **TC** | Ma2015[21] | [-9.83, -5.04] | 0.0001 | 61 |
| **TG** | Kim2023(2)[18] | [-6.84, -2.21] | 0.0001 | 38 |
| **N-HDL-C** | Ran2017[23] | [-12.25, -4.93] | 0.0001 | 0 |

**Table3. A meta-analysis of the of Outcome indicators the studies is included.**

| **First author,**  **reference** | **primary endpoint** | | **Secondary efficacy** | | **Individual clinical** | | **LDL-C** | | **HDL-C** | | **TC** | | **N-HDL-C** | | **TG** | | **LDL-C<70**  **mgdL** | | **LDL-C<55**  **mgdL** | | **overall adverse events** | |
| --- | --- | --- | --- | --- | --- | --- | --- | --- | --- | --- | --- | --- | --- | --- | --- | --- | --- | --- | --- | --- | --- | --- |
|  | **R10+EZ** | **R**  **20** | **R10+EZ** | **R**  **20** | **R10+EZ** | **R**  **20** | **R10+EZ** | **R20** | **R10+EZ** | **R20** | **R10+EZ** | **R20** | **R10+EZ** | **R20** | **R10+EZ** | **R20** | **R**  **10+EZ** | **R**  **20** | **R10+EZ** | **R**  **20** | **R10+EZ** | **R20** |
| **Bomlee2023** | 172 | 186 | 186 | 197 | 465 | 483 | - | - | - | - | - | - | - | - | - | - | 978 | 759 | - | - | 436 | 522 |
| **Choi2023** | - | - | - | - | - | - | -23.8±31.4 | -11.4±28.6 | - | - | - | - | - | - | - | - | - | - | - | - | 10 | 20 |
| **Du2021** | - | - | - | - | - | - | -59.12±5.65 | -49.4±27.14 | - | - | - | - | - | - | - | - | - | - | - | - | - | - |
| **Feng2019** | - | - | - | - | - | - | -55.8±16.39 | -47.96±17.77 | 3.6±9.4 | 4.6±9.01 | -28.6±30.48 | -24.4±32.48 | - | - | -15.1±59.83 | -10.9±64.95 | - | - | - | - | 6 | 9 |
| **Hong2018** | - | - | - | - | - | - | -57.1±18.5 | -49.2±27.1 | - | - | - | - | - | - | - | - | - | - | - | - | 10 | 16 |
| **Hyuplee2023**  **(1)** | 29 | 37 | 37 | 38 | - | - | -22.35±22.4 | -16.65±24.22 | - | - | - | - | - | - | - | - | - | - | - | - | 58 | 98 |
| **Hyuplee2023**  **(2)** | 143 | 149 | 149 | 159 | - | - | -23.05±23.43 | -13.7±23.57 | - | - | - | - | - | - | - | - | - | - | - | - | 412 | 510 |
| **Joonlee2023**  **(1)** | 103 | 109 | 111 | 113 | 406 | 414 | -50.9±20.42 | -57.08±22.35 | -1.35±11.59 | -0.35±11.51 | -24.7±29.59 | -15.13±1.36 | - | - | -12.35±55.94 | -3.3±59.36 | 581 | 441 | 302 | 164 | 433 | 530 |
| **Joonlee2023**  **(2)** | 69 | 77 | 75 | 84 | 301 | 319 | -95±23.64 | -77±30.41 | -1±10.29 | 0.7±10.61 | -24.4±31.67 | -12.95±29.23 | - | - | -16.8±62.02 | -3.89±64.41 | 397 | 318 | 261 | 166 | 132 | 198 |
| **Kim2016** | - | - | - | - | - | - | -57.6±17.4 | -56±17.2 | 14.1±28.5 | 11.7±29.99 | -39.6±19.95 | -32.9±20 | -54.9±24.22 | -45.8±24.28 | -22.7±49.87 | -13.4±49.99 | - | - | - | - | - | - |
| **Kim2018** | - | - | - | - | - | - | -30±29.14 | -21.7±30.71 | 12.47±25.6 | 13.9±20.25 | -38.92±13.47 | -32.45±14.88 | -52.85±15.64 | -45.04±17.3 | -20.45±32.53 | -19.36±32.64 | - | - | - | - | 12 | 15 |
| **Kim2022** | 172 | 186 | 186 | 197 | 713 | 788 | -25.3±27.43 | -18±28.31 | - | - |  |  | - | - | - | - | 978 | 759 | - | - | 594 | 752 |
| **Kim2023(1)** | 134 | 146 | 145 | 155 | 769 | 584 | -30.8±24.47 | -22.9±26.28 | -1±10.4 | 0.7±11.24 | -22.7±29.77 | -13.05±31.89 | - | - | -11.8±58.72 | -3.55±61.17 | 739 | 585 | 427 | 267 | 486 | 586 |
| **Kim2023(2)** | 38 | 40 | 41 | 42 | 153 | 150 | -47.7±7.13 | -36.36±7.77 | -0.3±12.86 | -0.05±12.57 | -27.75±30.99 | -15.05±30.68 | - | - | -18.4±56.33 | 0.46±55.87 | 239 | 173 | 136 | 63 | 138 | 196 |
| **Lee2023(1)** | 87 | 88 | - | - | - | - | -30.24±14.67 | -24.3±14.34 | - | - | - | - | - | - | - | - | 598 | 436 | - | - | 57 | 100 |
| **Lee2023(2)** | 85 | 88 | - | - | - | - | -29.88±13.58 | -13.14±13.56 | - | - | - | - | - | - | - | - | 380 | 323 | - | - | 34 | 56 |
| **Li2020** | - | - | - | - | - | - | - | - | - | - | - | - | - | - | - | - | - | - | - | - | 34 | 52 |
| **Ma2015** | - | - | - | - | - | - | -16.38±8.04 | -16.74±8.49 | 18.9±3.43 | 12.6±3.42 | -45±8.62 | 37.8±8.2 | - | - | -28.8±7.93 | -24.3±7.29 | - | - | - | - | - | - |
| **Moon2023** | - | - | - | - | - | - | -39.6±19.33 | -34.74±18.1 | - | - | - | - | - | - | - | - | - | - | - | - | 27 | 17 |
| **Ran2017** | - | - | - | - | - | - | -21.4±24.77 | -13.75±23.33 | 2±6 | 2±7 | -96±29.81 | -81±40.63 | -98±30 | -56±38.2 | -31±36.59 | -27±38.3 | - | - | - | - | 4 | 11 |
| **Su2016** | - | - | - | - | - | - | -23.7±24.98 | -15.4±25.98 | 1.8±5.51 | 1.62±5.67 | -56.34±20.68 | -52.38±19.04 | - | - | -8.28±10.19 | -7.2±9.39 | - | - | - | - | - | - |
| **Wang2018** | - | - | - | - | - | - | -22.7±24.91 | -14.7±25.73 | 0.9±4.08 | 0.72±4.42 | -53.82±21.85 | -46.62±21.18 | - | - | -21.06±8.49 | -20.34±7.78 | - | - | - | - | 2 | 5 |
| **Xu2013** | - | - | - | - | - | - | -18.8±32.2 | -9.7±34.5 | 1.44±4.26 | 0.54±6.92 | -18±11.26 | -18.36±10.44 | - | - | -2.88±7.15 | -0.9±8.4 | - | - | - | - | - | - |
| **Yang2016** | - | - | - | - | - | - | -23.5±29.4 | -12.6±31.9 | 5.1±17.7 | 5.9±18.4 | -39.2±12.8 | -39.2±13.2 | - | - | -24.6±30.43 | -25.9±35.4 | - | - | - | - | - | - |
| **Zhang2018** | - | - | - | - | - | - | - | - | - | - | - | - | - | - | - | - | - | - | - | - | 4 | 8 |

**Name of data extractors:Lingyan Liu**

**Date of data extraction：November 2023 to December 2023**
