# Supplementary material for: Efficacy and safety of moderate-intensity rosuvastatin plus ezetimibe versus high-intensity rosuvastatin monotherapy in the treatment of composite cardiovascular events with hypercholesterolemia: A meta-analysis
Source: PLoS One. 2024 Nov 13;19(11):e0310696. doi: 10.1371/journal.pone.0310696 (PMC11559983; doi:10.1371/journal.pone.0310696)
Supplement: S2 File — (DOC) [file pone.0310696.s003.doc]

**Supplementary tables**

**S1 Table. A numbered table of all studies identified in the literature search, with the full title of each article, including those that were excluded from the analyses.**

**S2 Table. Quality evaluation results of the literature.**

**Table1. A numbered table of all studies identified in the literature search, with the full title of each article, including those that were excluded from the analyses.**

| **number** | **Study** | **Status** |
| --- | --- | --- |
| **1** | Long-term efficacy and safety of moderate-intensity statin with ezetimibe combination therapy versus high-intensity statin monotherapy in patients with atherosclerotic cardiovascular disease (RACING): a randomised, open-label, non-inferiority trial. | remove |
| **2** | Treat-to-Target or High-Intensity Statin in Patients With Coronary Artery Disease: A Randomized Clinical Trial. | remove |
| **3** | Effect of Alirocumab Added to High-Intensity Statin Therapy on Coronary Atherosclerosis in Patients With Acute Myocardial Infarction: The PACMAN-AMI Randomized Clinical Trial. | remove |
| **4** | Combination Moderate-Intensity Statin and Ezetimibe Therapy for Elderly Patients With Atherosclerosis. | remove |
| **5** | Beta-Blockers after Myocardial Infarction and Preserved Ejection Fraction. | remove |
| **6** | Combination Moderate-Intensity Statin and Ezetimibe Therapy for Elderly Patients With Atherosclerosis. | remove |
| **7** | Alirocumab and Cardiovascular Outcomes after Acute Coronary Syndrome. | remove |
| **8** | Moderate-Intensity Statin With Ezetimibe Combination Therapy vs High-Intensity Statin Monotherapy in Patients at Very High Risk of Atherosclerotic Cardiovascular Disease: A Post Hoc Analysis From the RACING Randomized Clinical Trial. | remove |
| **9** | Comparative efficacy and safety among high-intensity statins. Systematic Review and Meta-Analysis. | remove |
| **10** | Obicetrapib plus ezetimibe as an adjunct to high-intensity statin therapy: A randomized phase 2 trial. | remove |
| **11** | The Efficacy and Safety of Moderate-Intensity Rosuvastatin with Ezetimibe versus High-Intensity Rosuvastatin in High Atherosclerotic Cardiovascular Disease Risk Patients with Type 2 Diabetes Mellitus: A Randomized, Multicenter, Open, Parallel, Phase 4 Study. | remove |
| **12** | Evolocumab for Early Reduction of LDL Cholesterol Levels in Patients With Acute Coronary Syndromes (EVOPACS). | remove |
| **13** | Comparative effectiveness of statins on non-high density lipoprotein cholesterol in people with diabetes and at risk of cardiovascular disease: systematic review and network meta-analysis. | remove |
| **14** | A Randomized, Multicenter, Double-blind, Placebo-Controlled Study to Evaluate the Efficacy and Safety of a Quadruple Combination of Amlodipine, Losartan, Rosuvastatin, and Ezetimibe in Patients with Concomitant Essential Hypertension and Dyslipidemia. | remove |
| **15** | A Phase III, Multicenter, Randomized, Double-blind, Active Comparator Clinical Trial to Compare the Efficacy and Safety of Combination Therapy With Ezetimibe and Rosuvastatin Versus Rosuvastatin Monotherapy in Patients With Hypercholesterolemia: I-ROSETTE (Ildong Rosuvastatin & Ezetimibe for Hypercholesterolemia) Randomized Controlled Trial. | remove |
| **16** | Ezetimibe combination therapy with statin for non-alcoholic fatty liver disease: an open-label randomized controlled trial (ESSENTIAL study). | remove |
| **17** | Effects of statin therapy on diagnoses of new-onset diabetes and worsening glycaemia in large-scale randomised blinded statin trials: an individual participant data meta-analysis. | remove |
| **18** | Ezetimibe combination therapy with statin for non-alcoholic fatty liver disease: an open-label randomized controlled trial (ESSENTIAL study). | remove |
| **19** | Effects of statin therapy on diagnoses of new-onset diabetes and worsening glycaemia in large-scale randomised blinded statin trials: an individual participant data meta-analysis. | remove |
| **20** | Coordinated Care to Optimize Cardiovascular Preventive Therapies in Type 2 Diabetes: A Randomized Clinical Trial. | remove |
| **21** | Effect of statin therapy on muscle symptoms: an individual participant data meta-analysis of large-scale, randomised, double-blind trials. | remove |
| **22** | Statins for Prevention of Cardiovascular Disease in Adults: Evidence Report and Systematic Review for the US Preventive Services Task Force. | remove |
| **23** | Effect of Alirocumab on Lipoprotein(a) and Cardiovascular Risk After Acute Coronary Syndrome. | remove |
| **24** | Effects of alirocumab on cardiovascular and metabolic outcomes after acute coronary syndrome in patients with or without diabetes: a prespecified analysis of the ODYSSEY OUTCOMES randomised controlled trial. | remove |
| **25** | Moderate-intensity statin plus ezetimibe vs high-intensity statin according to baseline LDL-C in the treatment of atherosclerotic cardiovascular disease: A post-hoc analysis of the RACING randomized trial. | remove |
| **26** | Antiphospholipid syndrome. | remove |
| **27** | Association of Bempedoic Acid Administration With Atherogenic Lipid Levels in Phase 3 Randomized Clinical Trials of Patients With Hypercholesterolemia. | remove |
| **28** | Effects of routine early treatment with PCSK9 inhibitors in patients undergoing primary percutaneous coronary intervention for ST-segment elevation myocardial infarction: a randomised, double-blind, sham-controlled trial. | remove |
| **29** | Efficacy and Safety of Single-Pill Combination of Rosuvastatin and Ezetimibe in Chinese Patients with Primary Hypercholesterolemia Inadequately Controlled by Statin Treatment (ROZEL): A Randomized, Double-Blind, Double Dummy, Active-Controlled Phase 3 Clinical Trial. | remove |
| **30** | High-Dose Versus Low-Dose Pitavastatin in Japanese Patients With Stable Coronary Artery Disease (REAL-CAD): A Randomized Superiority Trial. | remove |
| **31** | Effect of rosuvastatin 20 mg versus rosuvastatin 5 mg plus ezetimibe on statin side-effects in elderly patients with atherosclerotic cardiovascular disease: Rationale and design of a randomized, controlled SaveSAMS trial. | remove |
| **32** | Pragmatic evaluation of events and benefits of lipid lowering in older adults (PREVENTABLE): Trial design and rationale. | remove |
| **33** | Medium-intensity statin with ezetimibe versus high-intensity statin in acute ischemic cerebrovascular disease (MESIA): A randomized clinical trial. | remove |
| **34** | Effectiveness of low-intensity atorvastatin 5 mg and ezetimibe 10 mg combination therapy compared with moderate-intensity atorvastatin 10 mg monotherapy: A randomized, double-blinded, multi-center, phase III study. | remove |
| **35** | Impact of Bempedoic Acid on Total Cardiovascular Events: A Prespecified Analysis of the CLEAR Outcomes Randomized Clinical Trial. | remove |
| **36** | Colchicine and high-intensity rosuvastatin in the treatment of non-critically ill patients hospitalised with COVID-19: a randomised clinical trial. | remove |
| **37** | High-intensity statin therapy in patients with chronic kidney disease: a systematic review and meta-analysis. | remove |
| **38** | Pharmacogenomic Study of Statin-Associated Muscle Symptoms in the ODYSSEY OUTCOMES Trial. | remove |
| **39** | Statins and the cholesterol mortality paradox. | remove |
| **40** | Pharmacokinetic Interaction Among Ezetimibe, Rosuvastatin, and Telmisartan. | remove |
| **41** | A systematic review and meta-analysis of the effect of high-intensity statin on coronary microvascular dysfunction. | remove |
| **42** | Distinct effects of rosuvastatin and rosuvastatin/ezetimibe on senescence markers of CD8+ T cells in patients with type 2 diabetes mellitus: a randomized controlled trial. | remove |
| **43** | In ASCVD, moderate-intensity statin + ezetimibe was noninferior to high-intensity statin alone at 3 y. | remove |
| **44** | The Legacy Effect in Treating Hypercholesterolemia. | remove |
| **45** | Highlights of Cardiovascular Disease Prevention Studies Presented at the 2023 American College of Cardiology Conference. | remove |
| **46** | The Legacy Effect in Treating Hypercholesterolemia. | remove |
| **47** | Comparative Safety and Efficacy of Low/Moderate-Intensity Statin plus Ezetimibe Combination Therapy vs. High-Intensity Statin Monotherapy in Patients with Atherosclerotic Cardiovascular Disease: An Updated Meta-Analysis. | remove |
| **48** | Atherosclerotic Cardiovascular Disease in Women: Providing Protection With Lipid-altering Agents. | remove |
| **49** | Effects of Fixed-dose Combination of Low-intensity Rosuvastatin and Ezetimibe Versus Moderate-intensity Rosuvastatin Monotherapy on Lipid Profiles in Patients With Hypercholesterolemia: A Randomized, Double-blind, Multicenter, Phase III Study. | remove |
| **50** | Comparative efficacy of fixed-dose statin and antihypertensive agent combinations: A network meta-analysis of randomized controlled trials. | remove |
| **51** | The Effects of Statin Dose, Lipophilicity, and Combination of Statins plus Ezetimibe on Circulating Oxidized Low-Density Lipoprotein Levels: A Systematic Review and Meta-Analysis of Randomized Controlled Trials. | remove |
| **52** | A PRISMA-compliant systematic review and meta-analysis of randomized controlled trials investigating the effects of statin therapy on plasma lipid concentrations in HIV-infected patients. | remove |
| **53** | Effect of rosuvastatin versus atorvastatin on new-onset diabetes mellitus in patients treated with high-intensity statin therapy for coronary artery disease: a post-hoc analysis from the LODESTAR randomized clinical trial. | remove |
| **54** | A Phase 3 Randomized Controlled Trial to Evaluate Efficacy and Safety of New-Formulation Zenon (Rosuvastatin/Ezetimibe Fixed-Dose Combination) in Primary Hypercholesterolemia Inadequately Controlled by Statins. | remove |
| **55** | Lipid management in ACS: Should we go lower faster? | remove |
| **56** | Combination Therapy of Rosuvastatin and Ezetimibe in Patients with High Cardiovascular Risk. | remove |
| **57** | Treat-to-target or high-intensity statin treatment in older adults with coronary artery disease: a post hoc analysis of the LODESTAR trial. | remove |
| **58** | High-intensity statin therapy yields better outcomes in acute coronary syndrome patients: a meta-analysis involving 26,497 patients. | remove |
| **59** | Unmet Need for Adjunctive Dyslipidemia Therapy in Hypertriglyceridemia Management. | remove |
| **60** | A Comparison of Rosuvastatin Monotherapy and Rosuvastatin Plus Ezetimibe Combination Therapy in Patients With Type 2 Diabetes: A Meta-Analysis of Randomized Controlled Trials. | remove |
| **61** | Pharmacokinetic Interactions and Tolerability of Rosuvastatin and Ezetimibe: A Randomized, Phase 1, Crossover Study in Healthy Chinese Participants. | remove |
| **62** | Effectiveness of lipid-lowering therapy on mortality and major adverse cardiovascular event outcomes in patients undergoing percutaneous coronary intervention: a network meta-analysis of randomised controlled trials. | remove |
| **63** | One-Year Effects of High-Intensity Statin on Bioactive Lipids: Findings From the JUPITER Trial. | remove |
| **64** | The comparative impact among different intensive statins and combination therapies with niacin/ezetimibe on carotid intima-media thickness: a systematic review, traditional meta-analysis, and network meta-analysis of randomized controlled trials. | remove |
| **65** | Pharmacokinetic and pharmacodynamic interaction between ezetimibe and rosuvastatin in healthy male subjects. | remove |
| **66** | Pharmacokinetic Interaction Between Telmisartan and Rosuvastatin/Ezetimibe After Multiple Oral Administration in Healthy Subjects. | remove |
| **67** | Lipid lowering effects of the CETP inhibitor obicetrapib in combination with high-intensity statins: a randomized phase 2 trial. | remove |
| **68** | The effect of statin treatment on circulating coenzyme Q10 concentrations: an updated meta-analysis of randomized controlled trials. | remove |
| **69** | A randomized, controlled comparison of different intensive lipid-lowering therapies in Chinese patients with non-ST-elevation acute coronary syndrome (NSTE-ACS): Ezetimibe and rosuvastatin versus high-dose rosuvastatin. | remove |
| **70** | Meta-analysis of the effect of statins on renal function. | remove |
| **71** | Ezetimibe in high-risk, previously treated statin patients: a systematic review and network meta-analysis of lipid efficacy. | remove |
| **72** | Ezetimibe in high-risk, previously treated statin patients: a systematic review and network meta-analysis of lipid efficacy. | remove |
| **73** | Comparative Efficacy of Rosuvastatin Monotherapy and Rosuvastatin/Ezetimibe Combination Therapy on Insulin Sensitivity and Vascular Inflammatory Response in Patients with Type 2 Diabetes Mellitus. | remove |
| **74** | Efficacy and safety of adding alirocumab to rosuvastatin versus adding ezetimibe or doubling the rosuvastatin dose in high cardiovascular-risk patients: The ODYSSEY OPTIONS II randomized trial. | remove |
| **75** | Comparative Efficacy of Rosuvastatin Monotherapy and Rosuvastatin/Ezetimibe Combination Therapy on Insulin Sensitivity and Vascular Inflammatory Response in Patients with Type 2 Diabetes Mellitus. | remove |
| **76** | Effect of Alirocumab Added to High-Intensity Statin on Platelet Reactivity and Noncoding RNAs in Patients with AMI: A Substudy of the PACMAN-AMI Trial. | remove |
| **77** | Intensity of statin therapy and muscle symptoms: a network meta-analysis of 153 000 patients. | remove |
| **78** | Overall Mortality and LDL Cholesterol Reduction in Secondary Prevention Trials of Cardiovascular Disease. | remove |
| **79** | Meta-Analysis for Impact of Statin on Mortality After Transcatheter Aortic Valve Implantation. | remove |
| **80** | Statins and their increased risk of inducing diabetes. | remove |
| **81** | Effect of Statins on Serum level of hs-CRP and CRP in Patients with Cardiovascular Diseases: A Systematic Review and Meta-Analysis of Randomized Controlled Trials. | remove |
| **82** | Association Between Age and Low-Density Lipoprotein Cholesterol Response to Statins : A Danish Nationwide Cohort Study. | remove |
| **83** | Effects of Ezetimibe/Simvastatin and Rosuvastatin on Oxidative Stress in Diabetic Neuropathy: A Randomized, Double-Blind, Placebo-Controlled Clinical Trial. | remove |
| **84** | Two randomized controlled trials of nudges to encourage referrals to centralized pharmacy services for evidence-based statin initiation in high-risk patients: Rationale and design of the SUPER LIPID program. | remove |
| **85** | Appropriateness of Intensive Statin Treatment in People with Type Two Diabetes and Mild Hypercholesterolemia: A Randomized Clinical Trial. | remove |
| **86** | The impact of the time of drug administration on the effectiveness of combined treatment of hypercholesterolemia with Rosuvastatin and Ezetimibe (RosEze): study protocol for a randomized controlled trial. | remove |
| **87** | Safety and Effectiveness of High-Intensity Statins Versus Low/Moderate-Intensity Statins Plus Ezetimibe in Patients With Atherosclerotic Cardiovascular Disease for Reaching LDL-C Goals: A Systematic Review and Meta-Analysis. | remove |
| **88** | Safety and effectiveness of the association ezetimibe-statin (E-S) versus high dose rosuvastatin after acute coronary syndrome: the SAFE-ES study. | remove |
| **89** | Effect of different types and dosages of statins on plasma lipoprotein(a) levels: A network meta-analysis. | remove |
| **90** | Eicosapentaenoic and docosahexaenoic acid supplementation and coronary artery calcium progression in patients with coronary artery disease: A secondary analysis of a randomized trial. | remove |
| **91** | Antidyslipidemia Pharmacotherapy in Chronic Kidney Disease: A Systematic Review and Bayesian Network Meta-Analysis. | remove |
| **92** | Statins and coronary artery bypass graft surgery: preoperative and postoperative efficacy and safety. | remove |
| **93** | Factors Associated With Enhanced Low-Density Lipoprotein Cholesterol Lowering With Bempedoic Acid. | remove |
| **94** | Ezetimibe and Rosuvastatin Combination Treatment Can Reduce the Dose of Rosuvastatin Without Compromising Its Lipid-lowering Efficacy. | remove |
| **95** | Efficacy, safety and effect on biomarkers related to cholesterol and lipoprotein metabolism of rosuvastatin 10 or 20 mg plus ezetimibe 10 mg vs. simvastatin 40 or 80 mg plus ezetimibe 10 mg in high-risk patients: Results of the GRAVITY randomized study. | remove |
| **96** | Lipid lowering for prevention of venous thromboembolism: a network meta-analysis. | remove |
| **97** | Managing the underestimated risk of statin-associated myopathy. | remove |
| **98** | Intensive vs non-intensive statin pretreatment before percutaneous coronary intervention in Chinese patients: A meta-analysis of randomized controlled trials. | remove |
| **99** | Meta-analysis comparing the effects of rosuvastatin versus atorvastatin on regression of coronary atherosclerotic plaques. | remove |
| **100** | High-intensity statin monotherapy versus moderate-intensity statin plus ezetimibe therapy: effects on vascular biomarkers. | remove |
| **101** | Low-Density Lipoprotein Cholesterol After an Acute Coronary Syndrome: How Low to Go? | remove |
| **102** | Statins in the elderly: a patient-focused approach. | remove |
| **103** | Lowering Targeted Atherogenic Lipoprotein Cholesterol Goals for Patients at "Extreme" ASCVD Risk. | remove |
| **104** | Effects of high-intensity statin combined with telmisartan versus amlodipine on glucose metabolism in hypertensive atherosclerotic cardiovascular disease patients with impaired fasting glucose: A randomized multicenter trial. | remove |
| **105** | Managing hypertriglyceridemia for cardiovascular disease prevention: Lessons from the PROMINENT trial. | remove |
| **106** | Switching from statin monotherapy to ezetimibe/simvastatin or rosuvastatin modifies the relationships between apolipoprotein B, LDL cholesterol, and non-HDL cholesterol in patients at high risk of coronary disease. | remove |
| **107** | The impact of statin therapy on plasma levels of von Willebrand factor antigen. Systematic review and meta-analysis of randomised placebo-controlled trials. | remove |
| **108** | Pharmacokinetic interactions and tolerability of rosuvastatin and ezetimibe: an open-label, randomized, multiple-dose, crossover study in healthy male volunteers. | remove |
| **109** | Cholesteryl Ester Transfer Protein Inhibition Reduces Major Adverse Cardiovascular Events by Lowering Apolipoprotein B Levels. | remove |
| **110** | High-intensity Statin Treatments in Clinically Stable Patients on Aspirin Monotherapy 12 Months After Drug-eluting Stent Implantation: A Randomized Study. | remove |
| **111** | Ezetimibe Improves Rosuvastatin Effects on Inflammation and Vascular Endothelial Function in Acute Coronary Syndrome Patients Undergoing PCI. | remove |
| **112** | Efficacy and safety of alirocumab as add-on therapy in high-cardiovascular-risk patients with hypercholesterolemia not adequately controlled with atorvastatin (20 or 40 mg) or rosuvastatin (10 or 20 mg): design and rationale of the ODYSSEY OPTIONS Studies. | remove |
| **113** | Efficacy and Safety of Lipid-Lowering Drugs of Different Intensity on Clinical Outcomes: A Systematic Review and Network Meta-Analysis. | remove |
| **114** | Prevalence and initiation of statin therapy in the oldest old-a longitudinal population-based study. | remove |
| **115** | PCSK9 inhibitor valuation: A science-based review of the two recent models. | remove |
| **116** | Effect of Eicosapentaenoic Acid/Docosahexaenoic Acid on Coronary High-Intensity Plaques Detected Using Noncontrast T1-weighted Imaging: The AQUAMARINE EPA/DHA Randomized Study. | remove |
| **117** | Effects of rosuvastatin versus atorvastatin on small dense low-density lipoprotein: a meta-analysis of randomized trials. | remove |
| **118** | Time-Dependent Cardiovascular Treatment Benefit Model for Lipid-Lowering Therapies. | remove |
| **119** | Hyperlipidemia management in diabetes: First line or supportive therapy? | remove |
| **120** | Gateways to clinical trials. | remove |
| **121** | Guidelines versus trial-evidence for statin use in primary prevention: The Copenhagen General Population Study. | remove |
| **122** | Effect of statins on the plasma/serum levels of inflammatory markers in patients with cardiovascular disease; a systematic review and meta-analysis of randomized clinical trials. | remove |
| **123** | Efficacy and safety of rosuvastatin 40 mg alone or in combination with ezetimibe in patients at high risk of cardiovascular disease (results from the EXPLORER study). | remove |
| **124** | Systematic literature review and meta-analysis of dual therapy with fenofibrate or fenofibric acid and a statin versus a double or equivalent dose of statin monotherapy. | remove |
| **125** | Gateways to clinical trials. | remove |
| **126** | Factors Influencing Decision Making for Carotid Endarterectomy versus Stenting in the Very Elderly. | remove |
| **127** | Lipid-altering efficacy of ezetimibe/simvastatin 10/20 mg compared to rosuvastatin 10 mg in high-risk patients with and without type 2 diabetes mellitus inadequately controlled despite prior statin monotherapy. | remove |
| **128** | Effects of alirocumab on endothelial function and coronary atherosclerosis in myocardial infarction: A PACMAN-AMI randomized clinical trial substudy. | remove |
| **129** | Systematic Review and Network Meta-Analysis on the Efficacy of Evolocumab and Other Therapies for the Management of Lipid Levels in Hyperlipidemia. | remove |
| **130** | Prevalence and Effects of High-Intensity Statins for Japanese Patients Presenting With Acute Coronary Syndrome - A Post Hoc Secondary Analysis of STOPDAPT-2 ACS. | remove |
| **131** | Pharmacodynamic interaction between ezetimibe and rosuvastatin. | remove |
| **132** | Toward "pain-free" statin prescribing: clinical algorithm for diagnosis and management of myalgia. | remove |
| **133** | Low dose of ROSuvastatin in combination with EZEtimibe effectively and permanently reduce low density lipoprotein cholesterol concentration independently of timing of administration (ROSEZE): A randomized, crossover study - preliminary results. | remove |
| **134** | PCSK9 inhibitor recaticimab for hypercholesterolemia on stable statin dose: a randomized, double-blind, placebo-controlled phase 1b/2 study. | remove |
| **135** | Coronary Artery Calcium to Improve the Efficiency of Randomized Controlled Trials in Primary Cardiovascular Prevention. | remove |
| **136** | Comparison of High-Dose Rosuvastatin Versus Low-Dose Rosuvastatin Plus Ezetimibe on Carotid Atherosclerotic Plaque Inflammation in Patients with Acute Coronary Syndrome. | remove |
| **137** | Rationale and design of the Pemafibrate to Reduce Cardiovascular Outcomes by Reducing Triglycerides in Patients with Diabetes (PROMINENT) study. | remove |
| **138** | Pharmacokinetics and bioequivalence of a rosuvastatin/ezetimibe fixed-dose combination tablet versus single agents in healthy male subjects. | remove |
| **139** | Efficacy and Safety of Ezetimibe and Rosuvastatin Combination Therapy Versus Those of Rosuvastatin Monotherapy in Patients With Primary Hypercholesterolemia. | remove |
| **140** | Efficacy of high intensity atorvastatin versus moderate intensity atorvastatin for acute coronary syndrome patients with diabetes mellitus. | remove |
| **141** | Statins and Mortality of Patients After Transcatheter Aortic Valve Implantation: A Systematic Review and Meta-analysis. | remove |
| **142** | Gateways to clinical trials. | remove |
| **143** | The case for intensive statin therapy after acute coronary syndromes. | remove |
| **144** | High-intensity statin therapy alters the natural history of diabetic coronary atherosclerosis: insights from SATURN. | remove |
| **145** | Statin use and risk for type 2 diabetes: what clinicians should know. | remove |
| **146** | Utilization of and Adherence to Guideline-Recommended Lipid-Lowering Therapy After Acute Coronary Syndrome: Opportunities for Improvement. | remove |
| **147** | Disparate statin prescribing following hospital discharge for stroke or transient ischemic attack: Findings from COMPASS. | remove |
| **148** | Risk of mortality and recurrent cardiovascular events in patients with acute coronary syndromes on high intensity statin treatment. | remove |
| **149** | Effect of Intensive Lipid-Lowering Therapy on Coronary Plaque Stabilization Derived from Optical Coherence Tomography: a Meta-analysis and Meta-regression. | remove |
| **150** | Impact of lipid levels and high-intensity statins on vein graft patency after CABG: Midterm results of the ACTIVE trial. | remove |
| **151** | The influence of statin monotherapy and statin-ezetimibe combined therapy on FoxP3 and IL 10 mRNA expression in patients with coronary artery disease. | remove |
| **152** | Effects of Combination of Ezetimibe and Rosuvastatin on Coronary Artery Plaque in Patients with Coronary Heart Disease. | remove |
| **153** | Effects of rosuvastatin/ezetimibe on senescence of CD8+ T-cell in type 2 diabetic patients with hypercholesterolemia: A study protocol. | remove |
| **154** | Lipoprotein(a) and coronary atheroma progression rates during long-term high-intensity statin therapy: Insights from SATURN. | remove |
| **155** | Statin effect on coronary calcium distribution, mass and volume scores and associations with immune activation among HIV+ persons on antiretroviral therapy. | remove |
| **156** | Colchicine for Prevention of Atherothrombotic Events in Patients With Coronary Artery Disease: Review and Practical Approach for Clinicians. | remove |
| **157** | Effect of eicosapentaenoic acid/docosahexaenoic acid on coronary high-intensity plaques detected with non-contrast T1-weighted imaging (the AQUAMARINE EPA/DHA study): study protocol for a randomized controlled trial. | remove |
| **158** | Simultaneous quantitation of rosuvastatin and ezetimibe in human plasma by LC-MS/MS: Pharmacokinetic study of fixed-dose formulation and separate tablets. | remove |
| **159** | Intensive versus moderate atorvastatin therapy and one-year graft patency after CABG: Rationale and design of the ACTIVE (Aggressive Cholesterol Therapy to Inhibit Vein Graft Events) randomized controlled trial (NCT01528709). | remove |
| **160** | Effect of high-intensity statin therapy on atherosclerosis in non-infarct-related coronary arteries (IBIS-4): a serial intravascular ultrasonography study. | remove |
| **161** | Lipid-altering efficacy of ezetimibe/simvastatin 10/20 mg compared with rosuvastatin 10 mg in high-risk hypercholesterolaemic patients inadequately controlled with prior statin monotherapy - The IN-CROSS study. | remove |
| **162** | Statins effect on insulin resistance after a meal and exercise in hypercholesterolemic pre-diabetic individuals. | remove |
| **163** | Percent reduction in LDL cholesterol following high-intensity statin therapy: potential implications for guidelines and for the prescription of emerging lipid-lowering agents. | remove |
| **164** | Effect of fixed-dose combinations of ezetimibe plus rosuvastatin in patients with primary hypercholesterolemia: MRS-ROZE (Multicenter Randomized Study of ROsuvastatin and eZEtimibe). | remove |
| **165** | National Lipid Association Scientific Statement on the use of icosapent ethyl in statin-treated patients with elevated triglycerides and high or very-high ASCVD risk. | remove |
| **166** | Investigating the Lowest Threshold of Vascular Benefits from LDL Cholesterol Lowering with a PCSK9 mAb Inhibitor (Alirocumab) in Patients with Stable Cardiovascular Disease (INTENSITY-HIGH): protocol and study rationale for a randomised, open label, parallel group, mechanistic study. | remove |
| **167** | Complementary low-density lipoprotein-cholesterol lowering and pharmacokinetics of adding bempedoic acid (ETC-1002) to high-dose atorvastatin background therapy in hypercholesterolemic patients: A randomized placebo-controlled trial. | remove |
| **168** | Safety and efficacy of ezetimibe added on to rosuvastatin 5 or 10 mg versus up-titration of rosuvastatin in patients with hypercholesterolemia (the ACTE Study). | remove |
| **169** | Lipid lowering efficacy and safety of Ezetimibe combined with rosuvastatin compared with titrating rosuvastatin monotherapy in HIV-positive patients. | remove |
| **170** | A comparison of the effects of low- and high-dose atorvastatin on lipoprotein metabolism and inflammatory cytokines in type 2 diabetes: Results from the Protection Against Nephropathy in Diabetes with Atorvastatin (PANDA) randomized trial. | remove |
| **171** | Comparison of anti-inflammatory effects and high-density lipoprotein cholesterol levels between therapy with quadruple-dose rosuvastatin and rosuvastatin combined with ezetimibe. | remove |
| **172** | Implications for Ezetimibe Therapy Use Based on IMPROVE-IT Criteria. | remove |
| **173** | Comparison of Low-Dose Statin Versus Low-Dose Statin + Armolipid Plus in High-Intensity Statin-Intolerant Patients With a Previous Coronary Event and Percutaneous Coronary Intervention (ADHERENCE Trial). | remove |
| **174** | Evaluation of two highly effective lipid-lowering therapies in subjects with acute myocardial infarction. | remove |
| **175** | Lipid-Modifying Efficacy and Tolerability of Anacetrapib Added to Ongoing Statin Therapy in Patients with Hypercholesterolemia or Low High-Density Lipoprotein Cholesterol. | remove |
| **176** | Optimal medical therapy in patients with stable coronary artery disease in Poland: the ISCHEMIA trial experience. | remove |
| **177** |  | remove |
| **178** | Comparison of Pharmacokinetics and Safety of a Fixed-dose Combination of Rosuvastatin and Ezetimibe Versus Separate Tablets in Healthy Subjects. | remove |
| **179** | Lower cardiovascular mortality with atorvastatin and rosuvastatin vs simvastatin: Data from "moderate-intensity" statin users in an observational registry on chronic heart failure (Daunia Heart Failure Registry). | remove |
| **180** | Efficacy and safety of alirocumab as add-on therapy in high-cardiovascular-risk patients with hypercholesterolemia not adequately controlled with atorvastatin (20 or 40 mg) or rosuvastatin (10 or 20 mg): design and rationale of the ODYSSEY OPTIONS Studies. | remove |
| **181** | Efficacy and Safety of Lipid-Lowering Drugs of Different Intensity on Clinical Outcomes: A Systematic Review and Network Meta-Analysis. | remove |
| **182** | Prevalence and initiation of statin therapy in the oldest old-a longitudinal population-based study. | remove |
| **183** | Effect of Eicosapentaenoic Acid/Docosahexaenoic Acid on Coronary High-Intensity Plaques Detected Using Noncontrast T1-weighted Imaging: The AQUAMARINE EPA/DHA Randomized Study. | remove |
| **184** | Factors Influencing Decision Making for Carotid Endarterectomy versus Stenting in the Very Elderly. | remove |
| **185** | Lipid-altering efficacy of ezetimibe/simvastatin 10/20 mg compared to rosuvastatin 10 mg in high-risk patients with and without type 2 diabetes mellitus inadequately controlled despite prior statin monotherapy. | remove |
| **186** | Implications for Ezetimibe Therapy Use Based on IMPROVE-IT Criteria. | remove |
| **187** | Comparison of Low-Dose Statin Versus Low-Dose Statin + Armolipid Plus in High-Intensity Statin-Intolerant Patients With a Previous Coronary Event and Percutaneous Coronary Intervention (ADHERENCE Trial). | remove |
| **188** | Evaluation of two highly effective lipid-lowering therapies in subjects with acute myocardial infarction. | remove |
| **189** | Optimal medical therapy in patients with stable coronary artery disease in Poland: the ISCHEMIA trial experience. | remove |
| **190** | Comparative Muscle Tolerability of Different Types and Intensities of Statins: A Network Meta-Analysis of Double-Blind Randomized Controlled Trials. | remove |
| **191** | Comparison of Pharmacokinetics and Safety of a Fixed-dose Combination of Rosuvastatin and Ezetimibe Versus Separate Tablets in Healthy Subjects. | remove |
| **192** | Lower cardiovascular mortality with atorvastatin and rosuvastatin vs simvastatin: Data from "moderate-intensity" statin users in an observational registry on chronic heart failure (Daunia Heart Failure Registry). | remove |
| **193** | Effect of atorvastatin on lipogenic, inflammatory and thrombogenic markers in women with the metabolic syndrome. | remove |
| **194** | The effect of statin therapy on plaque regression following acute coronary syndrome: a meta-analysis of prospective trials. | remove |
| **195** | The Effect of Atorvastatin on Habitual Physical Activity among Healthy Adults. | remove |
| **196** | Effects of four antiplatelet/statin combined strategies on immune and inflammatory responses in patients with acute myocardial infarction undergoing pharmacoinvasive strategy: Design and rationale of the B and T Types of Lymphocytes Evaluation in Acute Myocardial Infarction (BATTLE-AMI) study: study protocol for a randomized controlled trial. | remove |
| **197** | The Bioequivalence and Effect of Food on the Pharmacokinetics of a Fixed-Dose Combination Tablet Containing Rosuvastatin and Ezetimibe in Healthy Japanese Subjects. | remove |
| **198** | Efficacy and Safety of Alirocumab in Adults With Homozygous Familial Hypercholesterolemia: The ODYSSEY HoFH Trial. | remove |
| **199** | Efficacy of High-Intensity Atorvastatin for Asian Patients Undergoing Percutaneous Coronary Intervention. | remove |
| **200** | Statin therapy on glycemic control in type 2 diabetic patients: A network meta-analysis. | remove |
| **201** | Effects of Alirocumab on Cardiovascular Events After Coronary Bypass Surgery. | remove |
| **202** | Effects of rosuvastatin with or without ezetimibe on clinical outcomes in patients undergoing elective vascular surgery: results of a pilot study. | remove |
| **203** | Effects of ezetimibe/simvastatin 10/10 mg versus Rosuvastatin 10 mg on carotid atherosclerotic plaque inflammation. | remove |
| **204** | Incremental cholesterol reduction with ezetimibe/simvastatin, atorvastatin and rosuvastatin in UK General Practice (IN-PRACTICE): randomised controlled trial of achievement of Joint British Societies (JBS-2) cholesterol targets. | remove |
| **205** | Baseline, delta, and achieved low-density lipoprotein cholesterol levels and cardiovascular risk in patients on statin therapy: A post-hoc resampling mediation analysis of treating new targets [TNT] trial. | remove |
| **206** | Alirocumab Reduces Total Nonfatal Cardiovascular and Fatal Events: The ODYSSEY OUTCOMES Trial. | remove |
| **207** | Medical lipid-regulating therapy: current evidence, ongoing trials and future developments. | remove |
| **208** | Effect of combination therapy of ezetimibe and rosuvastatin on regression of coronary atherosclerosis in patients with coronary artery disease. | remove |
| **209** | PCSK9 inhibition in patients with acute stroke and symptomatic intracranial atherosclerosis: protocol for a prospective, randomised, open-label, blinded end-point trial with vessel-wall MR imaging. | remove |
| **210** | Patient and physician factors influence decision-making in hypercholesterolemia: a questionnaire-based survey. | remove |
| **211** | Effect of Atorvastatin (10 mg) and Ezetimibe (10 mg) Combination Compared to Atorvastatin (40 mg) Alone on Coronary Atherosclerosis. | remove |
| **212** | Safety and Efficacy of Pitavastatin in Patients With Impaired Fasting Glucose and Hyperlipidemia: A Randomized, Open-labeled, Multicentered, Phase IV Study. | remove |
| **213** | Effect of Alirocumab on Incidence of Atrial Fibrillation After Acute Coronary Syndromes: Insights from the ODYSSEY OUTCOMES Trial. | remove |
| **214** | Low-density lipoprotein cholesterol reduction and goal achievement with ezetimibe/simvastatin versus atorvastatin or rosuvastatin in patients with diabetes, metabolic syndrome, or neither disease, stratified by National Cholesterol Education Program risk category. | remove |
| **215** | Low-density lipoprotein cholesterol reduction and goal achievement with ezetimibe/simvastatin versus atorvastatin or rosuvastatin in patients with diabetes, metabolic syndrome, or neither disease, stratified by National Cholesterol Education Program risk category. | remove |
| **216** | Intensive lipid intervention in the post-ENHANCE era. | remove |
| **217** | Coronary atheroma progression rates in men and women following high-intensity statin therapy: A pooled analysis of REVERSAL, ASTEROID and SATURN. | remove |
| **218** | Effect of Statin Treatment on Modifying Plaque Composition: A Double-Blind, Randomized Study. | remove |
| **219** | Comparative effects on lipid levels of combination therapy with a statin and extended-release niacin or ezetimibe versus a statin alone (the COMPELL study). | remove |
| **220** | Effect of intensive lipid-lowering therapies on cholinesterase activity in patients with coronary artery disease. | remove |
| **221** | Pharmacokinetic and bioequivalence study of a fixed-dose combination of amlodipine besylate and rosuvastatin calcium compared to co-administration of separate tablets in healthy Korean subjects. | remove |
| **222** | Detrimental effects of high-fat diet loading on vascular endothelial function and therapeutic efficacy of ezetimibe and statins in patients with type 2 diabetes. | remove |
| **223** | Rosuvastatin for Reduction of Myocardial Damage during Coronary Angioplasty - the Remedy Trial. | remove |
| **224** | Efficacy of combination of Ezetimibe 10 mg and rosuvastatin 2.5 mg versus rosuvastatin 5 mg monotherapy for hypercholesterolemia in patients with type 2 diabetes. | remove |
| **225** | Effectiveness of Roux-en-Y Gastric Bypass vs Sleeve Gastrectomy on Lipid Levels in Type 2 Diabetes: a Meta-analysis. | remove |
| **226** | Group IIA Secretory Phospholipase A2, Vascular Inflammation, and Incident Cardiovascular Disease. | remove |
| **227** | Comparison of the Effect of Rosuvastatin 2.5 mg vs 20 mg on Coronary Plaque Determined by Angioscopy and Intravascular Ultrasound in Japanese With Stable Angina Pectoris (from the Aggressive Lipid-Lowering Treatment Approach Using Intensive Rosuvastatin for Vulnerable Coronary Artery Plaque [ALTAIR] Randomized Trial). | remove |
| **228** | Prolonged-release nicotinic acid for the management of dyslipidemia: an update including results from the NAUTILUS study. | remove |
| **229** | Efficacy and safety of alirocumab in patients with or without prior coronary revascularization: Pooled analysis of eight ODYSSEY phase 3 trials. | remove |
| **230** | Lipid-altering efficacy of switching to ezetimibe/simvastatin 10/20 mg versus rosuvastatin 10 mg in high-risk patients with and without metabolic syndrome. | remove |
| **231** | Main differences between two highly effective lipid-lowering therapies in subclasses of lipoproteins in patients with acute myocardial infarction. | remove |
| **232** | Main differences between two highly effective lipid-lowering therapies in subclasses of lipoproteins in patients with acute myocardial infarction. | remove |
| **233** | Effects of 4 Statins on Regression of Coronary Plaque in Acute Coronary Syndrome. | remove |
| **234** | To what extent do high-intensity statins reduce low-density lipoprotein cholesterol in each of the four statin benefit groups identified by the 2013 American College of Cardiology/American Heart Association guidelines? A VOYAGER meta-analysis. | remove |
| **235** | Effect of Statin Therapy on Outcomes of Patients With Acute Ischemic Stroke and Atrial Fibrillation. | remove |
| **236** | Pharmacological Inhibition of CETP (Cholesteryl Ester Transfer Protein) Increases HDL (High-Density Lipoprotein) That Contains ApoC3 and Other HDL Subspecies Associated With Higher Risk of Coronary Heart Disease. | remove |
| **237** | Effect of Eicosapentaenoic and Docosahexaenoic Acids Added to Statin Therapy on Coronary Artery Plaque in Patients With Coronary Artery Disease: A Randomized Clinical Trial. | remove |
| **238** | Evolocumab treatment in patients with HIV and hypercholesterolemia/mixed dyslipidemia: BEIJERINCK study design and baseline characteristics. | remove |
| **239** | Associations between lower levels of low-density lipoprotein cholesterol and cardiovascular events in very high-risk patients: Pooled analysis of nine ODYSSEY trials of alirocumab versus control. | remove |
| **240** | Coronary atheroma volume and cardiovascular events during maximally intensive statin therapy. | remove |
| **241** | Effects of atorvastatin 20 mg, rosuvastatin 10 mg, and atorvastatin/ezetimibe 5 mg/5 mg on lipoproteins and glucose metabolism. | remove |
| **242** | Additive effects of plant sterols supplementation in addition to different lipid-lowering regimens. | remove |
| **243** | Changes in lipoproteins associated with lipid-lowering and antiplatelet strategies in patients with acute myocardial infarction. | remove |
| **244** | Modeling Statin-Induced Reductions of Cardiovascular Events in Primary Prevention: A VOYAGER Meta-Analysis. | remove |
| **245** | Comparison of the effect of simvastatin versus simvastatin/ezetimibe versus rosuvastatin on markers of inflammation and oxidative stress in subjects with hypercholesterolemia. | remove |
| **246** | The efficacy and safety of ezetimibe/simvastatin combination compared with intensified lipid-lowering treatment strategies in diabetic subjects with and without metabolic syndrome. | remove |
| **247** | Ticagrelor improves blood viscosity-dependent microcirculatory flow in patients with lower extremity arterial disease: the Hema-kinesis clinical trial. | remove |
| **248** | Coordinating Cardiology clinics randomized trial of interventions to improve outcomes (COORDINATE) - Diabetes: rationale and design. | remove |
| **249** | Effect of atorvastatin on muscle symptoms in coronary heart disease patients with self-perceived statin muscle side effects: a randomized, double-blinded crossover trial. | remove |
| **250** | Lipid Screening in Childhood and Adolescence for Detection of Familial Hypercholesterolemia: A Systematic Evidence Review for the U.S. Preventive Services Task Force [Internet]. | remove |
| **251** | Long-term effects of ezetimibe-plus-statin therapy on low-density lipoprotein cholesterol levels as compared with double-dose statin therapy in patients with coronary artery disease. | remove |
| **252** | Apabetalone and hospitalization for heart failure in patients following an acute coronary syndrome: a prespecified analysis of the BETonMACE study. | remove |
| **253** | Effects of the PCSK9 antibody alirocumab on coronary atherosclerosis in patients with acute myocardial infarction: a serial, multivessel, intravascular ultrasound, near-infrared spectroscopy and optical coherence tomography imaging study-Rationale and design of the PACMAN-AMI trial. | remove |
| **254** | Development and Validation of a Model to Predict Absolute Vascular Risk Reduction by Moderate-Intensity Statin Therapy in Individual Patients With Type 2 Diabetes Mellitus: The Anglo Scandinavian Cardiac Outcomes Trial, Antihypertensive and Lipid-Lowering Treatment to Prevent Heart Attack Trial, and Collaborative Atorvastatin Diabetes Study. | remove |
| **255** | Efficacy and safety of gemcabene as add-on to stable statin therapy in hypercholesterolemic patients. | remove |
| **256** | Cost-Effectiveness of Alirocumab in Patients With Acute Coronary Syndromes: The ODYSSEY OUTCOMES Trial. | remove |
| **257** | Efficacy and safety of gemcabene as add-on to stable statin therapy in hypercholesterolemic patients. | remove |
| **258** | A Comparison of Statin Therapies in Hypercholesterolemia in Women: A Subgroup Analysis of the STELLAR Study. | remove |
| **259** | In CAD, treat-to-target statins were noninferior to high-intensity statins for a composite clinical outcome. | remove |
| **260** | Effect of Statin Use on Mobility Disability and its Prevention in At-risk Older Adults: The LIFE Study. | remove |
| **261** | Impact of statin therapy intensity on endothelial progenitor cells after percutaneous coronary intervention in diabetic patients. The REMEDY-EPC late study. | remove |
| **262** | Intensive statin therapy, used alone or in combination with ezetimibe, improves homocysteine level and lipid peroxidation to a similar degree in patients with coronary artery diseases. | remove |
| **263** | Rationale and design of the Women's Ischemia Trial to Reduce Events in Nonobstructive CAD (WARRIOR) trial. | remove |
| **264** | Effect of the PCSK9 Inhibitor Evolocumab on Total Cardiovascular Events in Patients With Cardiovascular Disease: A Prespecified Analysis From the FOURIER Trial. | remove |
| **265** | Effects of rosuvastatin vs. simvastatin/ezetimibe on arterial wall stiffness in patients with coronary artery disease. | remove |
| **266** | Differences in synthesis and absorption of cholesterol of two effective lipid-lowering therapies. | remove |
| **267** | Relative efficacy of antilipemic agents in non–high-density lipoprotein cholesterol reduction. | remove |
| **268** | LEGACY: Phase 2a Trial to Evaluate the Safety, Pharmacokinetics, and Pharmacodynamic Effects of the Anti-EL (Endothelial Lipase) Antibody MEDI5884 in Patients With Stable Coronary Artery Disease. | remove |
| **269** | Comparative effect of physical exercise versus statins on improving arterial stiffness in patients with high cardiometabolic risk: A network meta-analysis. | remove |
| **270** | Assessment of the Efficacy of Lowering LDL Cholesterol with Rosuvastatin 10 mg in Four Korean Statin Benefit Groups as per ACC/AHA Guidelines (NewStaR4G). | remove |
| **271** | Alirocumab as Add-On to Atorvastatin Versus Other Lipid Treatment Strategies: ODYSSEY OPTIONS I Randomized Trial. | remove |
| **272** | Alirocumab Reduces Total Hospitalizations and Increases Days Alive and Out of Hospital in the ODYSSEY OUTCOMES Trial. | remove |
| **273** | Lipid-altering efficacy of the ezetimibe/simvastatin single tablet versus rosuvastatin in hypercholesterolemic patients. | remove |
| **274** | Effects of two lipid lowering therapies on immune responses in hyperlipidemic subjects. | remove |
| **275** | Alirocumab in Acute Myocardial Infarction: Results From the Virginia Commonwealth University Alirocumab Response Trial (VCU-AlirocRT). | remove |
| **276** | Alirocumab in Acute Myocardial Infarction: Results From the Virginia Commonwealth University Alirocumab Response Trial (VCU-AlirocRT). | remove |
| **277** | Effect of Switching from Low-Dose Simvastatin to High-Dose Atorvastatin on Glucose Homeostasis and Cognitive Function in Type 2 Diabetes. | remove |
| **278** | Effect of intensive versus moderate lipid-lowering therapy on epicardial adipose tissue in hyperlipidemic post-menopausal women: a substudy of the BELLES trial (Beyond Endorsed Lipid Lowering with EBT Scanning). | remove |
| **279** | Investigation of Motivational Interviewing and Prevention Consults to Achieve Cardiovascular Targets (IMPACT) trial. | remove |
| **280** | Combining multiple approaches for the secondary prevention of vascular events after stroke: a quantitative modeling study. | remove |
| **281** | Improvement in Renal Function and Reduction in Serum Uric Acid with Intensive Statin Therapy in Older Patients: A Post Hoc Analysis of the SAGE Trial. | remove |
| **282** | A Meta-Analysis Assessing Additional LDL-C Reduction from Addition of a Bile Acid Sequestrant to Statin Therapy. | remove |
| **283** |  | remove |
| **284** | A Meta-Analysis Assessing Additional LDL-C Reduction from Addition of a Bile Acid Sequestrant to Statin Therapy. | remove |
| **285** | Cost-effectiveness of Simvastatin plus Ezetimibe for Cardiovascular Prevention in CKD: Results of the Study of Heart and Renal Protection (SHARP). | remove |
| **286** | The role of soluble fiber intake in patients under highly effective lipid-lowering therapy. | remove |
| **287** | The role of soluble fiber intake in patients under highly effective lipid-lowering therapy. | remove |
| **288** | Disappointing recent cholesterol-lowering drug trials: is it not time for a full reappraisal of the cholesterol theory? | remove |
| **289** | Disappointing recent cholesterol-lowering drug trials: is it not time for a full reappraisal of the cholesterol theory? | remove |
| **290** | [Comparative Randomized Study of the Effects of Long-Term Therapy With Rosuvastatin and Combination of Atorvastatin and Ezetimibe on Carbohydrate Metabolism and Adipokines Levels in Patients With Coronary Artery Disease and Diabetes Mellitus]. | remove |
| **291** | [Comparative Randomized Study of the Effects of Long-Term Therapy With Rosuvastatin and Combination of Atorvastatin and Ezetimibe on Carbohydrate Metabolism and Adipokines Levels in Patients With Coronary Artery Disease and Diabetes Mellitus]. | remove |
| **292** | Comparison of the effects of simvastatin vs. rosuvastatin vs. simvastatin/ezetimibe on parameters of insulin resistance. | remove |
| **293** | Efficacy and safety of evolocumab in individuals with type 2 diabetes mellitus: primary results of the randomised controlled BANTING study. | remove |
| **294** | Consistency of effect of ezetimibe/simvastatin compared with intensified lipid-lowering treatment strategies in obese and non-obese diabetic subjects. | remove |
| **295** | Clinical usefulness of additional treatment with ezetimibe in patients with coronary artery disease on statin therapy. - From the viewpoint of cholesterol metabolism.-. | remove |
| **296** | Clinical usefulness of additional treatment with ezetimibe in patients with coronary artery disease on statin therapy. - From the viewpoint of cholesterol metabolism.-. | remove |
| **297** | Antiatherosclerotic effects of long-term maximally intensive statin therapy after acute coronary syndrome: insights from Study of Coronary Atheroma by Intravascular Ultrasound: Effect of Rosuvastatin Versus Atorvastatin. | remove |
| **298** | Prediction of cardiovascular event risk reduction from lipid changes associated with high potency dyslipidemia therapy. | remove |
| **299** | Prediction of cardiovascular event risk reduction from lipid changes associated with high potency dyslipidemia therapy. | remove |
| **300** | Three-dimensional morphological response of lipid-rich coronary plaques to statin therapy: a serial optical coherence tomography study. | remove |
| **301** | Safety profile of subjects treated to very low low-density lipoprotein cholesterol levels (<30 mg/dl) with rosuvastatin 20 mg daily (from JUPITER). | remove |
| **302** | [New AHA and ACC guidelines on the treatment of blood cholesterol to reduce atherosclerotic cardiovascular risk : Statement of the D•A•CH Society for Prevention of Cardiovascular Diseases, the Austrian Atherosclerosis Society and the Working Group on Lipids and Atherosclerosis (AGLA) of the Swiss Society for Cardiology]. | remove |
| **303** | [Effects of different statins, ezetimibe/simvastatin combination on hsCRP levels in unstable angina pectoris and non-ST elevation myocardial infarction patients: a randomized trial]. | remove |
| **304** | [Effects of different statins, ezetimibe/simvastatin combination on hsCRP levels in unstable angina pectoris and non-ST elevation myocardial infarction patients: a randomized trial]. | remove |
| **305** | A 1-year lifestyle intervention for weight loss in individuals with type 2 diabetes reduces high C-reactive protein levels and identifies metabolic predictors of change: from the Look AHEAD (Action for Health in Diabetes) study. | remove |
| **306** | A 1-year lifestyle intervention for weight loss in individuals with type 2 diabetes reduces high C-reactive protein levels and identifies metabolic predictors of change: from the Look AHEAD (Action for Health in Diabetes) study. | remove |
| **307** | Concordance between plasma apolipoprotein B levels and cholesterol indices among patients receiving statins and nonstatin treatment: Post-hoc analyses from the U.K. InPractice study. | remove |
| **308** | Serial Optical Coherence Tomography and Intravascular Ultrasound Analysis of Gender Difference in Changes of Plaque Phenotype in Response to Lipid-Lowering Therapy. | remove |
| **309** | Rosuvastatin increased serum osteocalcin levels independent of its serum cholesterol-lowering effect in patients with type 2 diabetes and hypercholesterolemia. | remove |
| **310** | Rosuvastatin increased serum osteocalcin levels independent of its serum cholesterol-lowering effect in patients with type 2 diabetes and hypercholesterolemia. | remove |
| **311** | The effect of lowering LDL cholesterol on vascular access patency: post hoc analysis of the Study of Heart and Renal Protection. | remove |
| **312** | Long-term efficacy and safety of moderate-intensity statin with ezetimibe combination therapy versus high-intensity statin monotherapy in patients with atherosclerotic cardiovascular disease (RACING): a randomised, open-label, non-inferiority trial. | remove |
| **313** | A Phase III, Multicenter, Randomized, Double-blind, Active Comparator Clinical Trial to Compare the Efficacy and Safety of Combination Therapy With Ezetimibe and Rosuvastatin Versus Rosuvastatin Monotherapy in Patients With Hypercholesterolemia: I-ROSETTE (Ildong Rosuvastatin & Ezetimibe for Hypercholesterolemia) Randomized Controlled Trial. | remove |
| **314** | The Efficacy and Safety of Moderate-Intensity Rosuvastatin with Ezetimibe versus High-Intensity Rosuvastatin in High Atherosclerotic Cardiovascular Disease Risk Patients with Type 2 Diabetes Mellitus: A Randomized, Multicenter, Open, Parallel, Phase 4 Study. | remove |
| **315** | A Randomized, Multicenter, Double-blind, Placebo-Controlled Study to Evaluate the Efficacy and Safety of a Quadruple Combination of Amlodipine, Losartan, Rosuvastatin, and Ezetimibe in Patients with Concomitant Essential Hypertension and Dyslipidemia. | remove |
| **316** | Pharmacokinetic Interaction Among Ezetimibe, Rosuvastatin, and Telmisartan. | remove |
| **317** | Pharmacokinetic Interaction Between Telmisartan and Rosuvastatin/Ezetimibe After Multiple Oral Administration in Healthy Subjects. | remove |
| **318** | Ezetimibe Improves Rosuvastatin Effects on Inflammation and Vascular Endothelial Function in Acute Coronary Syndrome Patients Undergoing PCI. | remove |
| **319** | Ezetimibe and Rosuvastatin Combination Treatment Can Reduce the Dose of Rosuvastatin Without Compromising Its Lipid-lowering Efficacy. | remove |
| **320** | Pharmacokinetic Interactions and Tolerability of Rosuvastatin and Ezetimibe: A Randomized, Phase 1, Crossover Study in Healthy Chinese Participants. | remove |
| **321** | Pharmacokinetic and pharmacodynamic interaction between ezetimibe and rosuvastatin in healthy male subjects. | remove |
| **322** | Efficacy and Safety of Ezetimibe and Rosuvastatin Combination Therapy Versus Those of Rosuvastatin Monotherapy in Patients With Primary Hypercholesterolemia. | remove |
| **323** | Efficacy and Safety of Single-Pill Combination of Rosuvastatin and Ezetimibe in Chinese Patients with Primary Hypercholesterolemia Inadequately Controlled by Statin Treatment (ROZEL): A Randomized, Double-Blind, Double Dummy, Active-Controlled Phase 3 Clinical Trial. | remove |
| **324** | Combination Therapy of Rosuvastatin and Ezetimibe in Patients with High Cardiovascular Risk. | remove |
| **325** | Effect of rosuvastatin 20 mg versus rosuvastatin 5 mg plus ezetimibe on statin side-effects in elderly patients with atherosclerotic cardiovascular disease: Rationale and design of a randomized, controlled SaveSAMS trial. | remove |
| **326** | Ezetimibe combination therapy with statin for non-alcoholic fatty liver disease: an open-label randomized controlled trial (ESSENTIAL study). | remove |
| **327** | Low dose of ROSuvastatin in combination with EZEtimibe effectively and permanently reduce low density lipoprotein cholesterol concentration independently of timing of administration (ROSEZE): A randomized, crossover study - preliminary results. | remove |
| **328** | Moderate-Intensity Statin With Ezetimibe Combination Therapy vs High-Intensity Statin Monotherapy in Patients at Very High Risk of Atherosclerotic Cardiovascular Disease: A Post Hoc Analysis From the RACING Randomized Clinical Trial. | remove |
| **329** | Pharmacokinetics and bioequivalence of a rosuvastatin/ezetimibe fixed-dose combination tablet versus single agents in healthy male subjects. | remove |
| **330** | Effects of Fixed-dose Combination of Low-intensity Rosuvastatin and Ezetimibe Versus Moderate-intensity Rosuvastatin Monotherapy on Lipid Profiles in Patients With Hypercholesterolemia: A Randomized, Double-blind, Multicenter, Phase III Study. | remove |
| **331** | Pharmacokinetic interactions and tolerability of rosuvastatin and ezetimibe: an open-label, randomized, multiple-dose, crossover study in healthy male volunteers. | remove |
| **332** | Efficacy and Safety of Ezetimibe and Rosuvastatin Combination Therapy Versus Those of Rosuvastatin Monotherapy in Patients With Primary Hypercholesterolemia. | remove |
| **333** | Effects of Combination of Ezetimibe and Rosuvastatin on Coronary Artery Plaque in Patients with Coronary Heart Disease. | remove |
| **334** | Pharmacodynamic interaction between ezetimibe and rosuvastatin. | remove |
| **335** | The Bioequivalence and Effect of Food on the Pharmacokinetics of a Fixed-Dose Combination Tablet Containing Rosuvastatin and Ezetimibe in Healthy Japanese Subjects. | remove |
| **336** | A Phase 3 Randomized Controlled Trial to Evaluate Efficacy and Safety of New-Formulation Zenon (Rosuvastatin/Ezetimibe Fixed-Dose Combination) in Primary Hypercholesterolemia Inadequately Controlled by Statins. | remove |
| **337** | Simultaneous quantitation of rosuvastatin and ezetimibe in human plasma by LC-MS/MS: Pharmacokinetic study of fixed-dose formulation and separate tablets. | remove |
| **338** | Comparison of Pharmacokinetics and Safety of a Fixed-dose Combination of Rosuvastatin and Ezetimibe Versus Separate Tablets in Healthy Subjects. | remove |
| **339** | Lipid lowering efficacy and safety of Ezetimibe combined with rosuvastatin compared with titrating rosuvastatin monotherapy in HIV-positive patients. | remove |
| **340** | The impact of the time of drug administration on the effectiveness of combined treatment of hypercholesterolemia with Rosuvastatin and Ezetimibe (RosEze): study protocol for a randomized controlled trial. | remove |
| **341** | Efficacy and safety of adding alirocumab to rosuvastatin versus adding ezetimibe or doubling the rosuvastatin dose in high cardiovascular-risk patients: The ODYSSEY OPTIONS II randomized trial. | remove |
| **342** | Efficacy and safety of rosuvastatin 40 mg alone or in combination with ezetimibe in patients at high risk of cardiovascular disease (results from the EXPLORER study). | remove |
| **343** | A randomized, controlled comparison of different intensive lipid-lowering therapies in Chinese patients with non-ST-elevation acute coronary syndrome (NSTE-ACS): Ezetimibe and rosuvastatin versus high-dose rosuvastatin. | remove |
| **344** | Efficacy and safety of coadministration of rosuvastatin, ezetimibe, and colestimide in heterozygous familial hypercholesterolemia. | remove |
| **345** | Safety and effectiveness of the association ezetimibe-statin (E-S) versus high dose rosuvastatin after acute coronary syndrome: the SAFE-ES study. | remove |
| **346** | Effect of fixed-dose combinations of ezetimibe plus rosuvastatin in patients with primary hypercholesterolemia: MRS-ROZE (Multicenter Randomized Study of ROsuvastatin and eZEtimibe). | remove |
| **347** | Safety and efficacy of ezetimibe added on to rosuvastatin 5 or 10 mg versus up-titration of rosuvastatin in patients with hypercholesterolemia (the ACTE Study). | remove |
| **348** | Efficacy of combination of Ezetimibe 10 mg and rosuvastatin 2.5 mg versus rosuvastatin 5 mg monotherapy for hypercholesterolemia in patients with type 2 diabetes. | remove |
| **349** | Effects of Ezetimibe/Simvastatin and Rosuvastatin on Oxidative Stress in Diabetic Neuropathy: A Randomized, Double-Blind, Placebo-Controlled Clinical Trial. | remove |
| **350** | Efficacy and safety of ezetimibe added to atorvastatin versus atorvastatin uptitration or switching to rosuvastatin in patients with primary hypercholesterolemia. | remove |
| **351** | Comparison of the effect of simvastatin versus simvastatin/ezetimibe versus rosuvastatin on markers of inflammation and oxidative stress in subjects with hypercholesterolemia. | remove |
| **352** | Efficacy, safety and effect on biomarkers related to cholesterol and lipoprotein metabolism of rosuvastatin 10 or 20 mg plus ezetimibe 10 mg vs. simvastatin 40 or 80 mg plus ezetimibe 10 mg in high-risk patients: Results of the GRAVITY randomized study. | remove |
| **353** | Comparison of anti-inflammatory effects and high-density lipoprotein cholesterol levels between therapy with quadruple-dose rosuvastatin and rosuvastatin combined with ezetimibe. | remove |
| **354** | Effect of combination therapy of ezetimibe and rosuvastatin on regression of coronary atherosclerosis in patients with coronary artery disease. | remove |
| **355** | A Bioequivalence Study of Ezetimibe/Rosuvastatin Fixed Dose Combination (10 mg/10 mg) Versus the Individual Formulations Taken Concomitantly. | remove |
| **356** | Incremental cholesterol reduction with ezetimibe/simvastatin, atorvastatin and rosuvastatin in UK General Practice (IN-PRACTICE): randomised controlled trial of achievement of Joint British Societies (JBS-2) cholesterol targets. | remove |
| **357** | Effects of atorvastatin 20 mg, rosuvastatin 10 mg, and atorvastatin/ezetimibe 5 mg/5 mg on lipoproteins and glucose metabolism. | remove |
| **358** | Comparison of High-Dose Rosuvastatin Versus Low-Dose Rosuvastatin Plus Ezetimibe on Carotid Atherosclerotic Plaque Inflammation in Patients with Acute Coronary Syndrome. | remove |
| **359** | Lipid-altering efficacy of switching to ezetimibe/simvastatin 10/20 mg versus rosuvastatin 10 mg in high-risk patients with and without metabolic syndrome. | remove |
| **360** | Switching from statin monotherapy to ezetimibe/simvastatin or rosuvastatin modifies the relationships between apolipoprotein B, LDL cholesterol, and non-HDL cholesterol in patients at high risk of coronary disease. | remove |
| **361** | The influence of statin monotherapy and statin-ezetimibe combined therapy on FoxP3 and IL 10 mRNA expression in patients with coronary artery disease. | remove |
| **362** | Effects of ezetimibe/simvastatin 10/10 mg versus Rosuvastatin 10 mg on carotid atherosclerotic plaque inflammation. | remove |
| **363** | Efficacy of Rosuvastatin in Children With Homozygous Familial Hypercholesterolemia and Association With Underlying Genetic Mutations. | remove |
| **364** | Moderate-intensity statin plus ezetimibe vs high-intensity statin according to baseline LDL-C in the treatment of atherosclerotic cardiovascular disease: A post-hoc analysis of the RACING randomized trial. | remove |
| **365** | [Comparative Randomized Study of the Effects of Long-Term Therapy With Rosuvastatin and Combination of Atorvastatin and Ezetimibe on Carbohydrate Metabolism and Adipokines Levels in Patients With Coronary Artery Disease and Diabetes Mellitus]. | remove |
| **366** | The efficacy and safety of ezetimibe/simvastatin combination compared with intensified lipid-lowering treatment strategies in diabetic subjects with and without metabolic syndrome. | remove |
| **367** | Lipid-altering efficacy of ezetimibe/simvastatin 10/20 mg compared to rosuvastatin 10 mg in high-risk patients with and without type 2 diabetes mellitus inadequately controlled despite prior statin monotherapy. | remove |
| **368** | Rosuvastatin but not ezetimibe improves endothelial function in patients with heart failure, by mechanisms independent of lipid lowering. | remove |
| **369** | Low-density lipoprotein cholesterol reduction and goal achievement with ezetimibe/simvastatin versus atorvastatin or rosuvastatin in patients with diabetes, metabolic syndrome, or neither disease, stratified by National Cholesterol Education Program risk category. | remove |
| **370** | Detrimental effects of high-fat diet loading on vascular endothelial function and therapeutic efficacy of ezetimibe and statins in patients with type 2 diabetes. | remove |
| **371** | Effects of rosuvastatin vs. simvastatin/ezetimibe on arterial wall stiffness in patients with coronary artery disease. | remove |
| **372** | Effect of evolocumab or ezetimibe added to moderate- or high-intensity statin therapy on LDL-C lowering in patients with hypercholesterolemia: the LAPLACE-2 randomized clinical trial. | remove |
| **373** | Lipid-altering efficacy of the ezetimibe/simvastatin single tablet versus rosuvastatin in hypercholesterolemic patients. | remove |
| **374** | The effect of adding ezetimibe to rosuvastatin on renal function in patients undergoing elective vascular surgery. | remove |
| **375** | Consistency of effect of ezetimibe/simvastatin compared with intensified lipid-lowering treatment strategies in obese and non-obese diabetic subjects. | remove |
| **376** | Efficacy and safety of alirocumab as add-on therapy in high-cardiovascular-risk patients with hypercholesterolemia not adequately controlled with atorvastatin (20 or 40 mg) or rosuvastatin (10 or 20 mg): design and rationale of the ODYSSEY OPTIONS Studies. | remove |
| **377** | Comparison of the effects of simvastatin vs. rosuvastatin vs. simvastatin/ezetimibe on parameters of insulin resistance. | remove |
| **378** | Changes in lipoproteins associated with lipid-lowering and antiplatelet strategies in patients with acute myocardial infarction. | remove |
| **379** | A comparison of efficacy and safety of an ezetimibe/simvastatin combination compared with other intensified lipid-lowering treatment strategies in diabetic patients with symptomatic cardiovascular disease. | remove |
| **380** | Comparative effects on lipid levels of combination therapy with a statin and extended-release niacin or ezetimibe versus a statin alone (the COMPELL study). | remove |
| **381** | Differences in synthesis and absorption of cholesterol of two effective lipid-lowering therapies. | remove |
| **382** | Ezetimibe/simvastatin 10/20 mg versus rosuvastatin 10 mg in high-risk hypercholesterolemic patients stratified by prior statin treatment potency. | remove |
| **383** | Effect of intensive lipid-lowering therapies on cholinesterase activity in patients with coronary artery disease. | remove |
| **384** | Changes in lipoprotein lipase and endothelial lipase mass in familial hypercholesterolemia during three-drug lipid-lowering combination therapy. | remove |
| **385** | Effects of rosuvastatin with or without ezetimibe on clinical outcomes in patients undergoing elective vascular surgery: results of a pilot study. | remove |
| **386** | Effects of statin monotherapy versus statin plus ezetimibe combination on serum uric acid levels. | remove |
| **387** | Intensive statin therapy, used alone or in combination with ezetimibe, improves homocysteine level and lipid peroxidation to a similar degree in patients with coronary artery diseases. | remove |
| **388** | Alirocumab as Add-On to Atorvastatin Versus Other Lipid Treatment Strategies: ODYSSEY OPTIONS I Randomized Trial. | remove |
| **389** | [Effects of different statins, ezetimibe/simvastatin combination on hsCRP levels in unstable angina pectoris and non-ST elevation myocardial infarction patients: a randomized trial]. | remove |
| **390** | Evaluation of two highly effective lipid-lowering therapies in subjects with acute myocardial infarction. | remove |
| **391** | Rosuvastatin increased serum osteocalcin levels independent of its serum cholesterol-lowering effect in patients with type 2 diabetes and hypercholesterolemia. | remove |
| **392** | Rosuvastatin for Reduction of Myocardial Damage during Coronary Angioplasty - the Remedy Trial. | remove |
| **393** | Patient and physician factors influence decision-making in hypercholesterolemia: a questionnaire-based survey. | remove |
| **394** | Effects of four antiplatelet/statin combined strategies on immune and inflammatory responses in patients with acute myocardial infarction undergoing pharmacoinvasive strategy: Design and rationale of the B and T Types of Lymphocytes Evaluation in Acute Myocardial Infarction (BATTLE-AMI) study: study protocol for a randomized controlled trial. | remove |
| **395** | Lipid-altering efficacy of ezetimibe/simvastatin 10/20 mg compared with rosuvastatin 10 mg in high-risk hypercholesterolaemic patients inadequately controlled with prior statin monotherapy - The IN-CROSS study. | remove |
| **396** | Main differences between two highly effective lipid-lowering therapies in subclasses of lipoproteins in patients with acute myocardial infarction. | remove |
| **397** | Effects of two lipid lowering therapies on immune responses in hyperlipidemic subjects. | remove |
| **398** | Influence of intensive lipid-lowering on CT derived fractional flow reserve in patients with stable chest pain: Rationale and design of the FLOWPROMOTE study. | remove |
| **399** | Clinical usefulness of additional treatment with ezetimibe in patients with coronary artery disease on statin therapy. - From the viewpoint of cholesterol metabolism.-. | remove |
| **400** | Effect of Switching From Statin Monotherapy to Ezetimibe/Simvastatin Combination Therapy Compared With Other Intensified Lipid-Lowering Strategies on Lipoprotein Subclasses in Diabetic Patients With Symptomatic Cardiovascular Disease. | remove |
| **401** | Long-term effects of ezetimibe-plus-statin therapy on low-density lipoprotein cholesterol levels as compared with double-dose statin therapy in patients with coronary artery disease. | remove |
| **402** | The role of soluble fiber intake in patients under highly effective lipid-lowering therapy. | remove |
| **403** | The acute impact of high-dose lipid-lowering treatment on endothelial progenitor cells in patients with coronary artery disease-The REMEDY-EPC early substudy. | remove |
| **404** | Concordance between plasma apolipoprotein B levels and cholesterol indices among patients receiving statins and nonstatin treatment: Post-hoc analyses from the U.K. InPractice study. | remove |
| **405** | The effect of lowering LDL cholesterol on vascular access patency: post hoc analysis of the Study of Heart and Renal Protection. | remove |
| **406** | Long-term efficacy and safety of moderate-intensity statin with ezetimibe combination therapy versus high-intensity statin monotherapy in patients with atherosclerotic cardiovascular disease (RACING): a randomised, open-label, non-inferiority trial. | remove |
| **407** | Combination Moderate-Intensity Statin and Ezetimibe Therapy for Elderly Patients With Atherosclerosis. | remove |
| **408** | Moderate-intensity statin with ezetimibe vs. high-intensity statin in patients with diabetes and atherosclerotic cardiovascular disease in the RACING trial. | remove |
| **409** | Moderate-Intensity Statin With Ezetimibe Combination Therapy vs High-Intensity Statin Monotherapy in Patients at Very High Risk of Atherosclerotic Cardiovascular Disease: A Post Hoc Analysis From the RACING Randomized Clinical Trial. | remove |
| **410** | The Efficacy and Safety of Moderate-Intensity Rosuvastatin with Ezetimibe versus High-Intensity Rosuvastatin in High Atherosclerotic Cardiovascular Disease Risk Patients with Type 2 Diabetes Mellitus: A Randomized, Multicenter, Open, Parallel, Phase 4 Study. | remove |
| **411** | Moderate-intensity statin plus ezetimibe vs high-intensity statin according to baseline LDL-C in the treatment of atherosclerotic cardiovascular disease: A post-hoc analysis of the RACING randomized trial. | remove |
| **412** | Effectiveness of low-intensity atorvastatin 5 mg and ezetimibe 10 mg combination therapy compared with moderate-intensity atorvastatin 10 mg monotherapy: A randomized, double-blinded, multi-center, phase III study. | remove |
| **413** | Effect of evolocumab or ezetimibe added to moderate- or high-intensity statin therapy on LDL-C lowering in patients with hypercholesterolemia: the LAPLACE-2 randomized clinical trial. | remove |
| **414** | Medium-intensity statin with ezetimibe versus high-intensity statin in acute ischemic cerebrovascular disease (MESIA): A randomized clinical trial. | remove |
| **415** | Effects of Fixed-dose Combination of Low-intensity Rosuvastatin and Ezetimibe Versus Moderate-intensity Rosuvastatin Monotherapy on Lipid Profiles in Patients With Hypercholesterolemia: A Randomized, Double-blind, Multicenter, Phase III Study. | remove |
| **416** | Effect of Atorvastatin (10 mg) and Ezetimibe (10 mg) Combination Compared to Atorvastatin (40 mg) Alone on Coronary Atherosclerosis. | remove |
| **417** | Efficacy and safety of gemcabene as add-on to stable statin therapy in hypercholesterolemic patients. | remove |
| **418** | Long-term efficacy and safety of moderate-intensity statin with ezetimibe combination therapy versus high-intensity statin monotherapy in patients with atherosclerotic cardiovascular disease (RACING): a randomised, open-label, non-inferiority trial. | remove |
| **419** | Moderate-intensity statin with ezetimibe vs. high-intensity statin in patients with diabetes and atherosclerotic cardiovascular disease in the RACING trial. | remove |
| **420** | Combination Moderate-Intensity Statin and Ezetimibe Therapy for Elderly Patients With Atherosclerosis. | remove |
| **421** | Bempedoic acid plus ezetimibe fixed-dose combination in patients with hypercholesterolemia and high CVD risk treated with maximally tolerated statin therapy. | remove |
| **422** | Moderate-Intensity Statin With Ezetimibe Combination Therapy vs High-Intensity Statin Monotherapy in Patients at Very High Risk of Atherosclerotic Cardiovascular Disease: A Post Hoc Analysis From the RACING Randomized Clinical Trial. | remove |
| **423** | Obicetrapib plus ezetimibe as an adjunct to high-intensity statin therapy: A randomized phase 2 trial. | remove |
| **424** | The Efficacy and Safety of Moderate-Intensity Rosuvastatin with Ezetimibe versus High-Intensity Rosuvastatin in High Atherosclerotic Cardiovascular Disease Risk Patients with Type 2 Diabetes Mellitus: A Randomized, Multicenter, Open, Parallel, Phase 4 Study. | remove |
| **425** | Moderate-intensity statin plus ezetimibe vs high-intensity statin according to baseline LDL-C in the treatment of atherosclerotic cardiovascular disease: A post-hoc analysis of the RACING randomized trial. | remove |
| **426** | Medium-intensity statin with ezetimibe versus high-intensity statin in acute ischemic cerebrovascular disease (MESIA): A randomized clinical trial. | remove |
| **427** | Effect of evolocumab or ezetimibe added to moderate- or high-intensity statin therapy on LDL-C lowering in patients with hypercholesterolemia: the LAPLACE-2 randomized clinical trial. | remove |
| **428** | Effect of rosuvastatin 20 mg versus rosuvastatin 5 mg plus ezetimibe on statin side-effects in elderly patients with atherosclerotic cardiovascular disease: Rationale and design of a randomized, controlled SaveSAMS trial. | remove |
| **429** | Effects of alirocumab on cardiovascular and metabolic outcomes after acute coronary syndrome in patients with or without diabetes: a prespecified analysis of the ODYSSEY OUTCOMES randomised controlled trial. | remove |
| **430** | Lipid-Modifying Efficacy and Tolerability of Anacetrapib Added to Ongoing Statin Therapy in Patients with Hypercholesterolemia or Low High-Density Lipoprotein Cholesterol. | remove |
| **431** | A randomized, controlled comparison of different intensive lipid-lowering therapies in Chinese patients with non-ST-elevation acute coronary syndrome (NSTE-ACS): Ezetimibe and rosuvastatin versus high-dose rosuvastatin. | remove |
| **432** | Factors Associated With Enhanced Low-Density Lipoprotein Cholesterol Lowering With Bempedoic Acid. | remove |
| **433** | Effect of Atorvastatin (10 mg) and Ezetimibe (10 mg) Combination Compared to Atorvastatin (40 mg) Alone on Coronary Atherosclerosis. | remove |
| **434** | Ezetimibe and Rosuvastatin Combination Treatment Can Reduce the Dose of Rosuvastatin Without Compromising Its Lipid-lowering Efficacy. | remove |
| **435** | Efficacy and safety of gemcabene as add-on to stable statin therapy in hypercholesterolemic patients. | remove |
| **436** | Pragmatic Trial of Messaging to Providers About Treatment of Hyperlipidemia (PROMPT-LIPID): A Randomized Clinical Trial. | remove |
| **437** | Pharmacological Inhibition of CETP (Cholesteryl Ester Transfer Protein) Increases HDL (High-Density Lipoprotein) That Contains ApoC3 and Other HDL Subspecies Associated With Higher Risk of Coronary Heart Disease. | remove |
| **438** | Randomized Trial of a Vascular Care Team vs Education for Patients With Peripheral Artery Disease. | remove |
| **439** | Cost-effectiveness of Simvastatin plus Ezetimibe for Cardiovascular Prevention in CKD: Results of the Study of Heart and Renal Protection (SHARP). | remove |
| **440** | Additive effects of plant sterols supplementation in addition to different lipid-lowering regimens. | remove |
| **441** | Efficacy and Safety of Alirocumab in Adults With Homozygous Familial Hypercholesterolemia: The ODYSSEY HoFH Trial. | remove |
| **442** | Open-label therapy with alirocumab in patients with heterozygous familial hypercholesterolemia: Results from three years of treatment. | remove |
| **443** | Safety and efficacy of ezetimibe added on to rosuvastatin 5 or 10 mg versus up-titration of rosuvastatin in patients with hypercholesterolemia (the ACTE Study). | remove |
| **444** | Factors Associated With Enhanced Low-Density Lipoprotein Cholesterol Lowering With Bempedoic Acid. | remove |
| **445** | Ezetimibe/simvastatin vs atorvastatin in patients with type 2 diabetes mellitus and hypercholesterolemia: the VYTAL study. | remove |
| **446** | The efficacy of statin monotherapy uptitration versus switching to ezetimibe/simvastatin: results of the EASEGO study. | remove |
| **447** | The effects of ezetimibe/simvastatin versus simvastatin monotherapy on platelet and inflammatory biomarkers in patients with metabolic syndrome. | remove |
| **448** | Efficacy and safety of ezetimibe co-administered with simvastatin in thiazolidinedione-treated type 2 diabetic patients. | remove |
| **449** | Effects of fluvastatin extended-release (80 mg) alone and in combination with ezetimibe (10 mg) on low-density lipoprotein cholesterol and inflammatory parameters in patients with primary hypercholesterolemia: a 12-week, multicenter, randomized, open-label, parallel-group study. | remove |
| **450** | Effects of combined ezetimibe and simvastatin therapy as compared with simvastatin alone in patients with type 2 diabetes: a prospective randomized double-blind clinical trial. | remove |
| **451** | Virtual histology evaluation of atherosclerosis regression during atorvastatin and ezetimibe administration: HEAVEN study. | remove |
| **452** | Efficacy of combination of Ezetimibe 10 mg and rosuvastatin 2.5 mg versus rosuvastatin 5 mg monotherapy for hypercholesterolemia in patients with type 2 diabetes. | remove |
| **453** | Effect of combination therapy of ezetimibe and rosuvastatin on regression of coronary atherosclerosis in patients with coronary artery disease. | remove |
| **454** | A comparison of efficacy and safety of an ezetimibe/simvastatin combination compared with other intensified lipid-lowering treatment strategies in diabetic patients with symptomatic cardiovascular disease. | remove |
| **455** | Comparison of PCSK9 Inhibitor Evolocumab vs Ezetimibe in Statin-Intolerant Patients: Design of the Goal Achievement After Utilizing an Anti-PCSK9 Antibody in Statin-Intolerant Subjects 3 (GAUSS-3) Trial. | remove |
| **456** | Long-term efficacy and safety of ezetimibe/simvastatin coadministered with extended-release niacin in hyperlipidaemic patients with diabetes or metabolic syndrome. | remove |
| **457** | Safety and efficacy of ezetimibe added to atorvastatin versus up titration of atorvastatin to 40 mg in Patients > or = 65 years of age (from the ZETia in the ELDerly [ZETELD] study). | remove |
| **458** | Clinical usefulness of additional treatment with ezetimibe in patients with coronary artery disease on statin therapy. - From the viewpoint of cholesterol metabolism.-. | remove |
| **459** | Lipid-altering efficacy and safety of ezetimibe/simvastatin coadministered with extended-release niacin in patients with type IIa or type IIb hyperlipidemia. | remove |
| **460** | Pleiotropic effects with equivalent low-density lipoprotein cholesterol reduction: comparative study between simvastatin and simvastatin/ezetimibe coadministration. | remove |
| **461** | Ezetimibe combined with simvastatin compared with simvastatin alone results in a greater suppression of oxidative stress and enhanced fibrinolysis in patients after acute coronary events. | remove |
| **462** | Consistency of effect of ezetimibe/simvastatin compared with intensified lipid-lowering treatment strategies in obese and non-obese diabetic subjects. | remove |
| **463** | Impact of QRS duration and morphology on the risk of sudden cardiac death in asymptomatic patients with aortic stenosis: the SEAS (Simvastatin and Ezetimibe in Aortic Stenosis) Study. | remove |
| **464** | Clinical implications of electrocardiographic left ventricular strain and hypertrophy in asymptomatic patients with aortic stenosis: the Simvastatin and Ezetimibe in Aortic Stenosis study. | remove |
| **465** | A Phase 3 Randomized Controlled Trial to Evaluate Efficacy and Safety of New-Formulation Zenon (Rosuvastatin/Ezetimibe Fixed-Dose Combination) in Primary Hypercholesterolemia Inadequately Controlled by Statins. | remove |
| **466** | Comparison of the effect of simvastatin versus simvastatin/ezetimibe versus rosuvastatin on markers of inflammation and oxidative stress in subjects with hypercholesterolemia. | remove |
| **467** | Efficacy and safety of ezetimibe co-administered with atorvastatin in untreated patients with primary hypercholesterolaemia and coronary heart disease. | remove |
| **468** | Medication Discontinuation in the IMPROVE-IT Trial. | remove |
| **469** | Lipid-altering efficacy of ezetimibe/simvastatin 10/40 mg compared with doubling the statin dose in patients admitted to the hospital for a recent coronary event: the INFORCE study. | remove |
| **470** | Effects of ezetimibe on plasma lipoproteins in severely hypercholesterolemic patients treated with regular LDL-apheresis and statins. | remove |
| **471** | A Bioequivalence Study of Ezetimibe/Rosuvastatin Fixed Dose Combination (10 mg/10 mg) Versus the Individual Formulations Taken Concomitantly. | remove |
| **472** | [Efficacy and safety of alirocumab versus ezetimibe in high cardiovascular risk Chinese patients with hyperlipidemia: ODYSSEY EAST Study-Chinese sub-population analysis]. | remove |
| **473** | A randomized, controlled comparison of different intensive lipid-lowering therapies in Chinese patients with non-ST-elevation acute coronary syndrome (NSTE-ACS): Ezetimibe and rosuvastatin versus high-dose rosuvastatin. | remove |
| **474** | Paediatric patients with heterozygous familial hypercholesterolaemia treated with evolocumab for 80 weeks (HAUSER-OLE): a single-arm, multicentre, open-label extension of HAUSER-RCT. | remove |
| **475** | Effect of coadministration of ezetimibe and simvastatin on high-sensitivity C-reactive protein. | remove |
| **476** | Efficacy and safety of coadministration of ezetimibe and simvastatin in African-American patients with primary hypercholesterolemia. | remove |
| **477** | Comparative efficacy and safety of fenofibrate/pravastatin plus ezetimibe triple therapy and simvastatin/ezetimibe dual therapy in type 2 diabetic patients with mixed hyperlipidaemia and cardiovascular disease. | remove |
| **478** | Baseline cholesterol absorption and the response to ezetimibe/simvastatin therapy: a post-hoc analysis of the ENHANCE trial. | remove |
| **479** | Effects of ezetimibe added to on-going statin therapy on the lipid profile of hypercholesterolemic patients with diabetes mellitus or metabolic syndrome. | remove |
| **480** | Effects of Extended-Release Niacin on Quartile Lp-PLA(2) Levels and Clinical Outcomes in Statin-treated Patients with Established Cardiovascular Disease and Low Baseline Levels of HDL-Cholesterol: Post Hoc Analysis of the AIM HIGH Trial. | remove |
| **481** | Dose-comparison study of the combination of ezetimibe and simvastatin (Vytorin) versus atorvastatin in patients with hypercholesterolemia: the Vytorin Versus Atorvastatin (VYVA) study. | remove |
| **482** | Left atrial size and function as predictors of new-onset of atrial fibrillation in patients with asymptomatic aortic stenosis: the simvastatin and ezetimibe in aortic stenosis study. | remove |
| **483** | Endothelial Effect of Statin Therapy at a High Dose Versus Low Dose Associated with Ezetimibe. | remove |
| **484** | Efficacy and safety of rosuvastatin 40 mg alone or in combination with ezetimibe in patients at high risk of cardiovascular disease (results from the EXPLORER study). | remove |
| **485** | Comparative study between high-dose fluvastatin and low-dose fluvastatin and ezetimibe with regard to the effect on endothelial function in diabetic patients. | remove |
| **486** | Long-term safety and, tolerability profiles and lipid-modifying efficacy of ezetimibe coadministered with ongoing simvastatin treatment: a multicenter, randomized, double-blind, placebo-controlled, 48-week extension study. | remove |
| **487** | Effects of atorvastatin 20 mg, rosuvastatin 10 mg, and atorvastatin/ezetimibe 5 mg/5 mg on lipoproteins and glucose metabolism. | remove |
| **488** | Therapeutic effects of atorvastatin and ezetimibe compared with double-dose atorvastatin in very elderly patients with acute coronary syndrome. | remove |
| **489** | Simvastatin with or without ezetimibe in familial hypercholesterolemia. | remove |
| **490** | Double-dose pravastatin versus add-on ezetimibe with low-dose pravastatin - effects on LDL cholesterol, cholesterol absorption, and cholesterol synthesis in Japanese patients with hypercholesterolemia (PEAS study). | remove |
| **491** | Hypolipidaemic and anti-inflammatory effects of fixed dose combination of atorvastatin plus ezetimibe in Indian patients with dyslipidaemia. | remove |
| **492** | [Comparative Randomized Study of the Effects of Long-Term Therapy With Rosuvastatin and Combination of Atorvastatin and Ezetimibe on Carbohydrate Metabolism and Adipokines Levels in Patients With Coronary Artery Disease and Diabetes Mellitus]. | remove |
| **493** | VAP II analysis of lipoprotein subclasses in mixed hyperlipidemic patients on treatment with ezetimibe/simvastatin and fenofibrate. | remove |
| **494** | Effect of a monoclonal antibody to PCSK9, REGN727/SAR236553, to reduce low-density lipoprotein cholesterol in patients with heterozygous familial hypercholesterolaemia on stable statin dose with or without ezetimibe therapy: a phase 2 randomised controlled trial. | remove |
| **495** | The second United Kingdom Heart and Renal Protection (UK-HARP-II) Study: a randomized controlled study of the biochemical safety and efficacy of adding ezetimibe to simvastatin as initial therapy among patients with CKD. | remove |
| **496** | Ezetimibe Increases Endogenous Cholesterol Excretion in Humans. | remove |
| **497** | Clinical Profile of Statin Intolerance in the Phase 3 GAUSS-2 Study. | remove |
| **498** | Influence of age, gender, and race on the efficacy of adding ezetimibe to atorvastatin vs. atorvastatin up-titration in patients at moderately high or high risk for coronary heart disease. | remove |
| **499** | Effect of simvastatin or its combination with ezetimibe on Toll-like receptor expression and lipopolysaccharide - induced cytokine production in monocytes of hypercholesterolemic patients. | remove |
| **500** | Efficacy and safety of the coadministration of ezetimibe/simvastatin with fenofibrate in patients with mixed hyperlipidemia. | remove |
| **501** | Cost-effectiveness of Simvastatin plus Ezetimibe for Cardiovascular Prevention in CKD: Results of the Study of Heart and Renal Protection (SHARP). | remove |
| **502** | Long-term safety and efficacy of triple combination ezetimibe/simvastatin plus extended-release niacin in patients with hyperlipidemia. | remove |
| **503** | Simvastatin but not ezetimibe reduces sympathetic activity despite similar reductions in cholesterol levels. | remove |
| **504** | Efficacy and safety of ezetimibe co-administered with ongoing atorvastatin therapy in achieving low-density lipoprotein goal in patients with hypercholesterolemia and coronary heart disease. | remove |
| **505** | The effects of low-dose simvastatin and ezetimibe compared to high-dose simvastatin alone on post-fat load endothelial function in patients with metabolic syndrome: a randomized double-blind crossover trial. | remove |
| **506** | Changes in lipoproteins associated with lipid-lowering and antiplatelet strategies in patients with acute myocardial infarction. | remove |
| **507** | Polyvascular disease, type 2 diabetes, and long-term vascular risk: a secondary analysis of the IMPROVE-IT trial. | remove |
| **508** | Polyvascular disease, type 2 diabetes, and long-term vascular risk: a secondary analysis of the IMPROVE-IT trial. | remove |
| **509** | Lipid-Modifying Efficacy and Tolerability of Anacetrapib Added to Ongoing Statin Therapy in Patients with Hypercholesterolemia or Low High-Density Lipoprotein Cholesterol. | remove |
| **510** | Effects of ezetimibe/simvastatin 10/10 mg versus Rosuvastatin 10 mg on carotid atherosclerotic plaque inflammation. | remove |
| **511** | Prognostic importance of atrial fibrillation in asymptomatic aortic stenosis: the Simvastatin and Ezetimibe in Aortic Stenosis study. | remove |
| **512** | Effect of lipid lowering on new-onset atrial fibrillation in patients with asymptomatic aortic stenosis: the Simvastatin and Ezetimibe in Aortic Stenosis (SEAS) study. | remove |
| **513** | Incremental cholesterol reduction with ezetimibe/simvastatin, atorvastatin and rosuvastatin in UK General Practice (IN-PRACTICE): randomised controlled trial of achievement of Joint British Societies (JBS-2) cholesterol targets. | remove |
| **514** | Atorvastatin 10 mg plus ezetimibe 10mg compared with atorvastatin 20 mg: impact on the lipid profile in Japanese patients with abnormal glucose tolerance and coronary artery disease. | remove |
| **515** | Efficacy and Safety of Alirocumab 150 mg Every 4 Weeks in Patients With Hypercholesterolemia Not on Statin Therapy: The ODYSSEY CHOICE II Study. | remove |
| **516** | The effect of ezetimibe, administered alone or in combination with simvastatin, on lymphocyte cytokine release in patients with elevated cholesterol levels. | remove |
| **517** | Effect Modifications of Lipid-Lowering Therapy on Progression of Aortic Stenosis (from the Simvastatin and Ezetimibe in Aortic Stenosis [SEAS] Study). | remove |
| **518** | A randomised trial of three counselling strategies for lifestyle changes in patients with hypercholesterolemia treated with ezetimibe on top of statin therapy (TWICE). | remove |
| **519** | The efficacy of colesevelam HCl in the treatment of heterozygous familial hypercholesterolemia in pediatric and adult patients. | remove |
| **520** | Effect of Ezetimibe + Pitavastatin on Cardiovascular Outcomes in Patients with ST-Segment Elevation Myocardial Infarction (from the HIJ-PROPER Study). | remove |
| **521** | Influence of Ezetimibe on Plaque Morphology in Patients with ST Elevation Myocardial Infarction Assessed by Optical Coherence Tomography: An OCTIVUS Sub-Study. | remove |
| **522** | Achieving lipoprotein goals in patients at high risk with severe hypercholesterolemia: efficacy and safety of ezetimibe co-administered with atorvastatin. | remove |
| **523** | Atorvastatin 10 mg plus ezetimibe versus titration to atorvastatin 40 mg: attainment of European and Canadian guideline lipid targets in high-risk subjects 65 years. | remove |
| **524** | Effect of Simvastatin-Ezetimibe Compared With Simvastatin Monotherapy After Acute Coronary Syndrome Among Patients 75 Years or Older: A Secondary Analysis of a Randomized Clinical Trial. | remove |
| **525** | Triglyceride concentrations and non-high-density lipoprotein cholesterol goal attainment in the ODYSSEY phase 3 trials with alirocumab. | remove |
| **526** | Efficacy and safety of alirocumab in high cardiovascular risk patients with inadequately controlled hypercholesterolaemia on maximally tolerated doses of statins: the ODYSSEY COMBO II randomized controlled trial. | remove |
| **527** | Ezetimibe in Combination With Simvastatin Reduces Remnant Cholesterol Without Affecting Biliary Lipid Concentrations in Gallstone Patients. | remove |
| **528** | Efficacy of Rosuvastatin in Children With Homozygous Familial Hypercholesterolemia and Association With Underlying Genetic Mutations. | remove |
| **529** | The VYtorin on Carotid intima-media thickness and overall arterial rigidity (VYCTOR) study. | remove |
| **530** | Endothelial progenitor cell levels in obese men with the metabolic syndrome and the effect of simvastatin monotherapy vs. simvastatin/ezetimibe combination therapy. | remove |
| **531** | Ezetimibe coadministered with simvastatin in patients with primary hypercholesterolemia. | remove |
| **532** | Effect of intensive lipid-lowering therapies on cholinesterase activity in patients with coronary artery disease. | remove |
| **533** | Alirocumab vs usual lipid-lowering care as add-on to statin therapy in individuals with type 2 diabetes and mixed dyslipidaemia: The ODYSSEY DM-DYSLIPIDEMIA randomized trial. | remove |
| **534** | Usefulness of Gemcabene in Homozygous Familial Hypercholesterolemia (from COBALT-1). | remove |
| **535** | Ezetimibe effectively reduces plasma plant sterols in patients with sitosterolemia. | remove |
| **536** | Reductions in Atherogenic Lipids and Major Cardiovascular Events: A Pooled Analysis of 10 ODYSSEY Trials Comparing Alirocumab With Control. | remove |
| **537** | Low myocardial energetic efficiency is associated with increased mortality in aortic stenosis. | remove |
| **538** | Efficacy of ezetimibe/simvastatin 10/20 and 10/40 mg compared with atorvastatin 20 mg in patients with type 2 diabetes mellitus. | remove |
| **539** | Lipid-altering efficacy of the ezetimibe/simvastatin single tablet versus rosuvastatin in hypercholesterolemic patients. | remove |
| **540** | Effect of evolocumab on cholesterol synthesis and absorption. | remove |
| **541** | Lipid-altering efficacy of switching to ezetimibe/simvastatin 10/20 mg versus rosuvastatin 10 mg in high-risk patients with and without metabolic syndrome. | remove |
| **542** | Usefulness of the electrocardiogram in predicting cardiovascular mortality in asymptomatic adults with aortic stenosis (from the Simvastatin and Ezetimibe in Aortic Stenosis Study). | remove |
| **543** | Evidence from a randomized trial that simvastatin, but not ezetimibe, upregulates circulating PCSK9 levels. | remove |
| **544** | The nonalcoholic fatty liver disease (NAFLD) fibrosis score, cardiovascular risk stratification and a strategy for secondary prevention with ezetimibe. | remove |
| **545** | Higher Acceleration/Ejection Time Ratio Predicts Impaired Outcome in Aortic Valve Stenosis. | remove |
| **546** | Effects of Combined Lipid-Lowering Therapy on Low-Density Lipoprotein Cholesterol Variability and Cardiovascular Adverse Events in Patients with Acute Coronary Syndrome. | remove |
| **547** | Patient and physician factors influence decision-making in hypercholesterolemia: a questionnaire-based survey. | remove |
| **548** | [ACUTE CORONARY SYNDROME AND LIPID-LOWERING THERAPY. DOES THE IMPROVE-IT STUDY MAKE ANY DIFFERENCE?]. | remove |
| **549** | Nutraceutical pill containing berberine versus ezetimibe on plasma lipid pattern in hypercholesterolemic subjects and its additive effect in patients with familial hypercholesterolemia on stable cholesterol-lowering treatment. | remove |
| **550** | Benefit of Targeting a LDL (Low-Density Lipoprotein) Cholesterol <70 mg/dL During 5 Years After Ischemic Stroke. | remove |
| **551** | Benefit of Targeting a LDL (Low-Density Lipoprotein) Cholesterol <70 mg/dL During 5 Years After Ischemic Stroke. | remove |
| **552** | Differences in synthesis and absorption of cholesterol of two effective lipid-lowering therapies. | remove |
| **553** | Fixed-dose combination ezetimibe+atorvastatin lowers LDL-C equivalent to co-administered components in randomized trials: use of a dose-response model. | remove |
| **554** | Improved endothelial function with simvastatin but unchanged insulin sensitivity with simvastatin or ezetimibe. | remove |
| **555** | Alirocumab efficacy and safety by race and ethnicity: Analysis from 3 ODYSSEY phase 3 trials. | remove |
| **556** | Treatment of alopecia areata with simvastatin/ezetimibe. | remove |
| **557** | Changes in lipoprotein lipase and endothelial lipase mass in familial hypercholesterolemia during three-drug lipid-lowering combination therapy. | remove |
| **558** | Lipid-altering efficacy of ezetimibe/simvastatin 10/20 mg compared with rosuvastatin 10 mg in high-risk hypercholesterolaemic patients inadequately controlled with prior statin monotherapy - The IN-CROSS study. | remove |
| **559** | Lipid-lowering treatment and inflammatory mediators in diabetes and chronic kidney disease. | remove |
| **560** | Efficacy, safety, and tolerability of a monoclonal antibody to proprotein convertase subtilisin/kexin type 9 in combination with a statin in patients with hypercholesterolaemia (LAPLACE-TIMI 57): a randomised, placebo-controlled, dose-ranging, phase 2 study. | remove |
| **561** | Ezetimibe/simvastatin 10/20 mg versus rosuvastatin 10 mg in high-risk hypercholesterolemic patients stratified by prior statin treatment potency. | remove |
| **562** | Effect of therapeutic interventions on oxidized phospholipids on apolipoprotein B100 and lipoprotein(a). | remove |
| **563** | Ezetimibe alone reduces low-density lipoprotein cholesterol in HIV-infected patients receiving combination antiretroviral therapy. | remove |
| **564** | Variability and persistence of aspirin response in lower extremity peripheral arterial disease patients. | remove |
| **565** | Treatment with ezetimibe plus low-dose atorvastatin compared with higher-dose atorvastatin alone: is sufficient cholesterol-lowering enough to inhibit platelets? | remove |
| **566** | Effect of ezetimibe on low-density lipoprotein subtype distribution: results of a placebo-controlled, double-blind trial in patients treated by regular low-density lipoprotein apheresis and statins. | remove |
| **567** | Flow-mediated dilation in patients with coronary artery disease is enhanced by high dose atorvastatin compared to combined low dose atorvastatin and ezetimibe: results of the CEZAR study. | remove |
| **568** | Benefit of Ezetimibe Added to Simvastatin in Reduced Kidney Function. | remove |
| **569** | Ezetimibe in Combination With Statins Ameliorates Endothelial Dysfunction in Coronary Arteries After Stenting: The CuVIC Trial (Effect of Cholesterol Absorption Inhibitor Usage on Target Vessel Dysfunction After Coronary Stenting), a Multicenter Randomized Controlled Trial. | remove |
| **570** | Pharmacological Inhibition of CETP (Cholesteryl Ester Transfer Protein) Increases HDL (High-Density Lipoprotein) That Contains ApoC3 and Other HDL Subspecies Associated With Higher Risk of Coronary Heart Disease. | remove |
| **571** | Lower Transaortic Flow Rate Is Associated With Increased Mortality in Aortic Valve Stenosis. | remove |
| **572** | Effect of ezetimibe coadministration with simvastatin in a Middle Eastern population: a prospective, multicentre, randomized, double-blind, placebo-controlled trial. | remove |
| **573** | Effect of ezetimibe coadministration with simvastatin in a Middle Eastern population: a prospective, multicentre, randomized, double-blind, placebo-controlled trial. | remove |
| **574** | Lower Transaortic Flow Rate Is Associated With Increased Mortality in Aortic Valve Stenosis. | remove |
| **575** | Effect of ezetimibe coadministration with simvastatin in a Middle Eastern population: a prospective, multicentre, randomized, double-blind, placebo-controlled trial. | remove |
| **576** | Effect of a monoclonal antibody to PCSK9 on low-density lipoprotein cholesterol levels in statin-intolerant patients: the GAUSS randomized trial. | remove |
| **577** | Effect of a monoclonal antibody to PCSK9 on low-density lipoprotein cholesterol levels in statin-intolerant patients: the GAUSS randomized trial. | remove |
| **578** | Low-density lipoprotein cholesterol reduction and goal achievement with ezetimibe/simvastatin versus atorvastatin or rosuvastatin in patients with diabetes, metabolic syndrome, or neither disease, stratified by National Cholesterol Education Program risk category. | remove |
| **579** | Evaluation of two highly effective lipid-lowering therapies in subjects with acute myocardial infarction. | remove |
| **580** | The Effect of Lipid Modification on Peripheral Artery Disease after Endovascular Intervention Trial (ELIMIT). | remove |
| **581** | Polyunsaturated Fatty Acid Impact on Clinical Outcomes in Acute Coronary Syndrome Patients With Dyslipidemia: Subanalysis of HIJ-PROPER. | remove |
| **582** | The short-term effect of atorvastatin plus ezetimibe therapy versus atorvastatin monotherapy on clinical outcome in acute coronary syndrome patients by gender. | remove |
| **583** | Short-term treatment with ezetimibe, simvastatin or their combination does not alter circulating adiponectin, resistin or leptin levels in healthy men. | remove |
| **584** | The short-term effect of atorvastatin plus ezetimibe therapy versus atorvastatin monotherapy on clinical outcome in acute coronary syndrome patients by gender. | remove |
| **585** | Lipid-lowering efficacy and safety of alirocumab in patients with or without diabetes: A sub-analysis of ODYSSEY COMBO II. | remove |
| **586** | Ezetimibe alone or in combination with simvastatin increases small dense low-density lipoproteins in healthy men: a randomized trial. | remove |
| **587** | Comparison of High-Dose Rosuvastatin Versus Low-Dose Rosuvastatin Plus Ezetimibe on Carotid Atherosclerotic Plaque Inflammation in Patients with Acute Coronary Syndrome. | remove |
| **588** | Efficacy of ezetimibe combined with atorvastatin in the treatment of carotid artery plaque in patients with type 2 diabetes mellitus complicated with coronary heart disease. | remove |
| **589** | Lower On-Treatment Low-Density Lipoprotein Cholesterol and Major Adverse Cardiovascular Events in Women and Men: Pooled Analysis of 10 ODYSSEY Phase 3 Alirocumab Trials. | remove |
| **590** | Relationship Between Low-Density Lipoprotein Cholesterol, Free Proprotein Convertase Subtilisin/Kexin Type 9, and Alirocumab Levels After Different Lipid-Lowering Strategies. | remove |
| **591** | A multi-centre, randomised, double-blind 14-week extension study examining the long-term safety and efficacy profile of the ezetimibe/simvastatin combination tablet. | remove |
| **592** | Additive effects of plant sterols supplementation in addition to different lipid-lowering regimens. | remove |
| **593** | Rationale and design of REDUCE-IT: Reduction of Cardiovascular Events with Icosapent Ethyl-Intervention Trial. | remove |
| **594** | Rationale and design of REDUCE-IT: Reduction of Cardiovascular Events with Icosapent Ethyl-Intervention Trial. | remove |
| **595** | Effect of Nutrition Education in NAFLD Patients Undergoing Simultaneous Hyperlipidemia Pharmacotherapy: A Randomized Controlled Trial. | remove |
| **596** | Relations of GlycA and lipoprotein particle subspecies with cardiovascular events and mortality: A post hoc analysis of the AIM-HIGH trial. | remove |
| **597** | Favorable effects of ezetimibe alone or in association with simvastatin on the removal from plasma of chylomicrons in coronary heart disease subjects. | remove |
| **598** | Echocardiographic aortic valve calcification and outcomes in women and men with aortic stenosis. | remove |
| **599** | Effects of ezetimibe coadministered with simvastatin on C-reactive protein in a large cohort of hypercholesterolemic patients. | remove |
| **600** | Effect of statins on skeletal muscle function. | remove |
| **601** | Lipid-lowering effects of colesevelam HCl in combination with ezetimibe. | remove |
| **602** | Cost-Effectiveness of Simvastatin Plus Ezetimibe for Cardiovascular Prevention in Patients With a History of Acute Coronary Syndrome: Analysis of Results of the IMPROVE-IT Trial. | remove |
| **603** | Treatment of high-risk patients with ezetimibe plus simvastatin co-administration versus simvastatin alone to attain National Cholesterol Education Program Adult Treatment Panel III low-density lipoprotein cholesterol goals. | remove |
| **604** | Resting heart rate and risk of adverse cardiovascular outcomes in asymptomatic aortic stenosis: the SEAS study. | remove |
| **605** | Influence of intensive lipid-lowering on CT derived fractional flow reserve in patients with stable chest pain: Rationale and design of the FLOWPROMOTE study. | remove |
| **606** | Carotid Atherosclerosis Evolution When Targeting a Low-Density Lipoprotein Cholesterol Concentration <70 mg/dL After an Ischemic Stroke of Atherosclerotic Origin. | remove |
| **607** | PCSK9 inhibition in patients with and without prior myocardial infarction or ischemic stroke: A pooled analysis of nine randomized-controlled studies of alirocumab. | remove |
| **608** | Impact on optical coherence tomographic coronary findings of fluvastatin alone versus fluvastatin + ezetimibe. | remove |
| **609** | Effect of ezetimibe/atorvastatin combination on oxidized low density lipoprotein cholesterol in patients with coronary artery disease or coronary artery disease equivalent. | remove |
| **610** | Rationale and design of IMPROVE-IT (IMProved Reduction of Outcomes: Vytorin Efficacy International Trial): comparison of ezetimbe/simvastatin versus simvastatin monotherapy on cardiovascular outcomes in patients with acute coronary syndromes. | remove |
| **611** | Differential effects of fluvastatin alone or in combination with ezetimibe on lipoprotein subfractions in patients at high risk of coronary events. | remove |
| **612** | Critical appraisal of the SHARP trial: the results may be dull. | remove |
| **613** | Ezetimibe/simvastatin vs simvastatin in coronary heart disease patients with or without diabetes. | remove |
| **614** | A pilot study of ezetimibe vs. atorvastatin for improving peripheral microvascular endothelial function in stable patients with type 2 diabetes mellitus. | remove |
| **615** | Small aortic root in aortic valve stenosis: clinical characteristics and prognostic implications. | remove |
| **616** | Can group medical clinics improve lipid management in diabetes? | remove |
| **617** | Hypertension in aortic stenosis: implications for left ventricular structure and cardiovascular events. | remove |
| **618** | Pharmacokinetics and exploratory efficacy biomarkers of bococizumab, an anti-PCSK9 monoclonal antibody, in hypercholesterolemic Japanese subjects. | remove |
| **619** | The effect of simvastatin alone versus simvastatin plus ezetimibe on the concentration of small dense low-density lipoprotein cholesterol in subjects with primary hypercholesterolemia. | remove |
| **620** | Effects of ezetimibe/simvastatin 10/20 mg vs. atorvastatin 20 mg on apolipoprotein B/apolipoprotein A1 in Korean patients with type 2 diabetes mellitus: results of a randomized controlled trial. | remove |
| **621** | Low-density lipoprotein cholesterol treatment and outcomes in patients with type 2 diabetes and established cardiovascular disease: Insights from TECOS. | remove |
| **622** | Lipid lowering with bempedoic acid added to a proprotein convertase subtilisin/kexin type 9 inhibitor therapy: A randomized, controlled trial. | remove |
| **623** | Monotherapy with the PCSK9 inhibitor alirocumab versus ezetimibe in patients with hypercholesterolemia: results of a 24 week, double-blind, randomized Phase 3 trial. | remove |
| **624** | Impact of Educational Attainment on Health Outcomes in Moderate to Severe CKD. | remove |
| **625** | Reduced gut microbial diversity in familial hypercholesterolemia with no effect of omega-3 polyunsaturated fatty acids intervention - a pilot trial. | remove |
| **626** | Impact of baseline severity of aortic valve stenosis on effect of intensive lipid lowering therapy (from the SEAS study). | remove |
| **627** | Efficacy and safety of alirocumab, a fully human PCSK9 monoclonal antibody, in high cardiovascular risk patients with poorly controlled hypercholesterolemia on maximally tolerated doses of statins: rationale and design of the ODYSSEY COMBO I and II trials. | remove |
| **628** | Impact of baseline severity of aortic valve stenosis on effect of intensive lipid lowering therapy (from the SEAS study). | remove |
| **629** | Effects of two lipid lowering therapies on immune responses in hyperlipidemic subjects. | remove |
| **630** | Co-administration of ezetimibe enhances proteinuria-lowering effects of pitavastatin in chronic kidney disease patients partly via a cholesterol-independent manner. | remove |
| **631** | Anti-PCSK9 antibody effectively lowers cholesterol in patients with statin intolerance: the GAUSS-2 randomized, placebo-controlled phase 3 clinical trial of evolocumab. | remove |
| **632** | A Randomized Open-Label Trial to Assess the Effect of Plant Sterols Associated with Ezetimibe in Low-Density Lipoprotein Levels in Patients with Coronary Artery Disease on Statin Therapy. | remove |
| **633** | The effects of high dose pravastatin and low dose pravastatin and ezetimibe combination therapy on lipid, glucose metabolism and inflammation. | remove |
| **634** | Patient Phenotypes, Cardiovascular Risk, and Ezetimibe Treatment in Patients After Acute Coronary Syndromes (from IMPROVE-IT). | remove |
| **635** | Effect of Combination Therapy of Ezetimibe and Atorvastatin on Remnant Lipoprotein Versus Double Atorvastatin Dose in Egyptian Diabetic Patients. | remove |
| **636** | Intensive lipid lowering with simvastatin and ezetimibe in aortic stenosis. | remove |
| **637** | Pharmacodynamic interaction between the new selective cholesterol absorption inhibitor ezetimibe and simvastatin. | remove |
| **638** | Efficacy and safety of ezetimibe added to atorvastatin versus atorvastatin uptitration or switching to rosuvastatin in patients with primary hypercholesterolemia. | remove |
| **639** | Effect of ezetimibe/simvastatin compared with atorvastatin on lipoprotein subclasses in patients with type 2 diabetes and hypercholesterolaemia. | remove |
| **640** | A multicenter, randomized, double-blind, placebo-controlled, factorial design study to evaluate the lipid-altering efficacy and safety profile of the ezetimibe/simvastatin tablet compared with ezetimibe and simvastatin monotherapy in patients with primary hypercholesterolemia. | remove |
| **641** | Efficacy, safety and LDL-C goal attainment of ezetimibe 10 mg-simvastatin 20 mg vs. placebo-simvastatin 20 mg in UK-based adults with coronary heart disease and hypercholesterolaemia. | remove |
| **642** | Long-term efficacy and safety of ezetimibe 10 mg in patients with homozygous sitosterolemia: a 2-year, open-label extension study. | remove |
| **643** | Effects of Extended-Release Niacin Added to Simvastatin/Ezetimibe on Glucose and Insulin Values in AIM-HIGH. | remove |
| **644** | Colesevelam HCl and ezetimibe combination therapy provides effective lipid-lowering in difficult-to-treat patients with hypercholesterolemia. | remove |
| **645** | Low-density lipoprotein cholesterol targeting with pitavastatin + ezetimibe for patients with acute coronary syndrome and dyslipidaemia: the HIJ-PROPER study, a prospective, open-label, randomized trial. | remove |
| **646** | Simvastatin versus ezetimibe: pleiotropic and lipid-lowering effects on endothelial function in humans. | remove |
| **647** | Long-term safety and tolerability profile of ezetimibe and atorvastatin coadministration therapy in patients with primary hypercholesterolaemia. | remove |
| **648** | Prevention of Stroke with the Addition of Ezetimibe to Statin Therapy in Patients With Acute Coronary Syndrome in IMPROVE-IT (Improved Reduction of Outcomes: Vytorin Efficacy International Trial). | remove |
| **649** | Detrimental effects of high-fat diet loading on vascular endothelial function and therapeutic efficacy of ezetimibe and statins in patients with type 2 diabetes. | remove |
| **650** | Efficacy and safety of adding alirocumab to rosuvastatin versus adding ezetimibe or doubling the rosuvastatin dose in high cardiovascular-risk patients: The ODYSSEY OPTIONS II randomized trial. | remove |
| **651** | Relationships between metabolic syndrome and other baseline factors and the efficacy of ezetimibe/simvastatin and atorvastatin in patients with type 2 diabetes and hypercholesterolemia. | remove |
| **652** | Effects of ezetimibe and simvastatin on apolipoprotein B metabolism in males with mixed hyperlipidemia. | remove |
| **653** | Co-administration of ezetimibe and simvastatin in acute myocardial infarction. | remove |
| **654** | Efficacy and safety of ezetimibe coadministered with simvastatin in patients with primary hypercholesterolemia: a randomized, double-blind, placebo-controlled trial. | remove |
| **655** | Comparison of anti-inflammatory effects and high-density lipoprotein cholesterol levels between therapy with quadruple-dose rosuvastatin and rosuvastatin combined with ezetimibe. | remove |
| **656** | Synergistic effect of ezetimibe addition on coronary atheroma regression in patients with prior statin therapy: Subanalysis of PRECISE-IVUS trial. | remove |
| **657** | Effects of Ezetimibe/Simvastatin and Rosuvastatin on Oxidative Stress in Diabetic Neuropathy: A Randomized, Double-Blind, Placebo-Controlled Clinical Trial. | remove |
| **658** | Efficacy and safety of ezetimibe coadministered with pravastatin in patients with primary hypercholesterolemia: a prospective, randomized, double-blind trial. | remove |
| **659** | The efficacy and tolerability of ezetimibe in cardiac transplant recipients taking cyclosporin. | remove |
| **660** | A randomized, double-blind, placebo-controlled study of the effect of ezetimibe on glucose metabolism in subjects with type 2 diabetes mellitus and hypercholesterolemia. | remove |
| **661** | Low dose of ROSuvastatin in combination with EZEtimibe effectively and permanently reduce low density lipoprotein cholesterol concentration independently of timing of administration (ROSEZE): A randomized, crossover study - preliminary results. | remove |
| **662** | Paradoxical progression of atherosclerosis related to low-density lipoprotein reduction and exposure to ezetimibe. | remove |
| **663** | Paradoxical progression of atherosclerosis related to low-density lipoprotein reduction and exposure to ezetimibe. | remove |
| **664** | Long-term safety and tolerability of ezetimibe coadministered with simvastatin in hypercholesterolemic patients: a randomized, 12-month double-blind extension study. | remove |
| **665** | Effect of Ezetimibe on LDL-C Lowering and Atherogenic Lipoprotein Profiles in Type 2 Diabetic Patients Poorly Controlled by Statins. | remove |
| **666** | Efficacy and safety of alirocumab in patients with hypercholesterolemia not adequately controlled with non-statin lipid-lowering therapy or the lowest strength of statin: ODYSSEY NIPPON study design and rationale. | remove |
| **667** | The effect of statin alone or in combination with ezetimibe on postprandial lipoprotein composition in obese metabolic syndrome patients. | remove |
| **668** | Efficacy and safety of ezetimibe coadministered with atorvastatin or simvastatin in patients with homozygous familial hypercholesterolemia. | remove |
| **669** | Efficacy and safety of fenofibric acid in combination with atorvastatin and ezetimibe in patients with mixed dyslipidemia. | remove |
| **670** | Effect of Switching From Statin Monotherapy to Ezetimibe/Simvastatin Combination Therapy Compared With Other Intensified Lipid-Lowering Strategies on Lipoprotein Subclasses in Diabetic Patients With Symptomatic Cardiovascular Disease. | remove |
| **671** | Lipid-altering efficacy of ezetimibe/simvastatin 10/20 mg compared to rosuvastatin 10 mg in high-risk patients with and without type 2 diabetes mellitus inadequately controlled despite prior statin monotherapy. | remove |
| **672** | Efficacy and safety of ezetimibe coadministered with statins: randomised, placebo-controlled, blinded experience in 2382 patients with primary hypercholesterolemia. | remove |
| **673** | A community-based, randomized trial of ezetimibe added to statin therapy to attain NCEP ATP III goals for LDL cholesterol in hypercholesterolemic patients: the ezetimibe add-on to statin for effectiveness (EASE) trial. | remove |
| **674** | Assessment of potential pharmacokinetic interactions of ezetimibe/simvastatin and extended-release niacin tablets in healthy subjects. | remove |
| **675** | Effects of Statin Plus Ezetimibe on Coronary Plaques in Acute Coronary Syndrome Patients with Diabetes Mellitus: Sub-Analysis of PRECISE-IVUS Trial. | remove |
| **676** | Efficacy and tolerability of fluvastatin XL 80 mg alone, ezetimibe alone, and the combination of fluvastatin XL 80 mg with ezetimibe in patients with a history of muscle-related side effects with other statins. | remove |
| **677** | The impact of the time of drug administration on the effectiveness of combined treatment of hypercholesterolemia with Rosuvastatin and Ezetimibe (RosEze): study protocol for a randomized controlled trial. | remove |
| **678** | Effects of ezetimibe on markers of synthesis and absorption of cholesterol in high-risk patients with elevated C-reactive protein. | remove |
| **679** | The effect of ezetimibe on peripheral arterial atherosclerosis depends upon statin use at baseline. | remove |
| **680** | Long-term effects of ezetimibe-plus-statin therapy on low-density lipoprotein cholesterol levels as compared with double-dose statin therapy in patients with coronary artery disease. | remove |
| **681** | Efficacy and safety of alirocumab in statin-intolerant patients over 3 years: open-label treatment period of the ODYSSEY ALTERNATIVE trial. | remove |
| **682** | Impact of Ezetimibe on the Rate of Cardiovascular-Related Hospitalizations and Associated Costs Among Patients With a Recent Acute Coronary Syndrome: Results From the IMPROVE-IT Trial (Improved Reduction of Outcomes: Vytorin Efficacy International Trial). | remove |
| **683** | Effect of ezetimibe and/or simvastatin on coenzyme Q10 levels in plasma: a randomised trial. | remove |
| **684** | Lipid-altering efficacy and safety of ezetimibe/simvastatin versus atorvastatin in patients with hypercholesterolemia and the metabolic syndrome (from the VYMET study). | remove |
| **685** | Efficacy and safety of ezetimibe co-administered with simvastatin compared with atorvastatin in adults with hypercholesterolemia. | remove |
| **686** | Efficacy, safety and effect on biomarkers related to cholesterol and lipoprotein metabolism of rosuvastatin 10 or 20 mg plus ezetimibe 10 mg vs. simvastatin 40 or 80 mg plus ezetimibe 10 mg in high-risk patients: Results of the GRAVITY randomized study. | remove |
| **687** | Effects of four antiplatelet/statin combined strategies on immune and inflammatory responses in patients with acute myocardial infarction undergoing pharmacoinvasive strategy: Design and rationale of the B and T Types of Lymphocytes Evaluation in Acute Myocardial Infarction (BATTLE-AMI) study: study protocol for a randomized controlled trial. | remove |
| **688** | Pharmacokinetic interaction between ezetimibe and lovastatin in healthy volunteers. | remove |
| **689** | Differences in action of atorvastatin and ezetimibe in lowering low-density lipoprotein cholesterol and effect on endothelial function: randomized controlled trial. | remove |
| **690** | Effect of fixed-dose combinations of ezetimibe plus rosuvastatin in patients with primary hypercholesterolemia: MRS-ROZE (Multicenter Randomized Study of ROsuvastatin and eZEtimibe). | remove |
| **691** | Efficacy and safety of ezetimibe coadministered with lovastatin in primary hypercholesterolemia. | remove |
| **692** | Safety and efficacy of ezetimibe/simvastatin combination versus atorvastatin alone in adults 65 years of age with hypercholesterolemia and with or at moderately high/high risk for coronary heart disease (the VYTELD study). | remove |
| **693** | Age, abdominal obesity, and baseline high-sensitivity C-reactive protein are associated with low-density lipoprotein cholesterol, non-high-density lipoprotein cholesterol, and apolipoprotein B responses to ezetimibe/simvastatin and atorvastatin in patients with metabolic syndrome. | remove |
| **694** | The Bioequivalence and Effect of Food on the Pharmacokinetics of a Fixed-Dose Combination Tablet Containing Rosuvastatin and Ezetimibe in Healthy Japanese Subjects. | remove |
| **695** | Efficacy and safety of alirocumab, a monoclonal antibody to PCSK9, in statin-intolerant patients: design and rationale of ODYSSEY ALTERNATIVE, a randomized phase 3 trial. | remove |
| **696** | The efficacy and safety of ezetimibe/simvastatin combination compared with intensified lipid-lowering treatment strategies in diabetic subjects with and without metabolic syndrome. | remove |
| **697** | Ezetimibe alone and in combination lowers the concentration of small, dense low-density lipoproteins in type 2 diabetes mellitus. | remove |
| **698** | Effect of atorvastatin monotherapy and low-dose atorvastatin/ezetimibe combination on fasting and postprandial triglycerides in combined hyperlipedemia. | remove |
| **699** | Effects of Combination of Ezetimibe and Rosuvastatin on Coronary Artery Plaque in Patients with Coronary Heart Disease. | remove |
| **700** | Achievement of dual low-density lipoprotein cholesterol and high-sensitivity C-reactive protein targets more frequent with the addition of ezetimibe to simvastatin and associated with better outcomes in IMPROVE-IT. | remove |
| **701** | Lipid lowering efficacy and safety of Ezetimibe combined with rosuvastatin compared with titrating rosuvastatin monotherapy in HIV-positive patients. | remove |
| **702** | Efficacy and safety of coadministration of rosuvastatin, ezetimibe, and colestimide in heterozygous familial hypercholesterolemia. | remove |
| **703** | The effect of ezetimibe and simvastatin on hemostasis in patients with isolated hypercholesterolemia. | remove |
| **704** | Ezetimibe improves endothelial function and inhibits Rho-kinase activity associated with inhibition of cholesterol absorption in humans. | remove |
| **705** | Bioequivalence of an ezetimibe/simvastatin combination tablet and coadministration of ezetimibe and simvastatin as separate tablets in healthy subjects. | remove |
| **706** | Effects of Fixed-dose Combination of Low-intensity Rosuvastatin and Ezetimibe Versus Moderate-intensity Rosuvastatin Monotherapy on Lipid Profiles in Patients With Hypercholesterolemia: A Randomized, Double-blind, Multicenter, Phase III Study. | remove |
| **707** | Rationale, design features, and baseline characteristics: The Heart Institute of Japan-PRoper level of lipid lOwering with Pitavastatin and Ezetimibe in acute coRonary syndrome (HIJ-PROPER). | remove |
| **708** | Influence of metabolic syndrome factors and insulin resistance on the efficacy of ezetimibe/simvastatin and atorvastatin in patients with metabolic syndrome and atherosclerotic coronary heart disease risk. | remove |
| **709** | Effect of Randomized Lipid Lowering With Simvastatin and Ezetimibe on Cataract Development (from the Simvastatin and Ezetimibe in Aortic Stenosis Study). | remove |
| **710** | Extended-release niacin or ezetimibe and carotid intima-media thickness. | remove |
| **711** | Efficacy and safety of ezetimibe 40 mg vs. ezetimibe 10 mg in the treatment of patients with homozygous sitosterolaemia. | remove |
| **712** | Rationale and design of a randomized clinical study to investigate the effect of ezetimibe, a cholesterol absorption inhibitor, on the regression of intracoronary plaque evaluated by non-obstructive angioscopy and ultrasound: The ZIPANGU study. | remove |
| **713** | Intracranial Hemorrhage in the TST Trial. | remove |
| **714** | Goal achievement of HbA1c and LDL-cholesterol in a randomized trial comparing colesevelam with ezetimibe: GOAL-RCT. | remove |
| **715** | More Than 50 Percent Reduction in LDL Cholesterol in Patients With Target LDL <70 mg/dL After a Stroke. | remove |
| **716** | Comparison of Pharmacokinetics and Safety of a Fixed-dose Combination of Rosuvastatin and Ezetimibe Versus Separate Tablets in Healthy Subjects. | remove |
| **717** | Differential effects of short-term lipid lowering with ezetimibe and statins on endothelial function in patients with CAD: clinical evidence for 'pleiotropic' functions of statin therapy. | remove |
| **718** | Benefit and tolerability of the coadministration of ezetimibe and atorvastatin in acute coronary syndrome patients. | remove |
| **719** | Ezetimibe beneficially influences fasting and postprandial triglyceride-rich lipoproteins in type 2 diabetes. | remove |
| **720** | Changes in cholesterol absorption and cholesterol synthesis caused by ezetimibe and/or simvastatin in men. | remove |
| **721** | Combination Therapy of Rosuvastatin and Ezetimibe in Patients with High Cardiovascular Risk. | remove |
| **722** | Efficacy and Safety of Alirocumab Versus Ezetimibe Over 2 Years (from ODYSSEY COMBO II). | remove |
| **723** | The SEAS Trial. | remove |
| **724** | Pharmacodynamic interaction between ezetimibe and rosuvastatin. | remove |
| **725** | Simultaneous quantitation of rosuvastatin and ezetimibe in human plasma by LC-MS/MS: Pharmacokinetic study of fixed-dose formulation and separate tablets. | remove |
| **726** | Cost-effectiveness and Budget Impact of Treatment with Evolocumab Versus Statins and Ezetimibe for Hypercholesterolemia in Spain. | remove |
| **727** | Baseline low-density lipoprotein cholesterol predicts the benefit of adding ezetimibe on statin in statin-naive acute coronary syndrome. | remove |
| **728** | The effect of ezetimibe and simvastatin on monocyte cytokine release in patients with isolated hypercholesterolemia. | remove |
| **729** | Effects of alirocumab on cardiovascular and metabolic outcomes after acute coronary syndrome in patients with or without diabetes: a prespecified analysis of the ODYSSEY OUTCOMES randomised controlled trial. | remove |
| **730** | Pharmacokinetics and bioequivalence of a rosuvastatin/ezetimibe fixed-dose combination tablet versus single agents in healthy male subjects. | remove |
| **731** | Ezetimibe Improves Rosuvastatin Effects on Inflammation and Vascular Endothelial Function in Acute Coronary Syndrome Patients Undergoing PCI. | remove |
| **732** | Effectiveness of ezetimibe added to ongoing statin therapy in modifying lipid profiles and low-density lipoprotein cholesterol goal attainment in patients of different races and ethnicities: a substudy of the Ezetimibe add-on to statin for effectiveness trial. | remove |
| **733** | Intensive statin therapy, used alone or in combination with ezetimibe, improves homocysteine level and lipid peroxidation to a similar degree in patients with coronary artery diseases. | remove |
| **734** | COMBINATION THERAPY EFFECTIVENESS OF EZETIMIBE AND ATORVASTATIN IN PATIENTS WITH ACUTE CORONARY SYNDROME. | remove |
| **735** | Effect of Atorvastatin (10 mg) and Ezetimibe (10 mg) Combination Compared to Atorvastatin (40 mg) Alone on Coronary Atherosclerosis. | remove |
| **736** | Efficacy and safety of coadministration of ezetimibe and statins in elderly patients with primary hypercholesterolaemia. | remove |
| **737** | Effect of 1PC111, a Fixed-dose Combination of Pitavastatin and Ezetimibe, Versus Pitavastatin or Ezetimibe Monotherapy on Lipid Profiles in Patients With Hypercholesterolemia or Mixed Dyslipidemia: A Randomized, Double-blind, Multicenter, Phase III Study. | remove |
| **738** | Efficacy and Tolerability of Ezetimibe/Atorvastatin Fixed-dose Combination Versus Atorvastatin Monotherapy in Hypercholesterolemia: A Phase III, Randomized, Active-controlled Study in Chinese Patients. | remove |
| **739** | RESEARCH (Recognized effect of Statin and ezetimibe therapy for achieving LDL-C Goal), a randomized, doctor-oriented, multicenter trial to compare the effects of higher-dose statin versus ezetimibe-plus-statin on the serum LDL-C concentration of Japanese type-2 diabetes patients design and rationale. | remove |
| **740** | Efficacy and safety of coadministration of ezetimibe and simvastatin in adolescents with heterozygous familial hypercholesterolemia. | remove |
| **741** | Cost-effectiveness of lipid lowering with statins and ezetimibe in chronic kidney disease. | remove |
| **742** | Indices of cholesterol metabolism and relative responsiveness to ezetimibe and simvastatin. | remove |
| **743** | Ezetimibe and simvastatin reduce inflammation, disease activity, and aortic stiffness and improve endothelial function in rheumatoid arthritis. | remove |
| **744** | Effects of ezetimibe on the pharmacodynamics and pharmacokinetics of lovastatin. | remove |
| **745** | Switching from statin monotherapy to ezetimibe/simvastatin or rosuvastatin modifies the relationships between apolipoprotein B, LDL cholesterol, and non-HDL cholesterol in patients at high risk of coronary disease. | remove |
| **746** | Effect of Ezetimibe on Stabilization and Regression of Intracoronary Plaque - The ZIPANGU Study. | remove |
| **747** | Impact of adding ezetimibe to statin to achieve low-density lipoprotein cholesterol goal (from the Clinical Outcomes Utilizing Revascularization and Aggressive Drug Evaluation [COURAGE] trial). | remove |
| **748** | Effectiveness of the addition of ezetimibe to ongoing statin therapy in modifying lipid profiles and attaining low-density lipoprotein cholesterol goals in older and elderly patients: subanalyses of data from a randomized, double-blind, placebo-controlled trial. | remove |
| **749** | Ezetimibe and Rosuvastatin Combination Treatment Can Reduce the Dose of Rosuvastatin Without Compromising Its Lipid-lowering Efficacy. | remove |
| **750** | Efficacy of ezetimibe/simvastatin 10/40 mg compared to doubling the dose of low-, medium- and high-potency statin monotherapy in patients with a recent coronary event. | remove |
| **751** | Effects of statin monotherapy versus statin plus ezetimibe combination on serum uric acid levels. | remove |
| **752** | Pharmacokinetic interactions and tolerability of rosuvastatin and ezetimibe: an open-label, randomized, multiple-dose, crossover study in healthy male volunteers. | remove |
| **753** | Pharmacokinetic interactions and tolerability of rosuvastatin and ezetimibe: an open-label, randomized, multiple-dose, crossover study in healthy male volunteers. | remove |
| **754** | Insulin Prevents Hypercholesterolemia by Suppressing 12α-Hydroxylated Bile Acids. | remove |
| **755** | Evidence for statin pleiotropy in humans: differential effects of statins and ezetimibe on rho-associated coiled-coil containing protein kinase activity, endothelial function, and inflammation. | remove |
| **756** | Rationale and design of LAPLACE-2: a phase 3, randomized, double-blind, placebo- and ezetimibe-controlled trial evaluating the efficacy and safety of evolocumab in subjects with hypercholesterolemia on background statin therapy. | remove |
| **757** | Comparative effects on lipid levels of combination therapy with a statin and extended-release niacin or ezetimibe versus a statin alone (the COMPELL study). | remove |
| **758** | Long-term efficacy and safety of moderate-intensity statin with ezetimibe combination therapy versus high-intensity statin monotherapy in patients with atherosclerotic cardiovascular disease (RACING): a randomised, open-label, non-inferiority trial. | remove |
| **759** | Combination Moderate-Intensity Statin and Ezetimibe Therapy for Elderly Patients With Atherosclerosis. | remove |
| **760** | Moderate-intensity statin with ezetimibe vs. high-intensity statin in patients with diabetes and atherosclerotic cardiovascular disease in the RACING trial. | remove |
| **761** | Ezetimibe Added to Statin Therapy after Acute Coronary Syndromes. | remove |
| **762** | Efficacy and safety of bempedoic acid added to ezetimibe in statin-intolerant patients with hypercholesterolemia: A randomized, placebo-controlled study. | remove |
| **763** | Bempedoic acid plus ezetimibe fixed-dose combination in patients with hypercholesterolemia and high CVD risk treated with maximally tolerated statin therapy. | remove |
| **764** | Efficacy and Tolerability of Evolocumab vs Ezetimibe in Patients With Muscle-Related Statin Intolerance: The GAUSS-3 Randomized Clinical Trial. | remove |
| **765** | Benefit of Adding Ezetimibe to Statin Therapy on Cardiovascular Outcomes and Safety in Patients With Versus Without Diabetes Mellitus: Results From IMPROVE-IT (Improved Reduction of Outcomes: Vytorin Efficacy International Trial). | remove |
| **766** | Moderate-Intensity Statin With Ezetimibe Combination Therapy vs High-Intensity Statin Monotherapy in Patients at Very High Risk of Atherosclerotic Cardiovascular Disease: A Post Hoc Analysis From the RACING Randomized Clinical Trial. | remove |
| **767** | Combination of bempedoic acid, ezetimibe, and atorvastatin in patients with hypercholesterolemia: A randomized clinical trial. | remove |
| **768** | Yield of Dual Therapy With Statin and Ezetimibe in the Treat Stroke to Target Trial. | remove |
| **769** | A Phase III, Multicenter, Randomized, Double-blind, Active Comparator Clinical Trial to Compare the Efficacy and Safety of Combination Therapy With Ezetimibe and Rosuvastatin Versus Rosuvastatin Monotherapy in Patients With Hypercholesterolemia: I-ROSETTE (Ildong Rosuvastatin & Ezetimibe for Hypercholesterolemia) Randomized Controlled Trial. | remove |
| **770** | Obicetrapib plus ezetimibe as an adjunct to high-intensity statin therapy: A randomized phase 2 trial. | remove |
| **771** | Ezetimibe combination therapy with statin for non-alcoholic fatty liver disease: an open-label randomized controlled trial (ESSENTIAL study). | remove |
| **772** | Small Interfering RNA to Reduce Lipoprotein(a) in Cardiovascular Disease. | remove |
| **773** | A Randomized, Multicenter, Double-blind, Placebo-Controlled Study to Evaluate the Efficacy and Safety of a Quadruple Combination of Amlodipine, Losartan, Rosuvastatin, and Ezetimibe in Patients with Concomitant Essential Hypertension and Dyslipidemia. | remove |
| **774** | Impact of Dual Lipid-Lowering Strategy With Ezetimibe and Atorvastatin on Coronary Plaque Regression in Patients With Percutaneous Coronary Intervention: The Multicenter Randomized Controlled PRECISE-IVUS Trial. | remove |
| **775** | The Efficacy and Safety of Moderate-Intensity Rosuvastatin with Ezetimibe versus High-Intensity Rosuvastatin in High Atherosclerotic Cardiovascular Disease Risk Patients with Type 2 Diabetes Mellitus: A Randomized, Multicenter, Open, Parallel, Phase 4 Study. | remove |
| **776** | Efficacy and Safety of Single-Pill Combination of Rosuvastatin and Ezetimibe in Chinese Patients with Primary Hypercholesterolemia Inadequately Controlled by Statin Treatment (ROZEL): A Randomized, Double-Blind, Double Dummy, Active-Controlled Phase 3 Clinical Trial. | remove |
| **777** | Effect of rosuvastatin 20 mg versus rosuvastatin 5 mg plus ezetimibe on statin side-effects in elderly patients with atherosclerotic cardiovascular disease: Rationale and design of a randomized, controlled SaveSAMS trial. | remove |
| **778** | Association between Serum Oxysterols and Coronary Plaque Regression during Lipid-Lowering Therapy with Statin and Ezetimibe: Insights from the CuVIC Trial. | remove |
| **779** | Baseline Low-Density Lipoprotein Cholesterol and Clinical Outcomes of Combining Ezetimibe With Statin Therapy in IMPROVE-IT. | remove |
| **780** | Comparison of statin plus ezetimibe with double-dose statin on lipid profiles and inflammation markers. | remove |
| **781** | Effectiveness and Safety of Novel Nutraceutical Formulation Added to Ezetimibe in Statin-Intolerant Hypercholesterolemic Subjects with Moderate-to-High Cardiovascular Risk. | remove |
| **782** | A Comparison of Two LDL Cholesterol Targets after Ischemic Stroke. | remove |
| **783** | The influence of statin monotherapy and statin-ezetimibe combined therapy on FoxP3 and IL 10 mRNA expression in patients with coronary artery disease. | remove |
| **784** | Moderate-intensity statin plus ezetimibe vs high-intensity statin according to baseline LDL-C in the treatment of atherosclerotic cardiovascular disease: A post-hoc analysis of the RACING randomized trial. | remove |
| **785** | ODYSSEY EAST: Alirocumab efficacy and safety vs ezetimibe in high cardiovascular risk patients with hypercholesterolemia and on maximally tolerated statin in China, India, and Thailand. | remove |
| **786** | A 52-week placebo-controlled trial of evolocumab in hyperlipidemia. | remove |
| **787** | Efficacy, Safety, and Tolerability of Inclisiran in Patients With Homozygous Familial Hypercholesterolemia: Results From the ORION-5 Randomized Clinical Trial. | remove |
| **788** | Efficacy and Safety of Pitavastatin/Ezetimibe Fixed-Dose Combination vs. Pitavastatin: Phase III, Double-Blind, Randomized Controlled Trial. | remove |
| **789** | Have We Learnt all from IMPROVE-IT? Part I. Core Results and Subanalyses on the Effects of Ezetimibe Added to Statin Therapy Related to Age, Gender and Selected Chronic Diseases (Kidney Disease, Diabetes Mellitus and Non-Alcoholic Fatty Liver Disease). | remove |
| **790** | Effect of the Early Application of Evolocumab on Blood Lipid Profile and Cardiovascular Prognosis in Patients with Extremely High-Risk Acute Coronary Syndrome. | remove |
| **791** | Effects of ezetimibe, simvastatin, atorvastatin, and ezetimibe-statin therapies on non-cholesterol sterols in patients with primary hypercholesterolemia. | remove |
| **792** | Efficacy and Safety of Adding Ezetimibe to Statin Therapy Among Women and Men: Insight From IMPROVE-IT (Improved Reduction of Outcomes: Vytorin Efficacy International Trial). | remove |
| **793** | A Multicenter, Randomized, Double-blind, Active-controlled, Factorial Design, Phase III Clinical Trial to Evaluate the Efficacy and Safety of Combination Therapy of Pitavastatin and Ezetimibe Versus Monotherapy of Pitavastatin in Patients With Primary Hypercholesterolemia. | remove |
| **794** | Effectiveness of low-intensity atorvastatin 5 mg and ezetimibe 10 mg combination therapy compared with moderate-intensity atorvastatin 10 mg monotherapy: A randomized, double-blinded, multi-center, phase III study. | remove |
| **795** | Pharmacokinetic Interaction Among Ezetimibe, Rosuvastatin, and Telmisartan. | remove |
| **796** | The effects of statin monotherapy and low-dose statin/ezetimibe on lipoprotein-associated phospholipase A2. | remove |
| **797** | Effect of simvastatin and ezetimibe on suPAR levels and outcomes. | remove |
| **798** | Pharmacokinetic and pharmacodynamic interaction between ezetimibe and rosuvastatin in healthy male subjects. | remove |
| **799** | Safety and effectiveness of the association ezetimibe-statin (E-S) versus high dose rosuvastatin after acute coronary syndrome: the SAFE-ES study. | remove |
| **800** | Efficacy and safety of ezetimibe added to ongoing statin therapy for treatment of patients with primary hypercholesterolemia. | remove |
| **801** | Usefulness of Nutraceuticals (Armolipid Plus) Versus Ezetimibe and Combination in Statin-Intolerant Patients With Dyslipidemia With Coronary Heart Disease. | remove |
| **802** | Efficacy and safety of alirocumab vs ezetimibe in statin-intolerant patients, with a statin rechallenge arm: The ODYSSEY ALTERNATIVE randomized trial. | remove |
| **803** | Pharmacokinetic Interactions and Tolerability of Rosuvastatin and Ezetimibe: A Randomized, Phase 1, Crossover Study in Healthy Chinese Participants. | remove |
| **804** | A comparison of the efficacy of combined ezetimibe and statin therapy with doubling of statin dose in patients with remnant lipoproteinemia on previous statin therapy. | remove |
| **805** | Niacin in patients with low HDL cholesterol levels receiving intensive statin therapy. | remove |
| **806** | Ezetimibe and simvastatin combination inhibits and reverses the pro-inflammatory and pro-atherogenic effects of cream in obese patients. | remove |
| **807** | Study of Heart and Renal Protection (SHARP): randomized trial to assess the effects of lowering low-density lipoprotein cholesterol among 9,438 patients with chronic kidney disease. | remove |
| **808** | Efficacy, safety and tolerability of ongoing statin plus ezetimibe versus doubling the ongoing statin dose in hypercholesterolemic Taiwanese patients: an open-label, randomized clinical trial. | remove |
| **809** | Usefulness of Low-Dose Statin Plus Ezetimibe and/or Nutraceuticals in Patients With Coronary Artery Disease Intolerant to High-Dose Statin Treatment. | remove |
| **810** | Efficacy and safety of ezetimibe plus orlistat or rimonabant in statin-intolerant nondiabetic overweight/obese patients with dyslipidemia. | remove |
| **811** | Effect of evolocumab or ezetimibe added to moderate- or high-intensity statin therapy on LDL-C lowering in patients with hypercholesterolemia: the LAPLACE-2 randomized clinical trial. | remove |
| **812** | The benefit of adding ezetimibe to statin therapy in patients with prior coronary artery bypass graft surgery and acute coronary syndrome in the IMPROVE-IT trial. | remove |
| **813** | Ezetimibe added to ongoing statin therapy improves LDL-C goal attainment and lipid profile in patients with diabetes or metabolic syndrome. | remove |
| **814** | Short-term ezetimibe is well tolerated and effective in combination with statin therapy to treat elevated LDL cholesterol in HIV-infected patients. | remove |
| **815** | Design and rationale of the GAUSS-2 study trial: a double-blind, ezetimibe-controlled phase 3 study of the efficacy and tolerability of evolocumab (AMG 145) in subjects with hypercholesterolemia who are intolerant of statin therapy. | remove |
| **816** | Impact of statin-ezetimibe combination on coronary atheroma plaque in patients with and without chronic kidney disease - Sub-analysis of PRECISE-IVUS trial. | remove |
| **817** | Effects of simvastatin and ezetimibe on interleukin-6 and high-sensitivity C-reactive protein. | remove |
| **818** | Pharmacokinetic Interaction Between Telmisartan and Rosuvastatin/Ezetimibe After Multiple Oral Administration in Healthy Subjects. | remove |
| **819** | Evolocumab vs. Ezetimibe in Statin-Intolerant Hyperlipidemic Japanese Patients: Phase 3 GAUSS-4 Trial. | remove |
| **820** | Effects of ezetimibe added to statin therapy on markers of cholesterol absorption and synthesis and LDL-C lowering in hyperlipidemic patients. | remove |
| **821** | Colesevelam added to combination therapy with a statin and ezetimibe in patients with familial hypercholesterolemia: a 12-week, multicenter, randomized, double-blind, controlled trial. | remove |
| **822** | Comparison of the efficacy and safety of statin and statin/ezetimibe therapy after coronary stent implantation in patients with stable angina. | remove |
| **823** | High-dose statin monotherapy versus low-dose statin/ezetimibe combination on fasting and postprandial lipids and endothelial function in obese patients with the metabolic syndrome: The PANACEA study. | remove |
| **824** | Biomarkers and Clinical Cardiovascular Outcomes With Ezetimibe in the IMPROVE-IT Trial. | remove |
| **825** | Efficacy and Safety of Ezetimibe and Rosuvastatin Combination Therapy Versus Those of Rosuvastatin Monotherapy in Patients With Primary Hypercholesterolemia. | remove |
| **826** | Effects of Ezetimibe-Statin Combination Therapy on Coronary Atherosclerosis in Acute Coronary Syndrome. | remove |
| **827** | Comparative efficacy and adverse effects of the addition of ezetimibe to statin versus statin titration in chronic kidney disease patients. | remove |
| **828** | Effects of simvastatin, ezetimibe and simvastatin/ezetimibe on mitochondrial function and leukocyte/endothelial cell interactions in patients with hypercholesterolemia. | remove |
| **829** | Comparative effects on lipid levels of combination therapy with a statin and extended-release niacin or ezetimibe versus a statin alone (the COMPELL study). | remove |
| **830** | Rationale and design of LAPLACE-2: a phase 3, randomized, double-blind, placebo- and ezetimibe-controlled trial evaluating the efficacy and safety of evolocumab in subjects with hypercholesterolemia on background statin therapy. | remove |
| **831** | Evidence for statin pleiotropy in humans: differential effects of statins and ezetimibe on rho-associated coiled-coil containing protein kinase activity, endothelial function, and inflammation. | remove |
| **832** | Pharmacokinetic interactions and tolerability of rosuvastatin and ezetimibe: an open-label, randomized, multiple-dose, crossover study in healthy male volunteers. | remove |
| **833** | Effects of statin monotherapy versus statin plus ezetimibe combination on serum uric acid levels. | remove |
| **834** | Efficacy of ezetimibe/simvastatin 10/40 mg compared to doubling the dose of low-, medium- and high-potency statin monotherapy in patients with a recent coronary event. | remove |
| **835** | Effectiveness of Rosuvastatin Combined with Ezetimibe in the Treatment of Unstable Angina Combined with Type 2 Diabetes Mellitus in Older Adults | remove |
| **836** | Evaluation of ezetimibe combined with Rosuvastatin in the treatment of diabetes mellitus complicated with hyperlipidemia | remove |
| **837** | Study on lipid regulation therapy of low-dose ezetimibe combined with resuvastatin in patients with diabetes mellitus combined with coronary artery disease | remove |
| **838** | Clinical efficacy and early prognosis of Rosuvastatin combined with ezetimibe in the treatment of acute coronary syndrome | remove |
| **839** | Clinical efficacy analysis of Rosuvastatin combined with ezetimibe in the treatment of diabetes mellitus complicated with hyperlipidemia in elderly people | remove |
| **840** | Clinical efficacy of ezetimibe combined with resuvastatin in the treatment of unstable angina combined with type 2 diabetes mellitus | remove |
| **841** | Clinical observation on small-dose Rosuvastatin combined with ezetimibe in the treatment of hyperlipidemia | remove |
| **842** | Effect of Rosuvastatin combined with ezetimibe in patients with early diabetic nephropathy | remove |
| **843** | Effectiveness of Rosuvastatin Combined with Ezetimibe in the Treatment of Unstable Angina Combined with Type 2 Diabetes Mellitus in Older Adults | remove |
| **844** | A study of the therapeutic effect of atorvastatin calcium combined with ezetimibe in coronary heart disease | remove |
| **845** | Analysis of the effects of atorvastatin calcium and ezetimibe drug therapy for coronary artery disease on patients' lipid levels and adverse effects | remove |
| **846** | Analysis of 108 cases of coronary heart disease treated with atorvastatin calcium combined with ezetimibe medication | remove |
| **847** | Investigating the therapeutic efficacy and clinical safety of atorvastatin calcium combined with a new ezetimibe drug in coronary heart disease | remove |
| **848** | Evaluation of ezetimibe combined with Rosuvastatin in the treatment of diabetes mellitus complicated with hyperlipidemia | remove |
| **849** | Efficacy and safety study of atorvastatin combined with ezetimibe versus atorvastatin alone in patients with ASCVD | remove |
| **850** | Clinical efficacy and early prognosis of Rosuvastatin combined with ezetimibe in the treatment of acute coronary syndrome | remove |
| **851** | Clinical efficacy of ezetimibe combined with atorvastatin in the treatment of type 2 diabetes mellitus combined with hyperlipidemia | remove |
| **852** | Clinical analysis of simvastatin combined with ezetimibe regimen in the treatment of acute coronary syndromes | remove |
| **853** | Clinical efficacy analysis of Rosuvastatin combined with ezetimibe in the treatment of diabetes mellitus complicated with hyperlipidemia in elderly people | remove |
| **854** | Clinical efficacy of early atorvastatin combined with ezetimibe in the treatment of acute coronary syndromes | remove |
| **855** | Clinical efficacy of ezetimibe combined with resuvastatin in the treatment of unstable angina combined with type 2 diabetes mellitus | remove |
| **856** | Clinical observation on small-dose Rosuvastatin combined with ezetimibe in the treatment of hyperlipidemia | remove |
| **857** | Effect of Rosuvastatin combined with ezetimibe in patients with early diabetic nephropathy | remove |
| **858** | Meta-analysis of lipid-lowering efficacy and safety of ezetimibe in combination with simvastatin | remove |
| **859** | Clinical analysis of ezetimibe combined with simvastatin in the treatment of hyperlipidemia | remove |
| **860** | Relation of Lipid-Lowering Therapy to Need for Aortic Valve Replacement in Patients With Asymptomatic Mild to Moderate Aortic Stenosis. | remove |
| **861** | Comparison of the effects of combination atorvastatin (40 mg) + ezetimibe (10 mg) versus atorvastatin (40 mg) alone on secretory phospholipase A2 activity in patients with stable coronary artery disease or coronary artery disease equivalent. | remove |
| **862** | Effects of lipid-lowering drugs on high-density lipoprotein subclasses in healthy men-a randomized trial. | remove |
| **863** | Quantitative and qualitative pleiotropic differences between Simvastatin single and Vytorin combination therapy in hypercholesterolemic subjects. | remove |
| **864** | Reduction in Total Cardiovascular Events With Ezetimibe/Simvastatin Post-Acute Coronary Syndrome: The IMPROVE-IT Trial. | remove |
| **865** | Effect of statins alone versus statins plus ezetimibe on carotid atherosclerosis in type 2 diabetes: the SANDS (Stop Atherosclerosis in Native Diabetics Study) trial. | remove |
| **866** | Impact of stroke volume on cardiovascular risk during progression of aortic valve stenosis. | remove |
| **867** | Impact of cholesterol lowering treatment on plasma kynurenine and tryptophan concentrations in chronic kidney disease: relationship with oxidative stress improvement. | remove |
| **868** | Effects of lipid-lowering drugs on irisin in human subjects in vivo and in human skeletal muscle cells ex vivo. | remove |
| **869** | Dyslipidemia in HIV-positive patients: a randomized, controlled, prospective study on ezetimibe+fenofibrate versus pravastatin monotherapy. | remove |
| **870** | Lipid profile associated with coronary plaque regression in patients with acute coronary syndrome: Subanalysis of PRECISE-IVUS trial. | remove |
| **871** | Treatment effect of alirocumab according to age group, smoking status, and hypertension: Pooled analysis from 10 randomized ODYSSEY studies. | remove |
| **872** | A multi-center, open label, crossover designed prospective study evaluating the effects of lipid lowering treatment on steroid synthesis in patients with Type 2 diabetes (MODEST Study). | remove |
| **873** | [Effect of simvastatin plus inulin in comparison with simvastatin plus ezetimibe on the treatment of mixed dyslipidemia]. | remove |
| **874** | Cholesterol lowering treatment restores blood global DNA methylation in chronic kidney disease (CKD) patients. | remove |
| **875** | Fluvastatin/fenofibrate vs. simvastatin/ezetimibe in patients with metabolic syndrome: different effects on LDL-profiles. | remove |
| **876** | A highly bioavailable omega-3 free fatty acid formulation improves the cardiovascular risk profile in high-risk, statin-treated patients with residual hypertriglyceridemia (the ESPRIT trial). | remove |
| **877** | High-dose simvastatin exhibits enhanced lipid-lowering effects relative to simvastatin/ezetimibe combination therapy. | remove |
| **878** | Antihypertensive Treatment With β-Blockade in Patients With Asymptomatic Aortic Stenosis and Association With Cardiovascular Events. | remove |
| **879** | Targeting high-sensitivity C-reactive protein levels in acute coronary syndrome patients undergoing contemporary lipid-lowering therapy: a sub-analysis of the HIJ-PROPER trial. | remove |
| **880** | Effects of rosuvastatin with or without ezetimibe on clinical outcomes in patients undergoing elective vascular surgery: results of a pilot study. | remove |
| **881** | Competing Risks of Cardiovascular Versus Noncardiovascular Death During Long-Term Follow-Up After Acute Coronary Syndromes. | remove |
| **882** | SREBP-1c gene polymorphism is associated with increased inhibition of cholesterol-absorption in response to ezetimibe treatment. | remove |
| **883** | Risk of hospitalized rhabdomyolysis associated with lipid-lowering drugs in a real-world clinical setting. | remove |
| **884** | Effects of rosuvastatin vs. simvastatin/ezetimibe on arterial wall stiffness in patients with coronary artery disease. | remove |
| **885** | Relation of Left Ventricular Mass to Prognosis in Initially Asymptomatic Mild to Moderate Aortic Valve Stenosis. | remove |
| **886** | Lipid-lowering therapy does not affect the postprandial drop in high density lipoprotein-cholesterol (HDL-c) plasma levels in obese men with metabolic syndrome: a randomized double blind crossover trial. | remove |
| **887** | Cholesterol lowering is more important than pleiotropic effects of statins for endothelial function in patients with dysglycaemia and coronary artery disease. | remove |
| **888** | Main differences between two highly effective lipid-lowering therapies in subclasses of lipoproteins in patients with acute myocardial infarction. | remove |
| **889** | Asymmetric dimethylarginine and the effect of folate substitution in children with familial hypercholesterolemia and diabetes mellitus type 1. | remove |
| **890** | Muscle Complaints or Events in Patients Randomized to Simvastatin or Ezetimibe/Simvastatin. | remove |
| **891** | Influence of ezetimibe in addition to high-dose atorvastatin therapy on plaque composition in patients with ST-segment elevation myocardial infarction assessed by serial: Intravascular ultrasound with iMap: the OCTIVUS trial. | remove |
| **892** | Low-flow aortic stenosis in asymptomatic patients: valvular-arterial impedance and systolic function from the SEAS Substudy. | remove |
| **893** | Predictive Value of Baseline High-Sensitivity C-Reactive Protein Level and Renal Function for Patients With Acute Coronary Syndrome Undergoing Aggressive Lipid-Lowering Therapy: A Subanalysis of HIJ-PROPER. | remove |
| **894** | Effects of lipid-lowering treatment on circulating microparticles in patients with diabetes mellitus and chronic kidney disease. | remove |
| **895** | Low-density lipoprotein lowering does not improve calf muscle perfusion, energetics, or exercise performance in peripheral arterial disease. | remove |
| **896** | Effects of evolocumab (AMG 145), a monoclonal antibody to PCSK9, in hypercholesterolemic, statin-treated Japanese patients at high cardiovascular risk--primary results from the phase 2 YUKAWA study. | remove |
| **897** | Ezetimibe/simvastatin 10/40 mg versus atorvastatin 40 mg in high cardiovascular risk patients with primary hypercholesterolemia: a randomized, double-blind, active-controlled, multicenter study. | remove |
| **898** | Global left ventricular load in asymptomatic aortic stenosis: covariates and prognostic implication (the SEAS trial). | remove |
| **899** | Plaque REgression with Cholesterol absorption Inhibitor or Synthesis inhibitor Evaluated by IntraVascular UltraSound (PRECISE-IVUS Trial): Study protocol for a randomized controlled trial. | remove |
| **900** | Effect of extended-release niacin on new-onset diabetes among hyperlipidemic patients treated with ezetimibe/simvastatin in a randomized controlled trial. | remove |
| **901** | Concordance between plasma apolipoprotein B levels and cholesterol indices among patients receiving statins and nonstatin treatment: Post-hoc analyses from the U.K. InPractice study. | remove |
| **902** | Ezetimibe added to atorvastatin compared with doubling the atorvastatin dose in patients at high risk for coronary heart disease with diabetes mellitus, metabolic syndrome or neither. | remove |
| **903** | Are post-treatment low-density lipoprotein subclass pattern analyses potentially misleading? | remove |
| **904** | The role of soluble fiber intake in patients under highly effective lipid-lowering therapy. | remove |
| **905** | Statin-induced immunomodulation alters peripheral invariant natural killer T-cell prevalence in hyperlipidemic patients. | remove |
| **906** | Rosuvastatin but not ezetimibe improves endothelial function in patients with heart failure, by mechanisms independent of lipid lowering. | remove |
| **907** | [Therapy of familial hypercholesterolemia with or without ezetimibe]. | remove |
| **908** | On-treatment analysis of the Improved Reduction of Outcomes: Vytorin Efficacy International Trial (IMPROVE-IT). | remove |
| **909** | Ezetimibe + simvastatin versus doubling the dose of simvastatin in high cardiovascular risk diabetics: a multicenter, randomized trial (the LEAD study). | remove |
| **910** | The acute impact of high-dose lipid-lowering treatment on endothelial progenitor cells in patients with coronary artery disease-The REMEDY-EPC early substudy. | remove |
| **911** | Lipid lowering versus pleiotropic effects of statins on skin microvascular function in patients with dysglycaemia and coronary artery disease. | remove |
| **912** | Relationship of baseline HDL subclasses, small dense LDL and LDL triglyceride to cardiovascular events in the AIM-HIGH clinical trial. | remove |
| **913** | Pathologic Intimal Thickening Plaque Phenotype: Not as Innocent as Previously Thought. A Serial 3D Intravascular Ultrasound Virtual Histology Study. | remove |
| **914** | Effects of adding ezetimibe to fluvastatin on kidney function in patients with hypercholesterolemia: a randomized control trial. | remove |
| **915** | The Effect of Combined Ezetimibe/Atorvastatin Therapy vs. Atorvastatin Monotherapy on the Erythrocyte Membrane Structure in Patients with Coronary Artery Disease: A Pilot Study. | remove |
| **916** | Effect of Alirocumab on Lipoprotein(a) Over ≥1.5 Years (from the Phase 3 ODYSSEY Program). | remove |
| **917** | Efficacy of cholesterol uptake inhibition added to statin therapy among subjects following a low-carbohydrate diet: a randomized controlled trial. | remove |
| **918** | LDL-C goal attainment with ezetimibe plus simvastatin coadministration vs atorvastatin or simvastatin monotherapy in patients at high risk of CHD. | remove |
| **919** | One-year cholesterol lowering treatment reduces medial temporal lobe atrophy and memory decline in stroke-free elderly with atrial fibrillation: evidence from a parallel group randomized trial. | remove |
| **920** | Efficacy and Safety of the Cholesteryl Ester Transfer Protein Inhibitor Evacetrapib in Combination With Atorvastatin in Japanese Patients With Primary Hypercholesterolemia. | remove |
| **921** | Impact of pressure recovery on echocardiographic assessment of asymptomatic aortic stenosis: a SEAS substudy. | remove |
| **922** | Open-label therapy with alirocumab in patients with heterozygous familial hypercholesterolemia: Results from three years of treatment. | remove |
| **923** | Effect of two lipid-lowering strategies on high-density lipoprotein function and some HDL-related proteins: a randomized clinical trial. | remove |
| **924** | A randomised placebo-controlled double-blind trial to evaluate lipid-lowering pharmacotherapy on proteolysis and inflammation in abdominal aortic aneurysms. | remove |
| **925** | Lipoprotein(a)-cholesterol levels estimated by vertical auto profile correlate poorly with Lp(a) mass in hyperlipidemic subjects: Implications for clinical practice interpretation of Lp(a)-mediated risk. | remove |
| **926** | Comparison of the effects of simvastatin vs. rosuvastatin vs. simvastatin/ezetimibe on parameters of insulin resistance. | remove |
| **927** | The role of niacin in raising high-density lipoprotein cholesterol to reduce cardiovascular events in patients with atherosclerotic cardiovascular disease and optimally treated low-density lipoprotein cholesterol: baseline characteristics of study participants. The Atherothrombosis Intervention in Metabolic syndrome with low HDL/high triglycerides: impact on Global Health outcomes (AIM-HIGH) trial. | remove |
| **928** | The role of niacin in raising high-density lipoprotein cholesterol to reduce cardiovascular events in patients with atherosclerotic cardiovascular disease and optimally treated low-density lipoprotein cholesterol: baseline characteristics of study participants. The Atherothrombosis Intervention in Metabolic syndrome with low HDL/high triglycerides: impact on Global Health outcomes (AIM-HIGH) trial. | remove |
| **929** | Assessing Optimal Blood Pressure in Patients With Asymptomatic Aortic Valve Stenosis: The Simvastatin Ezetimibe in Aortic Stenosis Study (SEAS). | remove |
| **930** | Effect of omega-3 fatty acid supplementation on arterial elasticity in patients with familial hypercholesterolaemia on statin therapy. | remove |
| **931** | LDL-C goal attainment with the addition of ezetimibe to ongoing simvastatin treatment in coronary heart disease patients with hypercholesterolemia. | remove |
| **932** | Oxidative stress improvement is associated with increased levels of taurine in CKD patients undergoing lipid-lowering therapy. | remove |
| **933** | Low-density lipoprotein cholesterol-lowering effects of AMG 145, a monoclonal antibody to proprotein convertase subtilisin/kexin type 9 serine protease in patients with heterozygous familial hypercholesterolemia: the Reduction of LDL-C with PCSK9 Inhibition in Heterozygous Familial Hypercholesterolemia Disorder (RUTHERFORD) randomized trial. | remove |
| **934** | The effect of adding ezetimibe to rosuvastatin on renal function in patients undergoing elective vascular surgery. | remove |
| **935** | Effects of lipid-lowering treatment on platelet reactivity and platelet-leukocyte aggregation in diabetic patients without and with chronic kidney disease: a randomized trial. | remove |
| **936** | The effect of adding ezetimibe to rosuvastatin on renal function in patients undergoing elective vascular surgery. | remove |
| **937** | The effect of adding ezetimibe to rosuvastatin on renal function in patients undergoing elective vascular surgery. | remove |
| **938** | Effect of simvastatin/ezetimibe 10/10 mg versus simvastatin 40 mg on serum vitamin D levels. | remove |
| **939** | Low incidence of paradoxical reductions in HDL-C levels in dyslipidemic patients treated with fenofibrate alone or in combination with ezetimibe or ezetimibe/simvastatin. | remove |
| **940** | Bempedoic acid plus ezetimibe fixed-dose combination in patients with hypercholesterolemia and high CVD risk treated with maximally tolerated statin therapy. | remove |
| **941** | Efficacy and Tolerability of Evolocumab vs Ezetimibe in Patients With Muscle-Related Statin Intolerance: The GAUSS-3 Randomized Clinical Trial. | remove |
| **942** | Benefit of Adding Ezetimibe to Statin Therapy on Cardiovascular Outcomes and Safety in Patients With Versus Without Diabetes Mellitus: Results From IMPROVE-IT (Improved Reduction of Outcomes: Vytorin Efficacy International Trial). | remove |
| **943** | Moderate-Intensity Statin With Ezetimibe Combination Therapy vs High-Intensity Statin Monotherapy in Patients at Very High Risk of Atherosclerotic Cardiovascular Disease: A Post Hoc Analysis From the RACING Randomized Clinical Trial. | remove |
| **944** | Long-term efficacy and safety of moderate-intensity statin with ezetimibe combination therapy versus high-intensity statin monotherapy in patients with atherosclerotic cardiovascular disease (RACING): a randomised, open-label, non-inferiority trial. | remove |
| **945** | Treat-to-Target or High-Intensity Statin in Patients With Coronary Artery Disease: A Randomized Clinical Trial. | remove |
| **946** | Effect of Alirocumab Added to High-Intensity Statin Therapy on Coronary Atherosclerosis in Patients With Acute Myocardial Infarction: The PACMAN-AMI Randomized Clinical Trial. | remove |
| **947** | Combination Moderate-Intensity Statin and Ezetimibe Therapy for Elderly Patients With Atherosclerosis. | remove |
| **948** | Beta-Blockers after Myocardial Infarction and Preserved Ejection Fraction. | remove |
| **949** | Long-term efficacy and safety of moderate-intensity statin with ezetimibe combination therapy versus high-intensity statin monotherapy in patients with atherosclerotic cardiovascular disease (RACING): a randomised, open-label, non-inferiority trial. | remove |
| **950** | Treat-to-Target or High-Intensity Statin in Patients With Coronary Artery Disease: A Randomized Clinical Trial. | remove |
| **951** | Effect of Alirocumab Added to High-Intensity Statin Therapy on Coronary Atherosclerosis in Patients With Acute Myocardial Infarction: The PACMAN-AMI Randomized Clinical Trial. | remove |
| **952** | Combination Moderate-Intensity Statin and Ezetimibe Therapy for Elderly Patients With Atherosclerosis. | remove |
| **953** | Beta-Blockers after Myocardial Infarction and Preserved Ejection Fraction. | remove |
| **954** | Effect of Statin Therapy on Outcomes of Patients With Acute Ischemic Stroke and Atrial Fibrillation. | remove |
| **955** | Pharmacological Inhibition of CETP (Cholesteryl Ester Transfer Protein) Increases HDL (High-Density Lipoprotein) That Contains ApoC3 and Other HDL Subspecies Associated With Higher Risk of Coronary Heart Disease. | remove |
| **956** | Effect of Eicosapentaenoic and Docosahexaenoic Acids Added to Statin Therapy on Coronary Artery Plaque in Patients With Coronary Artery Disease: A Randomized Clinical Trial. | remove |
| **957** | Evolocumab treatment in patients with HIV and hypercholesterolemia/mixed dyslipidemia: BEIJERINCK study design and baseline characteristics. | remove |
| **958** | Associations between lower levels of low-density lipoprotein cholesterol and cardiovascular events in very high-risk patients: Pooled analysis of nine ODYSSEY trials of alirocumab versus control. | remove |
| **959** | Coronary atheroma volume and cardiovascular events during maximally intensive statin therapy. | remove |
| **960** | Effects of atorvastatin 20 mg, rosuvastatin 10 mg, and atorvastatin/ezetimibe 5 mg/5 mg on lipoproteins and glucose metabolism. | remove |
| **961** | Additive effects of plant sterols supplementation in addition to different lipid-lowering regimens. | remove |
| **962** | Changes in lipoproteins associated with lipid-lowering and antiplatelet strategies in patients with acute myocardial infarction. | remove |
| **963** | Modeling Statin-Induced Reductions of Cardiovascular Events in Primary Prevention: A VOYAGER Meta-Analysis. | remove |
| **964** | Comparison of the effect of simvastatin versus simvastatin/ezetimibe versus rosuvastatin on markers of inflammation and oxidative stress in subjects with hypercholesterolemia. | remove |
| **965** | The efficacy and safety of ezetimibe/simvastatin combination compared with intensified lipid-lowering treatment strategies in diabetic subjects with and without metabolic syndrome. | remove |
| **966** | Long-term efficacy and safety of moderate-intensity statin with ezetimibe combination therapy versus high-intensity statin monotherapy in patients with atherosclerotic cardiovascular disease (RACING): a randomised, open-label, non-inferiority trial. | integrate |
| **967** | Lipid-Lowering Efficacy of Combination Therapy With Moderate-Intensity Statin and Ezetimibe Versus High-Intensity Statin Monotherapy: A Randomized, Open-Label, Non-Inferiority Trial From Korea. | integrate |
| **968** | Moderate-intensity statin plus ezetimibe vs high-intensity statin according to baseline LDL-C in the treatment of atherosclerotic cardiovascular disease: A post-hoc analysis of the RACING randomized trial. | integrate |
| **969** | Effects of Rosuvastatin combined with ezetimibe on endoplasmic reticulum stress in vascular endothelial cells of patients with atherosclerosis. | integrate |
| **970** | Randomized, Double-blind, Active Comparator Clinical Trial to Compare the Efficacy and Safety of Combination Therapy With Ezetimibe and Rosuvastatin Versus Rosuvastatin Monotherapy in Patients With Hypercholesterolemia: I-ROSETTE (Ildong Rosuvastatin & Ezetimibe for Hypercholesterolemia) Randomized Controlled Trial. | integrate |
| **971** | Clinical efficacy of Rosuvastatin combined with ezetimibe in post-PCI patients. | integrate |
| **972** | A Phase III, Multicenter, Randomized, Double-blind, Active Comparator Clinical Trial to Compare the Efficacy and Safety of Combination Therapy With Ezetimibe and Rosuvastatin Versus Rosuvastatin Monotherapy in Patients With Hypercholesterolemia: I-ROSETTE (Ildong Rosuvastatin & Ezetimibe for Hypercholesterolemia) Randomized Controlled Trial. | integrate |
| **973** | Combination Moderate-Intensity Statin and Ezetimibe Therapy for Elderly Patients With Atherosclerosis. Journal of the American College of Cardiology. | integrate |
| **974** | Moderate-intensity statin with ezetimibe vs. high-intensity statin in patients with diabetes and atherosclerotic cardiovascular disease in the RACING trial. | integrate |
| **975** | Effect of fixed‐dose combinations of ezetimibe plus rosuvastatin in patients with primary hypercholesterolemia: MRS‐ROZE (Multicenter Randomized Study of ROsuvastatin and eZEtimibe). | integrate |
| **976** | Efficacy and Safety of Ezetimibe and Rosuvastatin Combination Therapy Versus Those of Rosuvastatin Monotherapy in Patients With Primary Hypercholesterolemia. Clinical Therapeutics. | integrate |
| **977** | Effect of moderate-intensity statin with ezetimibe combination vs. high-intensity statin therapy according to sex in patients with atherosclerosis. | integrate |
| **978** | Moderate-Intensity Statin With Ezetimibe Combination Therapy vs High-Intensity Statin Monotherapy in Patients at Very High Risk of Atherosclerotic Cardiovascular Disease. | integrate |
| **979** | Evaluation of Two Different Therapies of Rosuvastatin Calcium Tablets on Vulnerable Carotid Plaques in Patients with Cerebral Infarction due to Large- artery. | integrate |
| **980** | Comparison of efficacy and safety of Rosuvastatin calcium tablets combined with ezetimibe tablets in the treatment of hyperlipidemia. | integrate |
| **981** | The Efficacy and Safety of Moderate-Intensity Rosuvastatin with Ezetimibe versus High-Intensity Rosuvastatin in High Atherosclerotic Cardiovascular Disease Risk Patients with Type 2 Diabetes Mellitus: A Randomized, Multicenter, Open, Parallel, Phase 4 Study. Diabetes & Metabolism Journal. | integrate |
| **982** | A randomized, controlled comparison of different intensive lipid-lowering therapies in Chinese patients with non-ST-elevation acute coronary syndrome (NSTE-ACS): Ezetimibe and rosuvastatin versus high-dose rosuvastatin. International Journal of Cardiology. | integrate |
| **983** | Effect of ezetimibe combined with conventional dose of Rosuvastatin on coronary atheromatous plaque. | integrate |
| **984** | Observation on the efficacy of ezetimibe combined with rosuvastatin in the treatment of diabetes mellitus complicated with hyperlipidemia. Modern Medicine and Health. | integrate |
| **985** | Combination Therapy of Rosuvastatin and Ezetimibe in Patients with High Cardiovascular Risk. | integrate |
| **986** | Effect of ezetimibe combined with rosuvastatin on the incidence of adverse cardiovascular events in STEMI patients. | integrate |

**Table2.Quality evaluation results of the literature**

| **Study** | **generation of randomized sequences (2 scores)** | **allocation concealment (2 scores)** | **blinding (2 scores)** | **withdrawal and loss of visits(1 score)** | **total** |
| --- | --- | --- | --- | --- | --- |
| **Bomlee2023** | 2 | 2 | 1 | 1 | 6 |
| **Choi2023** | 2 | 2 | 1 | 1 | 6 |
| **Du2021** | 2 | 10 | 0 | 3 |  |
| **Feng2019** | 2 | 0 | 1 | 0 | 3 |
| **Hong2018** | 2 | 0 | 1 | 1 | 4 |
| **Hyup lee2023** | 2 | 2 | 1 | 1 | 6 |
| **Joon lee2023** | 2 | 2 | 1 | 1 | 6 |
| **Kim2016** | 2 | 2 | 1 | 1 | 6 |
| **Kim2018** | 2 | 2 | 1 | 1 | 6 |
| **Kim2022** | 2 | 2 | 1 | 0 | 5 |
| **Kim2023** | 2 | 2 | 1 | 0 | 5 |
| **Lee2023** | 2 | 2 | 1 | 1 | 6 |
| **Li2020** | 2 | 1 | 1 | 1 | 5 |
| **Ma2015** | 2 | 0 | 1 | 0 | 3 |
| **Moon2023** | 2 | 1 | 0 | 1 | 4 |
| **Ran2017** | 2 | 1 | 1 | 1 | 5 |
| **Su2016** | 2 | 2 | 0 | 0 | 4 |
| **Wang2018** | 2 | 0 | 1 | 0 | 3 |
| **Xu2013** | 2 | 0 | 1 | 0 | 3 |
| **Yang2016** | 2 | 1 | 1 | 1 | 5 |
| **Zhang2018** | 2 | 0 | 0 | 1 | 3 |
