# Supplementary material for: Efficacy and safety of moderate-intensity rosuvastatin plus ezetimibe versus high-intensity rosuvastatin monotherapy in the treatment of composite cardiovascular events with hypercholesterolemia: A meta-analysis
Source: PLoS One. 2024 Nov 13;19(11):e0310696. doi: 10.1371/journal.pone.0310696 (PMC11559983; doi:10.1371/journal.pone.0310696)
Supplement: Attachment — Submitted filename: PLOS one Efficacy and safety of Rosuvastatin with ezetimibe .pdf [file pone.0310696.s005.pdf]

PLOS ONE

Efficacy and safety of Moderate-intensity Rosuvastatin Plus Ezetimibe versus High-intensity Rosuvastatin Monotherapy in the treatment of Composite Cardiovascular Events with hypercholesterolemia: A Meta-analysis

--Manuscript Draft--

**Manuscript Number:**	PONE-D-24-07811
**Article Type:**	Research Article
**Full Title:**	Efficacy and safety of Moderate-intensity Rosuvastatin Plus Ezetimibe versus High-intensity Rosuvastatin Monotherapy in the treatment of Composite Cardiovascular Events with hypercholesterolemia: A Meta-analysis
**Short Title:**	Rosuvastatin Plus Ezetimibe vs Rosuvastatin : A Meta-analysis
**Corresponding Author:**	Lingyan Liu liu<br>The 926th Hospital of Joint Logistics Support Force of Chinese People's Liberation Army<br>Yunnan, CHINA
**Keywords:**	Keywords: Rosuvastatin, Ezetimibe, Moderate-intensity statin, high-intensity statin, randomized controlled trials
**Abstract:**	Abstract<br>Background :The gold standard in the treatment of dyslipidemia, statins significantly reduce the risk of cardiovascular disease.<br>Objective : To systematically review the efficacy and safety of Moderate-intensity Rosuvastatin Plus Ezetimibe compared with High-intensity Rosuvastatin in the treatment of Composite Cardiovascular Events.<br>Methods: PubMed, Embase, Cochrane Library, CINAHL, Web of Science, China Knowledge Network, China Biological Literature Database, Wan fang Database, and Weipu Database were searched to retrieve randomized controlled trials assessing the safety and efficacy of the two therapies from the time of construction to December 2023. The Jadad scale assessment tool was used to evaluate the quality of the included literatures, and Review Manager 5.4 software was used for meta-analysis. Heterogeneity of outcomes was estimated by the I2 test, where we applied risk ratios (RR) and 95% confidence intervals (CI) to assess dichotomous outcomes, and mean difference (MD) and 95% CI to present continuous outcomes. We used funnel plots to assess study publication bias and sensitivity analysis was used to address significant clinical heterogeneity.<br>Results: The meta-analysis described 21 RCTs involving 24592 participants, The findings indicated that combination therapy improved low-density lipoprotein cholesterol (LDL-C) (MD -8.06, 95% CI [-9.48, -6.64] $p < 0.05$), total cholesterol (TG) (MD -5.66, 95% CI [-8.51, -2.82] $p < 0.05$), and non-high-density lipoprotein cholesterol (non-HDL-C) (MD -17.04, 95% CI [-29.55, -4.54] $p < 0.05$) to a greater extent and superior in achieving LDL-C <70 (RR1.26, 95% CI [1.22, 1.29] $p < 0.05$) and LDL-C <55 (RR1.66, 95% CI [1.56, 1.77] $p < 0.05$) ratios and in the incidence of adverse events than the high-intensity Rosuvastatin monotherapy group. However, there was no statistical difference between the two in improving HDL-C, total cholesterol (TC) and preventing long-term composite adverse cardiovascular events (ACE). Publication bias was indicated by funnel plots. Sensitivity analysis suggested instability in long-term composite cardiovascular events., HDL-C, and TC results.<br>Conclusions: Moderate intensity statin plus ezetimibe with combination therapy had better efficacy and safety than high-intensity statins. Future validation is needed with more long-term high-quality large samples.
**Order of Authors:**	Lingyan Liu liu
	Xingbiao Yang
	Zhaoheng Yin
	Yongkun Deng
	Lai Yong

Powered by Editorial Manager® and ProduXion Manager® from Aries Systems Corporation

Additional Information:	
**Question**	**Response**
**Financial Disclosure**<br><br>Enter a financial disclosure statement that describes the sources of funding for the work included in this submission. Review the submission guidelines for detailed requirements. View published research articles from *PLOS ONE* for specific examples.<br><br>This statement is required for submission and **will appear in the published article** if the submission is accepted. Please make sure it is accurate.<br><br>**Funded studies**<br>Enter a statement with the following details:<br>• Initials of the authors who received each award<br>• Grant numbers awarded to each author<br>• The full name of each funder<br>• URL of each funder website<br>• Did the sponsors or funders play any role in the study design, data collection and analysis, decision to publish, or preparation of the manuscript?<br><br>Did you receive funding for this work?	The author(s) received no specific funding for this work.
**Competing Interests**<br><br>Use the instructions below to enter a competing interest statement for this submission. On behalf of all authors, disclose any competing interests that could be perceived to bias this work—acknowledging all financial support and any other relevant financial or non-financial competing interests.<br><br>This statement is required for submission and **will appear in the published article** if the submission is accepted. Please make sure it is accurate and that any funding sources listed in your Funding Information	The authors have declared that no competing interests exist.

later in the submission form are also declared in your Financial Disclosure statement.<br><br>View published research articles from *PLOS ONE* for specific examples.<br><br>**NO authors have competing interests**<br><br>Enter: *The authors have declared that no competing interests exist.*<br><br>**Authors with competing interests**<br><br>Enter competing interest details beginning with this statement:<br><br>*I have read the journal's policy and the authors of this manuscript have the following competing interests: [insert competing interests here]*<br><br>* typeset	
**Ethics Statement**<br><br>Enter an ethics statement for this submission. This statement is required if the study involved:<br><br>• Human participants<br>• Human specimens or tissue<br>• Vertebrate animals or cephalopods<br>• Vertebrate embryos or tissues<br>• Field research<br><br>Write "N/A" if the submission does not require an ethics statement.<br><br>General guidance is provided below. Consult the submission guidelines for detailed instructions. **Make sure that all information entered here is included in the Methods section of the manuscript.**	N/A

**Format for specific study types**<br><br>**Human Subject Research (involving human participants and/or tissue)**<br>• Give the name of the institutional review board or ethics committee that approved the study<br>• Include the approval number and/or a statement indicating approval of this research<br>• Indicate the form of consent obtained (written/oral) or the reason that consent was not obtained (e.g. the data were analyzed anonymously)<br><br>**Animal Research (involving vertebrate animals, embryos or tissues)**<br>• Provide the name of the Institutional Animal Care and Use Committee (IACUC) or other relevant ethics board that reviewed the study protocol, and indicate whether they approved this research or granted a formal waiver of ethical approval<br>• Include an approval number if one was obtained<br>• If the study involved *non-human primates*, add *additional details* about animal welfare and steps taken to ameliorate suffering<br>• If anesthesia, euthanasia, or any kind of animal sacrifice is part of the study, include briefly which substances and/or methods were applied<br><br>**Field Research**<br><br>Include the following details if this study involves the collection of plant, animal, or other materials from a natural setting:<br>• Field permit number<br>• Name of the institution or relevant body that granted permission	
**Data Availability**<br><br>Authors are required to make all data underlying the findings described fully available, without restriction, and from the time of publication. PLOS allows rare exceptions to address legal and ethical concerns. See the PLOS Data Policy and FAQ for detailed information.	Yes - all data are fully available without restriction

Powered by Editorial Manager® and ProduXion Manager® from Aries Systems Corporation

A Data Availability Statement describing where the data can be found is required at submission. Your answers to this question constitute the Data Availability Statement and **will be published in the article**, if accepted.<br><br>**Important:** Stating 'data available on request from the author' is not sufficient. If your data are only available upon request, select 'No' for the first question and explain your exceptional situation in the text box.<br><br>Do the authors confirm that all data underlying the findings described in their manuscript are fully available without restriction?	
**Describe where the data may be found in full sentences. If you are copying our sample text, replace any instances of XXX with the appropriate details.**<br><br>• If the data are **held or will be held in a public repository**, include URLs, accession numbers or DOIs. If this information will only be available after acceptance, indicate this by ticking the box below. For example: *All XXX files are available from the XXX database (accession number(s) XXX, XXX.).*<br>• If the data are all contained **within the manuscript and/or Supporting Information files**, enter the following: *All relevant data are within the manuscript and its Supporting Information files.*<br>• If neither of these applies but you are able to provide **details of access elsewhere**, with or without limitations, please do so. For example:<br><br>*Data cannot be shared publicly because of [XXX]. Data are available from the XXX Institutional Data Access / Ethics Committee (contact via XXX) for researchers who meet the criteria for access to confidential data.*<br><br>*The data underlying the results presented in the study are available from (include the name of the third party*	All relevant data are within the manuscript and its Supporting Information files.

and contact information or URL).<br>• This text is appropriate if the data are owned by a third party and authors do not have permission to share the data.<br><br>* typeset	
Additional data availability information:	

Click here to access/download;Manuscript;manuscript .docx

Title page

Full title:Efficacy and safety of Moderate-intensity Rosuvastatin Plus Ezetimibe versus High-intensity Rosuvastatin Monotherapy in the treatment of Composite Cardiovascular Events with hypercholesterolemia: A Meta-analysis

Short title:Rosuvastatin Plus Ezetimibe vs Rosuvastatin : A Meta-analysis

Lingyan Liu[1],Xingbiao Yang[1], Zhaoheng Yin[1], Yongkun Deng[1*] ,Yong Lai[2,3,4*]

1.Department of Pharmacy, The 926th Hospital of Joint Logistics Support Force of Chinese People's Liberation Army, Kaiyuan, Yunan, China

2.Yunnan Provincial Key Laboratory of Entomological Biopharmaceutical R&D, Dali University, Dali, Yunnan Province, China

3.National-Local Joint Engineering Research Center of Entomoceutics, Dali University, Dali , Yunnan Province, China

4.College of Pharmacy Dali University, Dali , Yunnan , China

*745763076@qq.com;*laiyong8879@163.com

Abstract

Background: The gold standard in the treatment of dyslipidemia, statins significantly reduce the risk of cardiovascular disease.

Objective: To systematically review the efficacy and safety of Moderate-intensity Rosuvastatin Plus Ezetimibe compared with High-intensity Rosuvastatin in the treatment of Composite Cardiovascular Events.

Methods: PubMed, Embase, Cochrane Library, CINAHL, Web of Science, China Knowledge Network, China Biological Literature Database, Wan fang Database, and Weipu Database were searched to retrieve randomized controlled trials assessing the safety and efficacy of the two therapies from the time of construction to December 2023. The Jadad scale assessment tool was used to evaluate the quality of the included literatures, and Review Manager 5.4 software was used for meta-analysis. Heterogeneity of outcomes was estimated by the I2 test, where we applied risk ratios (RR) and 95% confidence intervals (CI) to assess dichotomous outcomes, and mean difference (MD) and 95% CI to present continuous outcomes. We used funnel plots to assess study publication bias and sensitivity analysis was used to address significant clinical heterogeneity.

Results: The meta-analysis described 21 RCTs involving 24592 participants, The findings indicated that combination therapy improved low-density lipoprotein cholesterol (LDL-C) (MD -8.06, 95% CI [-9.48, -6.64] $p < 0.05$), total cholesterol (TG) (MD -5.66, 95% CI [-8.51, -2.82] $p < 0.05$), and non-high-density lipoprotein cholesterol (non-HDL-C) (MD -17.04, 95% CI [-29.55, -4.54] $p < 0.05$) to a greater

extent and superior in achieving LDL-C <70 (RR1.26, 95% CI [1.22, 1.29] $p < 0.05$) and LDL-C <55 (RR1.66, 95% CI [1.56, 1.77] $p < 0.05$) ratios and in the incidence of adverse events than the high-intensity Rosuvastatin monotherapy group. However, there was no statistical difference between the two in improving HDL-C, total cholesterol (TC) and preventing long-term composite adverse cardiovascular events (ACE). Publication bias was indicated by funnel plots. Sensitivity analysis suggested instability in long-term composite cardiovascular events., HDL-C, and TC results.

Conclusions: Moderate intensity statin plus ezetimibe with combination therapy had better efficacy and safety than high-intensity statins. Future validation is needed with more long-term high-quality large samples.

Keywords: Rosuvastatin, Ezetimibe, Moderate-intensity statin, high-intensity statin, randomized controlled trials

Introduction

Cardiovascular disease (CVD) is the primary global cause of death and disability and various preventive measures are being pursued to reduce the risk to patients. A primary preventive approach is to control cholesterol, especially LDL-C, to reduce the burden of atherosclerotic plaque, thereby decreasing the likelihood of future cardiac complications. Statins are easy to administer, have few drug interactions, have a favorable safety profile, and are considered the cornerstone of control of dyslipidemia or atherosclerotic cardiovascular disease (ASCVD)[1-3]. Several statins are available, which rosuvastatin is one of the most effective drugs for reducing CV risk.

Unfortunately, muscle symptoms associated with statins have been reported commonly and are a major reason for discontinuing treatment. Moreover, doubling the statin dose resulted in only a 6% increase in LDL-C reduction, but statin-related rhabdomyolysis, hepatic and renal damage, and other adverse effects are also aggravated [4].

In order to reduce the dosage of statin and improve the safety during treatment, more studies at both domestic and international have reported the combination of statin and ezetimibe for lipid adjustment in ASCVD patients, and international guidelines have recommended ezetimibe as a second-line choice for patients who are intolerant to statins or unable to achieve the target LDL-c level [5]. Ezetimibe is a Niemann-Pick C1-Like 1 inhibitor that resulting in about 15-20% reduction in LDL-C and .an increase in HDL-C of about 3%, with no effect on TG. In addition, combination therapy with statins resulted in an extra 21%-27% reduction in LDL-C levels [6, 7].

Recently, Various randomized controlled trials have shown differences in safety and efficacy between moderate-intensity rosuvastatin combined with ezetimibe and high-intensity rosuvastatin, but there are no consistent conclusions. Kim et al. [8] concluded that Rosuvastatin combination with ezetimibe was more effective, but Choi et al. [9] reported that the advantage was not obvious. Whether moderate-intensity Rosuvastatin combined with ezetimibe has advantages in ACE prevention and lipid regulation needs to be further explored. In this study, we selected the RCTS of medium-intensity Rosuvastatin plus ezetimibe versus double-dose Rosuvastatin for the treatment of CVD patients, and performed meta-analysis on the efficacy and safety in the two groups, in order to provide references for the clinical use of drugs.

Materials and methods

Data sources and search strategy

We conducted a systematic search of PubMed, Embase, Cochrane Library, CINAHL, Web of Science, China Knowledge Network, China Biological Literature Database, Wan fang Database, and Weipu Database from inception to December 2023. The main search keywords were combinations of “Rosuvastatin”, “Ezetimibe”, “Moderate-intensity statin”, “high-intensity statin” and “randomized controlled trials” in various databases. Furthermore, we manually searched bibliography sections from previously published relevant research to ascertain potential studies.

Study selection

The search results underwent a title, abstract and full text sieve by two reviewers conducted independently, and the differences were resolved by a third author. Inclusion criteria for this meta-analysis included:(1) age:18-80years;(2) a randomized clinical trial; (3) rosuvastatin 10mg plus ezetimibe vs rosuvastatin 20mg;(4) Included studies had to report at least one clinical event among outcomes of interest;(5) no language restrictions

Data extraction and quality evaluation

Data extraction was independently performed by two reviewers. The extracted information included the first author, year of publication, country, disease status,

sample size, interventions, treatment duration, gender, age, and clinical outcomes. A Jadad scale was used to evaluate the quality of the study, which included the generation of randomized sequences (2 scores), allocation concealment (2 scores), blinding (2 scores), and withdrawal and loss of visits (1 score), with 0-2 scores for each of these items according to the criteria. while the total was 7 scores, 1-3 scores were regarded as low-quality studies, and 4-7 scores were regarded as high-quality studies.

Endpoints

Regarding long-term composite ACE, we investigated the primary endpoint (composite of cardiovascular death, major cardiovascular events, or nonfatal stroke), the Secondary efficacy endpoint (composite of all-cause death, major cardiovascular events, or nonfatal stroke), and individual clinical endpoints (all cardiovascular events) in three areas.

Regarding clinical efficacy and safety endpoints, the lipid profile included changes from baseline in LDL-C, HDL-C, non-HDL-C, TG, and TC, as well as the proportion of patients with an LDL-C level of less than 70 or 55 mg/dL; Safety endpoints included the occurrence of overall adverse events (Muscle-related adverse events, discontinuation or dose reduction of study drug due to intolerance events or new-onset disease).

Statistical analysis

All data analysis were performed using RevMan 5.4. We use the risk ratio (RR)

and 95% confidence intervals (CI) to evaluate dichotomous outcomes and the mean difference (MD) and 95% CI was used to present continuous outcomes. When original studies do not report the standard deviation of differences for continuous variables, use the Cochrane Handbook to convert the values. The heterogeneity of the results was evaluated by I2 values using $\chi 2$ test. When heterogeneity showed no significant differences ($P > 0.10$, $I2 < 50\%$) with a fixed effect model, otherwise, the random effects model was employed. There were significant differences between the two groups ($p < 0.05$). We used sensitivity analysis to address the significant clinical heterogeneity. Funnel plots were utilized to assess the research for publication bias when it included more than ten studies.

RESULTS

Search Results

In the target databases, initially turned up 986 pieces of literature for the study, among which 405 were excluded due to duplication, following a review of the titles and abstracts, 240 were eliminated and 280 full-text papers were rejected according to the inclusion and exclusion criterion. Ultimately, the remaining 21 studies (25 data sets) were included in the meta-analysis,13 articles in English and 8 articles in Chinese(Fig 1).

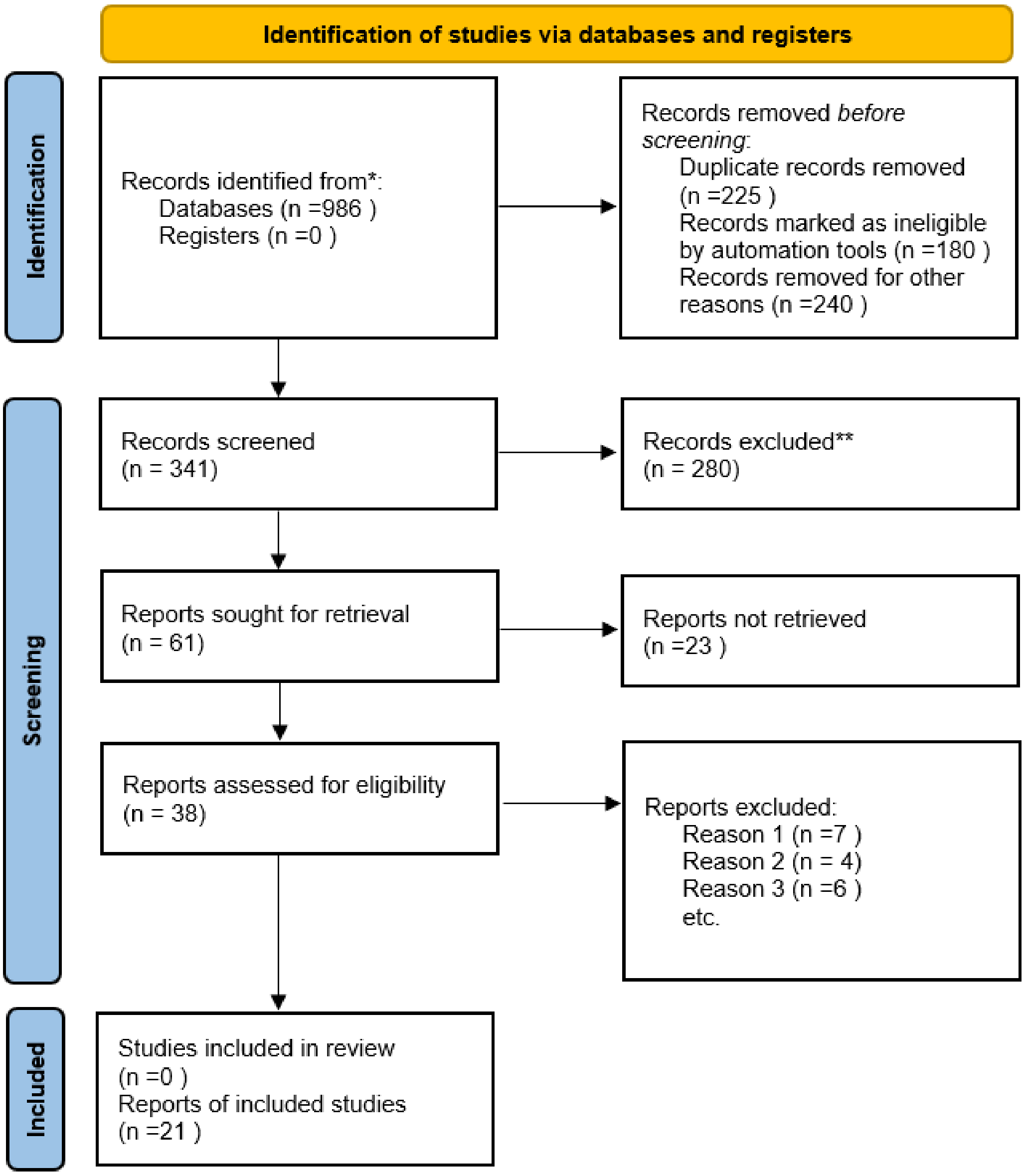

Fig 1. PRISMA Flowchart for selection of relevant studies

Study Characteristics and Quality Assessment

The meta-analysis presented 21 RCTS with a total of 24592 participants conducted from 2016 to 2023. Patients with various cardiovascular disease statuses were included, such as those with hyperlipidemia alone or in combination with diabetes mellitus (DM), ASCVD or combined with DM, acute coronary syndrome (ACS),coronary artery disease, Large-artery atherosclerosis(LAA), cerebral infarction, ST-segment elevation

myocardial infarction (STEMI).The interventions were ezetimibe 10 mg plus rosuvastatin 10 mg against rosuvastatin 20 mg, twelve of the included trials were published in Korea, eleven in China, and the treatment ranged in length from 8 weeks to 3 years. According to the Jadad system, 6 studies were categorized as low-quality studies and the other 15 studies were categorized as high-quality studies (Table 1).

Table 1. meta-analysis of the general characteristics of the studies included.

Author year	Country	Trial Duration	Patient Comparison	R10+EZ10(n)	R20(n)	Age (years)		Male (%)		outcome indicator	Jadad score
						R10+EZ10	R20	R10+EZ10	R20		
Bomlee 2023[10]	Korea	3-year	ASCVD	1894	1886	63.98 ±10.12	64.58±10.24	1420 (75)	1406 (74.5)	①⑦⑧	6
Choi2023 [9]	Korea	Week24	ASCVD	126	132	58.93±26.99	53.66±36.73	91 (72.2)	104 (78.8)	②⑧	6
Du2021 [11]	China	1-year	ASCVD	35	35	56.54±6.49	57.03±6.46	20 (57.14)	19 (54.29)	②	3
Feng2019 [12]	China	Week24	CAD	35	34	59±9	61±8	28 (80)	24 (71)	②③④⑤⑧	3
Hong2018 [13]	Korea	Week 8	Hypercholesterolemia	66	64	62.5 ±8.9	64.2±8.3	39 (59.1)	40 (62.5)	②⑧	4
Hyup lee2023(1) [14]	Korea	3-year	ASCVD	273	301	77 ±2	77±2	173 (63.4)	180 (59.8)	①②⑧	6
Hyup lee2023(2) [14]	Korea	3-year	ASCVD	1621	1585	61 ± 8	62 ±8	1247 (76.9)	1226 (77.4)	①②⑧	6
Joon lee2023(1) [15]	Korea	3-year	ASCVD+DM	701	697	64±9	65±9	545 (77.7)	515 (73.9)	①②③④⑤⑦⑧	6
Joonlee2023(2)[15]	Korea	3-year	ASCVD	1193	1189	63±10	63±10	875	891 (74.9)	①②③④⑤⑦⑧	6

								(73.3)			
Kim2016 [16]	Korea	Week 8	Hypercholesterolemia	203	204	64.2±7.9	64.3±9.3	113 (55.7)	118 (57.8)	②③④⑤⑥	6
Kim2018[17]	Korea	Week 8	Hypercholesterolemia	60	63	61.77±9.92	59.33±9.13	31 (51.7)	39 (61.9)	②③④⑤⑥⑧	6
Kim2022 [8]	Korea	3-year	ASCVD	1894	1886	64±10	64±10	1420 (75)	1406 (75)	①②⑦⑧	5
Kim2023 (1)[18]	Korea	3-year	ASCVD	474	480	67.1±8.4	67.8±8.5	Female		①②③④⑤⑦⑧	5
Kim2023 (2)[18]	Korea	3-year	ASCVD	1420	1406	62.4±9.6	62.8±9.7	Male		①②③④⑤⑦⑧	5
Lee2023(1) [19]	Korea	3-year	ASCVD	757	754	63.6 ±9.9	64.3±10.3	616 (81.4)	600 (79.6)	①②⑦⑧	6
Lee2023(2)[19]	Korea	3-year	ASCVD	1137	1132	63.5 ±9.3	63.9 ±9.2	804 (70.7)	806 (71.2)	①②⑦⑧	6
Li2020[20]	China	Week12	LAA	92	92	73.4±6.25	71.0±3.62	47 (51.09)	49 (53.27)	⑧	5
Ma2015 [21]	China	Week16	Hypercholesterolemia	40	40	63. 5±9. 6	62. 8±9. 9	NA	NA	②③④⑤	3
Moon(22) 2023	Korea	Week24	ASCVD + DM	48	51	61.88±6.47	61.16±7.09	28 (58.33)	37 (72.55)	②⑧	4
Ran2017 [23]	China	Week 12	ACS	42	41	60.4 ±8.2	60.5±10.0	32 (76.2)	30 (73.2)	②③④⑤⑥⑧	5
Su2016 [24]	China	Week 12	CAD	48	48	53.18±4.32	53.2±4.09	30 (62.5)	31 (64.58)	②③④⑤	4
Wang2018 [25]	China	Week12	Hypercholesterolemia+DM	26	26	57.3±10.4		NA	NA	②③④⑤⑧	3
Xu2013 [24]	China	Week16	cerebral infarction	29	27	60±11	60±0.9	16 (55.2)	13 (48.1)	②③④⑤	3

Yang2016 [26]	Korea	Week 12	CAD	38	39	62.1 ±9.5	62.7 ±9.6	24 (63.2)	26 (66.7)	②③④⑤	5
Zhang2018[27]	China	1-year	STEMI	78	50	61.01±7. 57	60.9±8. 36	56 (71. 8)	34 (68)	⑧	3

Note: R:Rosuvastatin;EZ:ezetimibe;ASCVD:atherosclerotic cardiovascular disease; CAD: coronary artery disease; DM: diabetes mellitus ; LAA: Large-artery atherosclerosis; ACS: acute coronary syndrome; STEMI: ST-segmen televation myocardial infarction ; ①long-term composite ACE②LDL-C③HDL-C ④TC ⑤TG ⑥non-HDL-C ⑦the proportion of patients whose LDL-C levels were below 70 or 55 mg/dL ⑧the occurrence of adverse events

Meta-analysis of long-term composite cardiovascular events

Six RCTs (10 data sets) reported Primary endpoint, five RCTs (8data sets) reported Secondary efficacy endpoint, four RCTs (6data sets) reported Individual clinical endpoint. We used a randomized controlled model for analysis and discovered that combination therapy was comparable to rosuvastatin monotherapy in preventing Primary endpoint(RR 0.93,95% CI: [0.86, 1.01] p=0.08), Secondary efficacy endpoint (RR 0.94,95% CI: [0.86, 1.02] p=0.16)and Individual clinical endpoint(RR 1.01,95% CI: [0.88, 1.16] p=0.87), with no difference of statistical significance(Fig 2).However, due to the large heterogeneity of Individual clinical endpoint, a sensitivity analysis is required.

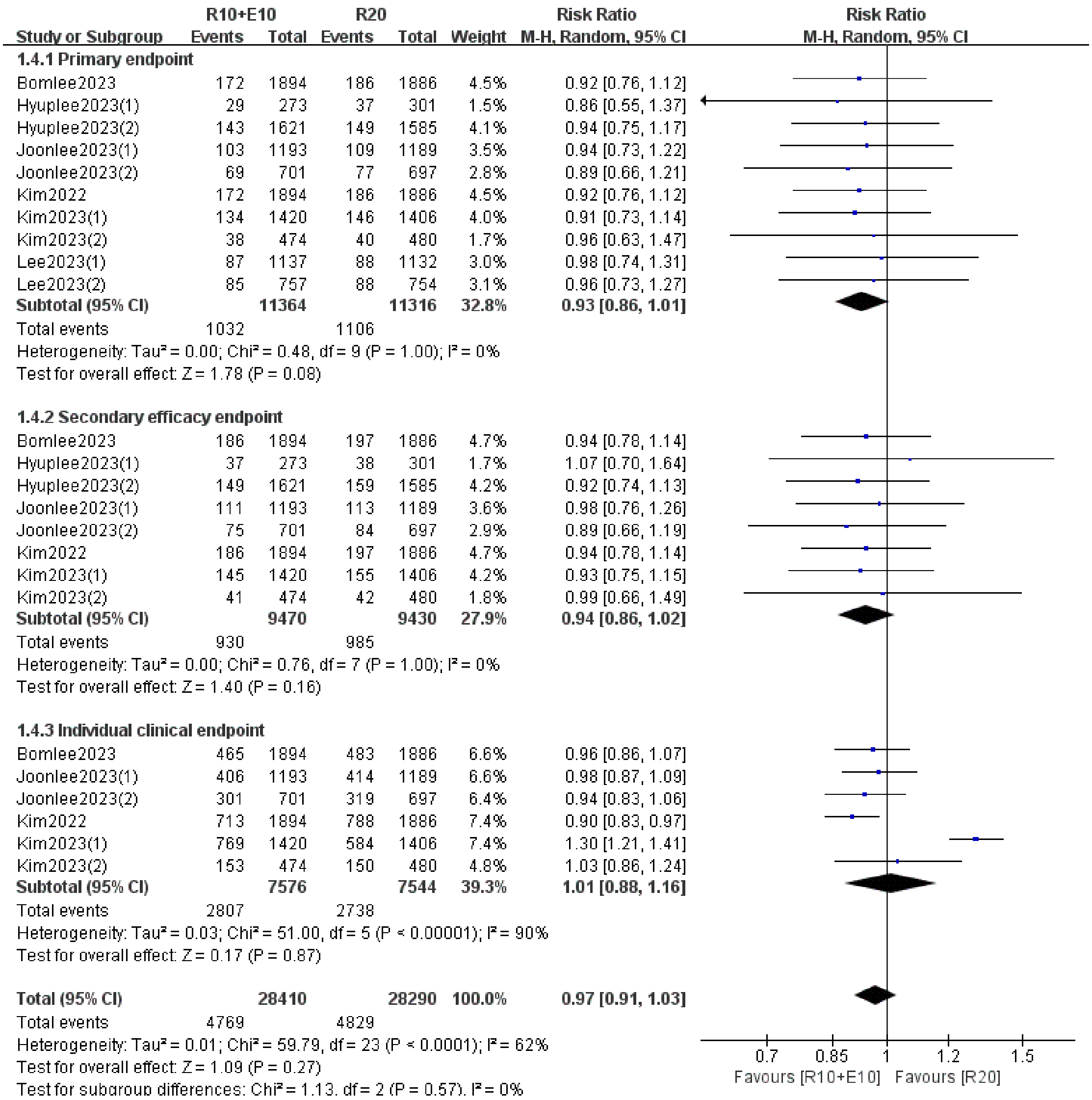

Fig 2. Forest plot of long-term composite cardiovascular events.

Meta-analysis of lipid profile

For changes in LDL-C, we included 18studies (22 data sets) and there was a large heterogeneity among studies (I2= 61%, P < 0.01) and randomized model was used for the analysis. Meta-analysis showed that the combination group improved LDC-C better than the monotherapy group, and the difference was statistically significant (MD -8.06, 95% CI [-9.48, -6.64] p < 0.05) (Fig 3).

A total of 11 studies(13 data sets) reported changes in HDL-C,TC,TG, The results

showed that moderate-intensity statin plus ezetimibe produced significantly superior reduction than high-intensity statin alone in the TG level (MD -5.66, 95% CI [-8.51, -2.82] $p < 0.05$), but no significant difference for the effects of treatments on HDL-C(MD 0.24, 95% CI [-1.37, 1.84] $p = 0.77$) or TC level (MD -13.12 [-27.20, 0.96] $p = 0.07$) (Fig 3).

Three studies reported non-HDL-C, which we analyzed using a random-effects model, and the results showed that the combination group improved non-HDL-C more than the monotherapy (MD -7.27, 95% CI [-9.80, -4.74] $p < 0.05$) accompanied by a high degree of heterogeneity (I2 = 97%, $P < 0.01$), and the difference was statistically significant (Fig 3).

	R10+E10			R20				Mean Difference
Study or Subgroup	Mean	SD	Total	Mean	SD	Total	Weight	IV, Random, 95% CI
**1.2.1 LDL-C**								
Choi2023	-23.8	31.4	126	-11.4	28.6	132	1.5%	-12.40 [-19.74, -5.06]
Du2021	-59.1	25.65	203	-49.4	27.14	204	1.6%	-9.70 [-14.83, -4.57]
Feng2019	-55.8	16.39	60	-47.96	17.77	63	1.6%	-7.84 [-13.88, -1.80]
Hong2018	-57.1	18.5	66	-49.2	27.1	64	1.5%	-7.90 [-15.90, 0.10]
Hyuplee2023(1)	-22.35	22.4	273	-16.65	24.22	301	1.7%	-5.70 [-9.51, -1.89]
Hyuplee2023(2)	-23.05	23.43	1621	-13.7	23.57	1585	1.8%	-9.35 [-10.98, -7.72]
Joonlee2023(1)	-50.9	20.42	51	-57.08	22.35	48	1.5%	6.18 [-2.27, 14.63]
Joonlee2023(2)	-95	23.64	42	-77	30.41	41	1.3%	-18.00 [-29.74, -6.26]
Kim2016	-57.6	17.4	38	-56	17.2	39	1.5%	-1.60 [-9.33, 6.13]
Kim2018	-30	29.14	339	-21.7	30.71	335	1.7%	-8.30 [-12.82, -3.78]
Kim2022	-25.3	27.43	1010	-18	28.31	979	1.7%	-7.30 [-9.75, -4.85]
Kim2023(1)	-30.8	24.47	34	-22.9	26.28	35	1.3%	-7.90 [-19.88, 4.08]
Kim2023(2)	-47.7	7.13	40	-36.36	7.77	40	1.7%	-11.34 [-14.61, -8.07]
Lee2023(1)	-30.24	14.67	35	-24.3	14.34	35	1.6%	-5.94 [-12.74, 0.86]
Lee2023(2)	-29.88	13.58	48	-13.14	13.56	48	1.6%	-16.74 [-22.17, -11.31]
Ma2015	-16.38	8.04	29	-16.74	8.49	27	1.7%	0.36 [-3.98, 4.70]
Moon2023	-39.6	19.33	26	-34.74	18.1	26	1.4%	-4.86 [-15.04, 5.32]
Ran2017	-21.4	24.77	497	-13.75	23.33	476	1.7%	-7.65 [-10.67, -4.63]
Su2016	-23.7	24.98	852	-15.4	25.98	839	1.7%	-8.30 [-10.73, -5.87]
Wang2018	-22.7	24.91	1349	-14.7	25.73	1315	1.7%	-8.00 [-9.92, -6.08]
Xu2013	-18.8	32.2	530	-9.7	34.5	536	1.7%	-9.10 [-13.11, -5.09]
Yang2016	-23.5	29.4	819	-12.6	31.9	779	1.7%	-10.90 [-13.91, -7.89]
**Subtotal (95% CI)**			**8088**			**7947**	**35.2%**	**-8.06 [-9.48, -6.64]**
Heterogeneity: Tau² = 5.60; Chi² = 54.41, df = 21 (P < 0.0001); I² = 61%								
Test for overall effect: Z = 11.11 (P < 0.00001)								
**1.2.2 HDL-C**								
Feng2019	3.6	9.4	34	4.6	9.01	35	1.7%	-1.00 [-5.35, 3.35]
Joonlee2023(1)	-1.35	11.59	852	-0.35	11.51	839	1.8%	-1.00 [-2.10, 0.10]
Joonlee2023(2)	-1	10.29	497	0.7	10.61	476	1.8%	-1.70 [-3.01, -0.39]
Kim2016	14.1	28.5	203	11.7	29.99	204	1.6%	2.40 [-3.28, 8.08]
Kim2018	12.47	25.6	60	13.9	20.25	63	1.5%	-1.43 [-9.61, 6.75]
Kim2023(1)	-1	10.4	1010	0.7	11.24	979	1.8%	-1.70 [-2.65, -0.75]
Kim2023(2)	-0.3	12.86	339	-0.05	12.57	335	1.7%	-0.25 [-2.17, 1.67]
Ma2015	18.9	3.43	40	12.6	3.42	40	1.8%	6.30 [4.80, 7.80]
Ran2017	2	6	42	2	7	41	1.7%	0.00 [-2.81, 2.81]
Su2016	1.8	5.51	48	1.62	5.67	48	1.7%	0.18 [-2.06, 2.42]
Wang2018	0.9	4.08	26	0.72	4.42	26	1.7%	0.18 [-2.13, 2.49]
Xu2013	1.44	4.26	29	0.54	6.92	27	1.7%	0.90 [-2.14, 3.94]
Yang2016	5.1	17.7	38	5.9	18.4	39	1.5%	-0.80 [-8.86, 7.26]
**Subtotal (95% CI)**			**3218**			**3152**	**22.0%**	**0.24 [-1.37, 1.84]**
Heterogeneity: Tau² = 6.18; Chi² = 90.38, df = 12 (P < 0.00001); I² = 87%								
Test for overall effect: Z = 0.29 (P = 0.77)								
**1.2.3 TC**								
Feng2019	-28.6	30.48	34	-24.4	32.48	35	1.1%	-4.20 [-19.06, 10.66]
Joonlee2023(1)	-24.7	29.59	852	-15.1	31.36	839	1.7%	-9.60 [-12.51, -6.69]
Joonlee2023(2)	-24.4	31.67	497	-12.95	29.23	476	1.7%	-11.45 [-15.28, -7.62]
Kim2016	-39.6	19.95	203	-32.9	20	204	1.7%	-6.70 [-10.58, -2.82]
Kim2018	-38.92	13.47	60	-32.45	14.88	63	1.7%	-6.47 [-11.48, -1.46]
Kim2023(1)	-22.7	29.77	1010	-13.05	31.89	979	1.7%	-9.65 [-12.36, -6.94]
Kim2023(2)	-27.75	30.99	339	-15.05	30.68	335	1.7%	-12.70 [-17.36, -8.04]
Ma2015	-45	8.62	40	37.8	8.2	40	1.7%	-82.80 [-86.49, -79.11]
Ran2017	-96	29.81	42	-81	40.63	41	1.1%	-15.00 [-30.36, 0.36]
Su2016	-56.34	20.68	48	-52.38	19.04	48	1.5%	-3.96 [-11.91, 3.99]
Wang2018	-53.82	21.85	26	-46.62	21.18	26	1.3%	-7.20 [-18.90, 4.50]
Xu2013	-18	11.26	29	-18.36	10.44	27	1.6%	0.36 [-5.32, 6.04]
Yang2016	-39.2	12.8	38	-39.2	13.2	39	1.6%	0.00 [-5.81, 5.81]
**Subtotal (95% CI)**			**3218**			**3152**	**20.0%**	**-13.12 [-27.20, 0.96]**
Heterogeneity: Tau² = 654.81; Chi² = 1416.64, df = 12 (P < 0.00001); I² = 99%								
Test for overall effect: Z = 1.83 (P = 0.07)								
**1.2.4 TG**								
Feng2019	-15.1	59.83	34	-10.9	64.95	35	0.5%	-4.20 [-33.65, 25.25]
Joonlee2023(1)	-12.35	55.94	852	-3.3	59.36	839	1.6%	-9.05 [-14.55, -3.55]
Joonlee2023(2)	-16.8	62.02	497	-3.89	64.41	476	1.5%	-12.91 [-20.86, -4.96]
Kim2016	-22.7	49.87	203	-13.4	49.99	204	1.4%	-9.30 [-19.00, 0.40]
Kim2018	-20.45	32.53	60	-19.36	32.64	63	1.3%	-1.09 [-12.61, 10.43]
Kim2023(1)	-11.8	58.72	1010	-3.55	61.17	979	1.6%	-8.25 [-13.52, -2.98]
Kim2023(2)	-18.4	56.33	339	0.46	55.87	335	1.5%	-18.86 [-27.33, -10.39]
Ma2015	-28.8	7.93	40	-24.3	7.29	40	1.7%	-4.50 [-7.84, -1.16]
Ran2017	-31	36.59	42	-27	38.3	41	1.0%	-4.00 [-20.12, 12.12]
Su2016	-8.28	10.19	48	-7.2	9.39	48	1.7%	-1.08 [-5.00, 2.84]
Wang2018	-21.06	8.49	26	-20.34	7.78	26	1.7%	-0.72 [-5.15, 3.71]
Xu2013	-2.88	7.15	29	-0.9	8.4	27	1.7%	-1.98 [-6.08, 2.12]
Yang2016	-24.6	30.43	38	-25.9	35.4	39	1.1%	1.30 [-13.43, 16.03]
**Subtotal (95% CI)**			**3218**			**3152**	**18.4%**	**-5.66 [-8.51, -2.82]**
Heterogeneity: Tau² = 13.08; Chi² = 28.94, df = 12 (P = 0.004); I² = 59%								
Test for overall effect: Z = 3.90 (P < 0.0001)								
**1.2.5 NHDL-C**								
Kim2016	-54.9	24.22	203	-45.8	24.28	204	1.7%	-9.10 [-13.81, -4.39]
Kim2018	-52.85	15.64	60	-45.04	17.3	63	1.6%	-7.81 [-13.63, -1.99]
Ran2017	-98	30	42	-56	38.2	41	1.1%	-42.00 [-56.80, -27.20]
**Subtotal (95% CI)**			**305**			**308**	**4.4%**	**-17.04 [-29.55, -4.54]**
Heterogeneity: Tau² = 102.18; Chi² = 18.56, df = 2 (P < 0.0001); I² = 89%								
Test for overall effect: Z = 2.67 (P = 0.008)								
**Total (95% CI)**			**18047**			**17711**	**100.0%**	**-7.27 [-9.80, -4.74]**
Heterogeneity: Tau² = 94.48; Chi² = 2374.26, df = 63 (P < 0.00001); I² = 97%								
Test for overall effect: Z = 5.63 (P < 0.00001)								
Test for subgroup differences: Chi² = 63.45, df = 4 (P < 0.00001), I² = 93.7%								

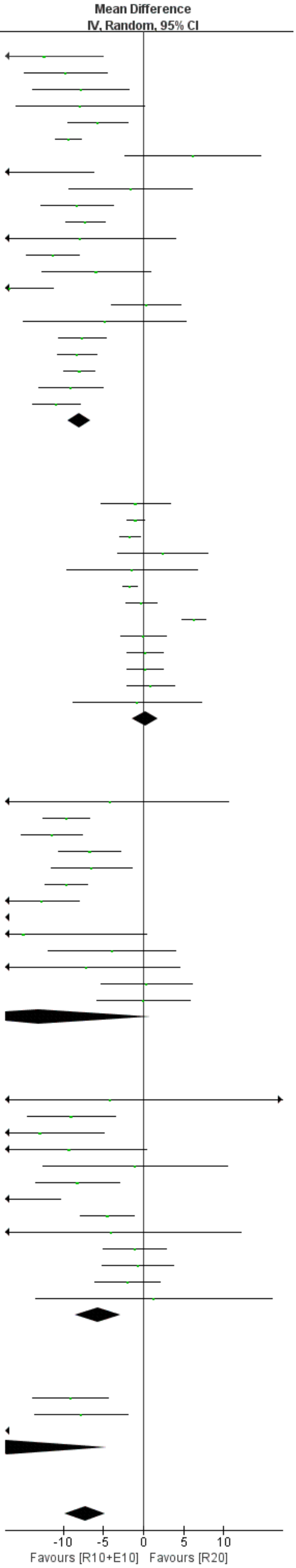

Fig 3. Forest plot of the results of the assessment of changes in lipid profile efficacy.

Meta-analysis of the proportion of patients whose LDL-C levels

Five studies (8 data sets) assessed the proportion of patients who achieved LDL-C levels <70 mg/dL, The results of the test for heterogeneity between studies were P = 0.43, I2=0%, so the fixed model was used for analysis. The results showed that the compliance rate was high in the combined group and the difference was significant (RR 1.26, 95% CI: [1.22, 1.29] p<0.05); similar results were observed in the proportion of patients who achieved 55mg/dL (RR 1.66, 95% CI: [1.56, 1.77] p<0.05) of three studies (5 data sets) included in the total (Fig 4).

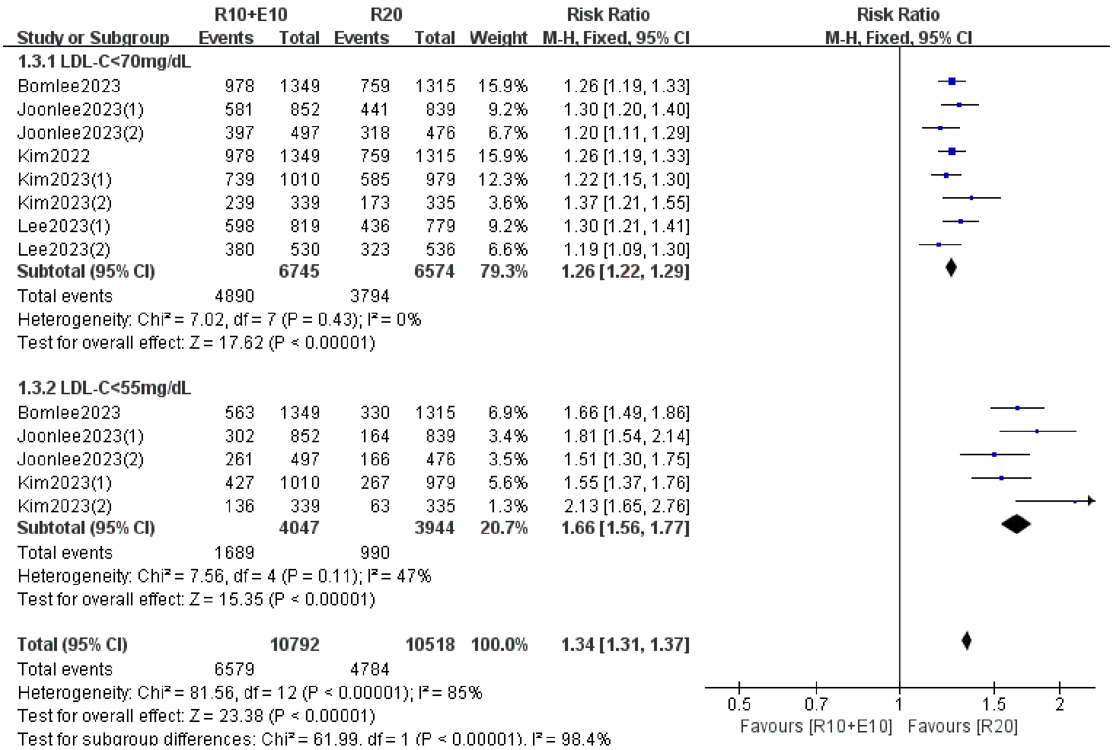

Fig 4. Forest plot of the proportion of patients who achieved LDL-C levels <70 mg/dL and 55mg/dL.

Meta-analysis of overall adverse events

Fourteen studies and 23200 participants were involved in all adverse event assessments and fixed model was used for the analysis with low heterogeneity among studies (I2= 43%, P=0.03), which showed that the rate of adverse events was lower in the combination group than in the high-dose group (RR 0.76, 95% CI: [0.71, 0.81] p<0.05) (Fig 5).

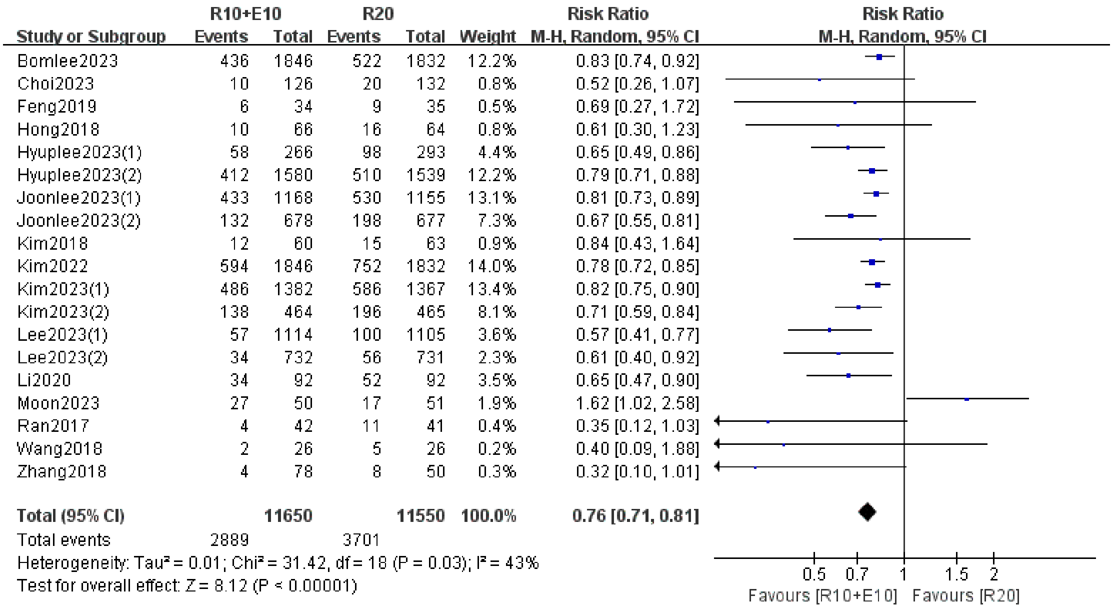

Fig 5. Forest plot of the results of the occurrence of adverse events

Sensitivity analysis

Sensitivity analysis was carried out by excluding individual studies in turn. After excluding literature with high heterogeneity, the LDL-C、TG、NON-HDL-C differences remained statistically significant, indicating that the analysis was relatively stable, but ACE、HDL-C、TC were altered significantly, suggesting that the analysis was unstable (Table 2).

Table.2 Test of heterogeneity Publication bias

outcome	No. of study	95%CI	P value	I^2(%)
ACE	Kim2023(2)[18]	[0.90, 0.97]	0.0009	0
LDL-C	Ma2015[21]	[-9.80, -7.31]	0.0001	48
HDL-C	Ma2015[21]	[-1.56, -0.49]	0.0002	0
TC	Ma2015[21]	[-9.83, -5.04]	0.0001	61
TG	Kim2023(2)[18]	[-6.84, -2.21]	0.0001	38
non-HDL-C	Ran2017[23]	[-12.25, -4.93]	0.0001	0

Publication bias

Five outcome indicators (LDL-C, HDL-C, TC, TG, adverse events) were included in ≥10 studies, and the risk of publication bias was assessed using an inverted funnel plot, which showed a symmetry between the left and right sides of the results, no publication bias and stable results. (Figs 6 and 7)

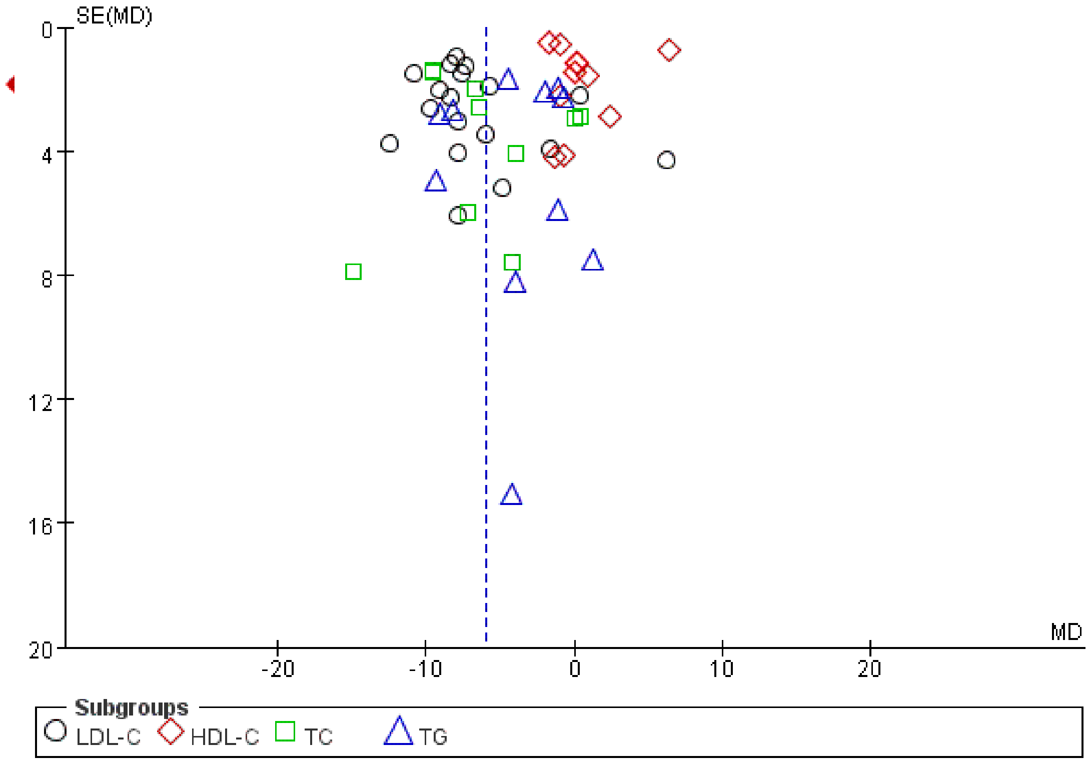

Fig 6. Funnel diagram of LDL-C, HDL-C, TC, TG

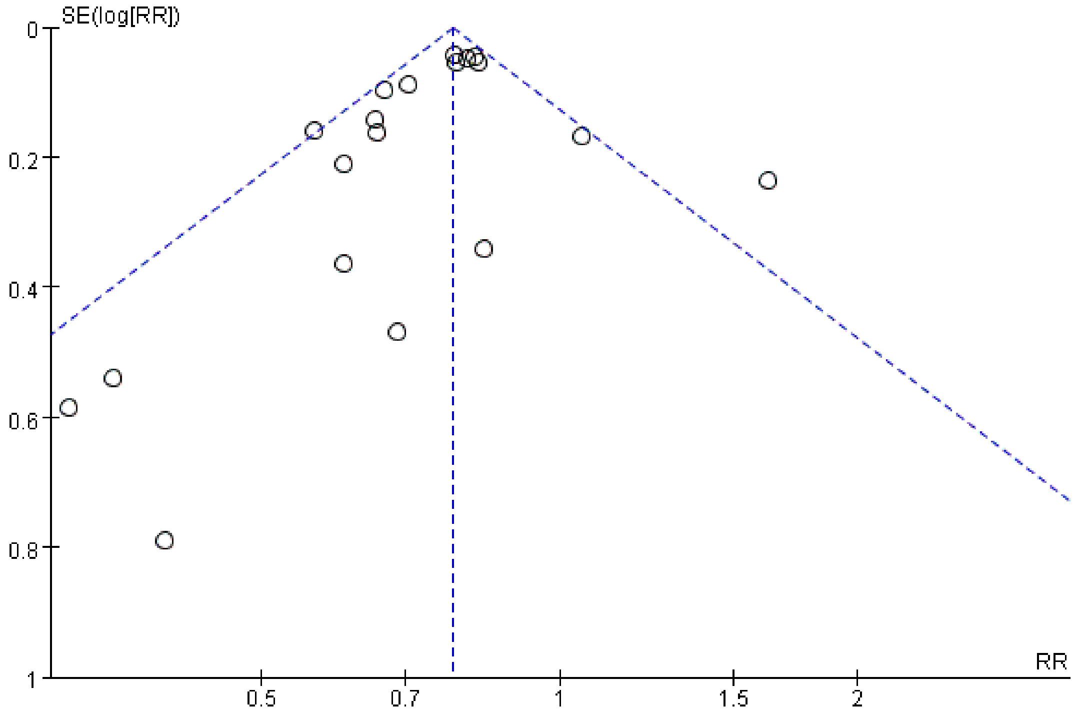

Fig 7. Funnel diagram of adverse events.

Discussion

The incidence of CVD is on the rise, imposing a huge burden on society and the economy, and lipid management cannot be delayed. Low control of dyslipidemia and twice as many adverse cardiovascular events as those with normal lipids can seriously affect the quality of patient survival [28, 29]. Common lipid markers used to assess CVD risk include TC, LDL-C, HDL-C, TG and non-HDL-C, and in most lipid-lowering intervention studies, LDL-C was the strongest independent predictor of the relationship between the effect of lipid lowering and the reduction of ASCVD risk. Therefore, most national or regional lipid management guidelines recommend LDL-Cas the primary goal of lipid-lowering therapy. The ESC/EAS guidelines for lipid management state that low-density lipoprotein cholesterol (LDL-C) levels should be controlled at less than 55 mg/dL and reduced by at least 50% for patients at very high and high risk of cardiovascular disease, respectively [5, 30].

Statins significantly reduce cardiovascular morbidity and mortality while lowering serum cholesterol [31]. Therefore, in order to regulate lipids up to standard, statins should be preferred clinically, but for patients who do not achieve LDL-C after conventional dose statin treatment, doubling the statin dose or combining with other lipid regulating drugs such as ezetimibe may be considered. Some studies have shown that statin combined with ezetimibe reduces LDL-C more significantly than doubling the statin dose, bringing LDL-C to the therapeutic target in the CVD population, but it has also been suggested that combining ezetimibe may not provide as much

cardiovascular benefit as doubling the dose [32, 33]. In this study, we compared the effects of combining ezetimibe with double-dose rosuvastatin on the risk of long-term composite ACE of the subgroup analyses that combination therapy was comparable to that of monotherapy, after removing the Kim [18] study for sensitivity analyses, the incidence of Individual clinical CVD was found to be lower in the combination therapy group than in the monotherapy group.

our analysis of lipid parameters and safety, combination therapy improved LDL-C, TG, non-HDL-C and reduced incidence of adverse reactions more than monotherapy. As well, combination therapy was superior proportion of patients to monotherapy in achieving LDL-C <70 and LDL-C <55. Furthermore, there was no statistical difference between the two in improving HDL-C, TC. we suggest that the addition of ezetimibe to rosuvastatin therapy is preferable to increasing the statin dose, and not only better lowering of LDL-C, but also reduce incidence of adverse reactions. Previous meta-analyses showed that for every 1 mmol/L reduction in LDL-C, ASCVD events were reduced by 20%~23% [34, 35]. The outcome successful to demonstrate a strong association between LDL-C and CVD risk and larger studies are needed in the future to confirm.

In addition, the beneficial effects of the combination therapy on TG and non-HDL-C were greater compared with high-intensity Rosuvastatin monotherapy, which we consider a noteworthy result. In recent years, the role of non-HDL-C in lipid management for the prevention of ASCVD has become increasingly important, it better anticipates ASCVD risk in patients with obesity, diabetes, and metabolic syndrome [36].

It has been demonstrated that non-HDL - C is a predictor of ASCVD risk better than LDL-C, with or without statin therapy. In a meta-analysis of statin studies, it was found that the magnitude of ASCVD reduction correlated better with the magnitude of non-HDL-C reduction than with the magnitude of LDL- C reduction [37, 38]. International guidelines recommend that non-HDL-C should as a co-primary therapeutic target of lipid-lowering therapy for CVD risk reduction, especially for patients at high risk of dyslipidemia [39-42]. NON-HDL-C may be an independent CVD risk factor in the future.

Epidemiological studies have shown that elevated TG levels is a risk factor for CVD, and previous study have shown that the association between TG and CVD risk is attenuated after adjustment for HDL-C and non-HDL-C, but it is still significant [43, 44]. High TG levels are associated with elevated cholesterol and low HLD-C levels, and the American Heart Association (AHA) / American College of Cardiology (ACC) cholesterol guidelines recommend elevated triglycerides as a "risk enhancer" for ASCVD [45]. Our meta-analysis is clinically relevant because we found differences in these lipid levels between the two treatments.

In the results of the heterogeneity test, the I2 values for LDL-C and TC were 48 and 61, respectively, indicating a moderate degree of heterogeneity (Table 2). Therefore, a reanalysis was performed using the random effects model. The same results of statistical significance for LDL-C indicated the presence of some heterogeneity, but the effect on the meta-analysis was not significant, in contrast to TC, which had a significant effect on the meta-analysis.

Our study has the following limitations. First, some of the study data were converted using formulas, and the data may not be accurate. Second, we included patients with different disease states, which may have contributed to the heterogeneity among this study, and third, we only included studies from China and Korea, and the results are only informative for Asian patients. Despite the limitations, our meta-analysis is meaningful because it provides clinical evidence for better pharmacological treatment of patients with dyslipidemia.

Conclusions

The study suggests that moderate-intensity Rosuvastatin in combination with ezetimibe can be an alternative to high-intensity statins with better efficacy and safety. Future validation is needed with more long-term high-quality large samples.

Funding Statement

The authors received no specific funding for this work.

Data Availability

All relevant data are within the paper and its Supporting Information files.

Author Contributions

Conceptualization: Lingyan Liu,Yong Lai,Yongkun Deng ,Zhaoheng Yin.

Data curation: Lingyan Liu,Yong Lai,Yongkun Deng ,Xingbiao Yang.

Formal analysis: Lingyan Liu.

Investigation: Lingyan Liu,Yong Lai,Yongkun Deng ,Xingbiao Yang, Zhaoheng Yin.

Methodology: Lingyan Liu,Yong Lai,Yongkun Deng ,Xingbiao Yang, Zhaoheng Yin.

Project administration: Yongkun Deng.

Resources:Yong Lai,Yongkun Deng ,Xingbiao Yang, Zhaoheng Yin.

Software:Xingbiao Yang, Zhaoheng Yin .

Supervision: Yong Lai,Yongkun Deng.

Validation: Lingyan Liu,Yong Lai,Yongkun Deng ,Xingbiao Yang, Zhaoheng Yin.

Visualization: Lingyan Liu,Yong Lai,Yongkun Deng ,Xingbiao Yang, Zhaoheng Yin.

Writing-original draft: Lingyan Liu.

Writing-review & editing:Lingyan Liu,Yong Lai,Yongkun Deng ,Xingbiao Yang, Zhaoheng Yin.

References

1. Grundy SM, Stone NJ, Bailey AL, Beam C, Birtcher KK, Blumenthal RS, et al. 2018 AHA/ACC/AACVPR/AAPA/ABC/ACPM/ADA/AGS/APhA/ASPC/NLA/PCNA Guideline on the Management of Blood Cholesterol: A Report of the American College of Cardiology/American Heart Association Task Force on Clinical Practice Guidelines. Circulation. 2019;139(25).

2. Figorilli F, Mannarino MR, Sahebkar A, Pirro M. Lipid profile screening and ASCVD prevention. Expert Review of Cardiovascular Therapy. 2023;21(7):463-71.

3. Goldsborough E, Osuji N, Blaha MJ. Assessment of Cardiovascular Disease Risk. Endocrinology and Metabolism Clinics of North America. 2022;51(3):483-509.

4. Vinci P, Panizon E, Tosoni LM, Cerrato C, Pellicori F, Mearelli F, et al. Statin-Associated Myopathy: Emphasis on Mechanisms and Targeted Therapy. International Journal of Molecular Sciences. 2021;22(21).

5. Mach F, Baigent C, Catapano AL, Koskinas KC, Casula M, Badimon L, et al. 2019 ESC/EAS Guidelines for the management of dyslipidaemias: lipid modification to reduce cardiovascular risk. European Heart Journal. 2020;41(1):111-88.

6. Lee J, Lee S-H. Expanding the therapeutic landscape: ezetimibe as non-statin therapy for dyslipidemia. The Korean Journal of Internal Medicine. 2023;38(6):797-809.

7. Chilbert MR, VanDuyn D, Salah S, Clark CM, Ma Q. Combination Therapy of Ezetimibe and Rosuvastatin for Dyslipidemia: Current Insights. Drug Design, Development and Therapy. 2022; Volume 16:2177-86.

8. Kim B-K, Hong S-J, Lee Y-J, Hong SJ, Yun KH, Hong B-K, et al. Long-term efficacy and safety of

moderate-intensity statin with ezetimibe combination therapy versus high-intensity statin monotherapy in patients with atherosclerotic cardiovascular disease (RACING): a randomised, open-label, non-inferiority trial. The Lancet. 2022;400(10349):380-90.
9. Choi H, Kang S-H, Jeong S-W, Yoon C-H, Youn T-J, Song WH, et al. Lipid-Lowering Efficacy of Combination Therapy With Moderate-Intensity Statin and Ezetimibe Versus High-Intensity Statin Monotherapy: A Randomized, Open-Label, Non-Inferiority Trial From Korea. Journal of Lipid and Atherosclerosis. 2023;12(3).
10. Lee B, Hong S-J, Rha S-W, Heo JH, Hur S-H, Choi HH, et al. Moderate-intensity statin plus ezetimibe vs high-intensity statin according to baseline LDL-C in the treatment of atherosclerotic cardiovascular disease: A post-hoc analysis of the RACING randomized trial. Atherosclerosis. 2023;386.
11. DU Zhenhao,YE Yanping,HE Jieqiong,LIAO Chaofeng. Effects of Rosuvastatin combined with ezetimibe on endoplasmic reticulum stress in vascular endothelial cells of patients with atherosclerosis. China Modern Drug Application. 2021, 15 (09): 151-153.
12. Feng Xinyu,Wang Shanjie,Dong Yuan,Sun Dongdong,Zhao Zhijing. Clinical efficacy of Rosuvastatin combined with ezetimibe in post-PCI patients .Heart Journal. 2019, 31 (03): 289-294.
13. Hong SJ, Jeong HS, Ahn JC, Cha D-H, Won KH, Kim W, et al. A Phase III, Multicenter, Randomized, Double-blind, Active Comparator Clinical Trial to Compare the Efficacy and Safety of Combination Therapy With Ezetimibe and Rosuvastatin Versus Rosuvastatin Monotherapy in Patients With Hypercholesterolemia: I-ROSETTE (Ildong Rosuvastatin & Ezetimibe for Hypercholesterolemia) Randomized Controlled Trial. Clinical Therapeutics. 2018;40(2):226-41.
14. Lee S-H, Lee Y-J, Heo JH, Hur S-H, Choi HH, Kim K-J, et al. Combination Moderate-Intensity Statin and Ezetimibe Therapy for Elderly Patients With Atherosclerosis. Journal of the American College of Cardiology. 2023;81(14):1339-49.
15. Lee Y-J, Cho JY, You SC, Lee Y-H, Yun KH, Cho Y-H, et al. Moderate-intensity statin with ezetimibe vs. high-intensity statin in patients with diabetes and atherosclerotic cardiovascular disease in the RACING trial. European Heart Journal. 2023;44(11):972-83.
16. Kim KJ, Kim SH, Yoon YW, Rha SW, Hong SJ, Kwak CH, et al. Effect of fixed‐ dose combinations of ezetimibe plus rosuvastatin in patients with primary hypercholesterolemia: MRS‐ ROZE (Multicenter Randomized Study of ROsuvastatin and eZEtimibe). Cardiovascular Therapeutics. 2016;34(5):371-82.
17. Kim W, Yoon YE, Shin S-H, Bae J-W, Hong B-K, Hong SJ, et al. Efficacy and Safety of Ezetimibe and Rosuvastatin Combination Therapy Versus Those of Rosuvastatin Monotherapy in Patients With Primary Hypercholesterolemia. Clinical Therapeutics. 2018;40(6):993-1013.
18. Kim BG, Lee S-J, Lee Y-J, You SC, Hong SJ, Yun KH, et al. Effect of moderate-intensity statin with ezetimibe combination vs. high-intensity statin therapy according to sex in patients with atherosclerosis. Scientific Reports. 2023;13(1).
19. Lee S-J, Cha J-J, Choi WG, Lee W-S, Jeong J-O, Choi S, et al. Moderate-Intensity Statin With Ezetimibe Combination Therapy vs High-Intensity Statin Monotherapy in Patients at Very High Risk of Atherosclerotic Cardiovascular Disease. JAMA Cardiology. 2023;8(9).
20. LI Jian-gang , JING Shao-hua , MAI Xing-sheng , LI Chao , LI Bao , CHEN Ying-cong,et al.Evaluation of Two Different Therapies of Rosuvastatin Calcium Tablets on Vulnerable Carotid Plaques in Patients with Cerebral Infarction due to Large- artery. Journal of Nongken Medicine.2020, 42 (02): 101-106.
21. MA Cao,LI Hui,XIAO Pu. Comparison of efficacy and safety of Rosuvastatin calcium tablets

combined with ezetimibe tablets in the treatment of hyperlipidemia. Journal of Pharmaceutical Forum. 2015, 36 (01): 148-149.
22. Moon JS, Park IR, Kim SS, Kim HS, Kim NH, Kim SG, et al. The Efficacy and Safety of Moderate-Intensity Rosuvastatin with Ezetimibe versus High-Intensity Rosuvastatin in High Atherosclerotic Cardiovascular Disease Risk Patients with Type 2 Diabetes Mellitus: A Randomized, Multicenter, Open, Parallel, Phase 4 Study. Diabetes & Metabolism Journal. 2023;47(6):818-25.
23. Ran D, Nie H-j, Gao Y-l, Deng S-b, Du J-l, Liu Y-j, et al. A randomized, controlled comparison of different intensive lipid-lowering therapies in Chinese patients with non-ST-elevation acute coronary syndrome (NSTE-ACS): Ezetimibe and rosuvastatin versus high-dose rosuvastatin. International Journal of Cardiology. 2017; 235:49-55.
24. Su WS. Effect of ezetimibe combined with conventional dose of Rosuvastatin on coronary atheromatous plaque. Hebei Med. 2016, 22 (08): 1340-1343.
25. Wang F. Observation on the efficacy of ezetimibe combined with rosuvastatin in the treatment of diabetes mellitus complicated with hyperlipidemia. Modern Medicine and Health. 2018, 34 (07): 1050-1052.
26. Yang Y-J, Lee S-H, Kim BS, Cho Y-K, Cho H-J, Cho KI, et al. Combination Therapy of Rosuvastatin and Ezetimibe in Patients with High Cardiovascular Risk. Clinical Therapeutics. 2017;39(1):107-17.
27. Zhang Huanji，Chen Yixi，Wu Zhiwen，Wu Guifu. Effect of ezetimibe combined with rosuvastatin on the incidence of adverse cardiovascular events in STEMI patients.New Medicine. 2018, 49 (03): 186-189.
28. Xing L, Jing L, Tian Y, Yan H, Zhang B, Sun Q, et al. Epidemiology of dyslipidemia and associated cardiovascular risk factors in northeast China: A cross-sectional study. Nutrition, Metabolism and Cardiovascular Diseases. 2020;30(12):2262-70.
29. Roth GA, Mensah GA, Johnson CO, Addolorato G, Ammirati E, Baddour LM, et al. Global Burden of Cardiovascular Diseases and Risk Factors, 1990–2019. Journal of the American College of Cardiology. 2020;76(25):2982-3021.
30. Li J-J, Zhao S-P, Zhao D, Lu G-P, Peng D-Q, Liu J, et al. 2023 Chinese guideline for lipid management. Frontiers in Pharmacology. 2023;14.
31. Almeida SO, Budoff M. Effect of statins on atherosclerotic plaque. Trends in Cardiovascular Medicine. 2019;29(8):451-5.
32. Siddiqi TJ, Ah Y-M, Jeong M, Choi HD. Comparative safety and efficacy of low- or moderate-intensity statin plus ezetimibe combination therapy and high-intensity statin monotherapy: A meta-analysis of randomized controlled studies. Plos One. 2022;17(3).
33. Hagiwara N, Kawada-Watanabe E, Koyanagi R, Arashi H, Yamaguchi J, Nakao K, et al. Low-density lipoprotein cholesterol targeting with pitavastatin + ezetimibe for patients with acute coronary syndrome and dyslipidaemia: the HIJ-PROPER study, a prospective, open-label, randomized trial. European Heart Journal. 2017;38(29):2264-76.
34. Silverman MG, Ference BA, Im K, Wiviott SD, Giugliano RP, Grundy SM, et al. Association Between Lowering LDL-C and Cardiovascular Risk Reduction Among Different Therapeutic Interventions. Jama. 2016;316(12).
35. Navarese EP, Robinson JG, Kowalewski M, Kolodziejczak M, Andreotti F, Bliden K, et al. Association Between Baseline LDL-C Level and Total and Cardiovascular Mortality After LDL-C

Lowering. Jama. 2018;319(15).
36. Sniderman AD, Williams K, Contois JH, Monroe HM, McQueen MJ, de Graaf J, et al. A Meta-Analysis of Low-Density Lipoprotein Cholesterol, Non-High-Density Lipoprotein Cholesterol, and Apolipoprotein B as Markers of Cardiovascular Risk. Circulation: Cardiovascular Quality and Outcomes. 2011;4(3):337-45.
37. Thanassoulis G, Williams K, Ye K, Brook R, Couture P, Lawler PR, et al. Relations of Change in Plasma Levels of LDL- C, Non- HDL- C and apoB With Risk Reduction From Statin Therapy: A Meta- Analysis of Randomized Trials. Journal of the American Heart Association. 2014;3(2).
38. Boekholdt SM, Arsenault BJ, Mora S, Pedersen TR, LaRosa JC, Nestel PJ, et al. Association of LDL Cholesterol, Non–HDL Cholesterol, and Apolipoprotein B Levels With Risk of Cardiovascular Events Among Patients Treated With Statins. Jama. 2012;307(12).
39. Alsayed N, Almahmeed W, Alnouri F, Al-Waili K, Sabbour H, Sulaiman K, et al. Consensus clinical recommendations for the management of plasma lipid disorders in the Middle East: 2021 update. Atherosclerosis. 2022; 343:28-50.
40. Duerden M, O'Flynn N, Qureshi N. Cardiovascular risk assessment and lipid modification: NICE guideline. British Journal of General Practice. 2015;65(636):378-80.
41. Grundy SM. An International Atherosclerosis Society Position Paper: Global recommendations for the management of dyslipidemia. Journal of Clinical Lipidology. 2013;7(6):561-5.
42. Ning G. Chinese Guideline on the Primary Prevention of Cardiovascular Diseases: Time to Start Better Cardiovascular Primary Prevention. Cardiology Discovery. 2021;1(2):65-7.
43. Budoff M. Triglycerides and Triglyceride-Rich Lipoproteins in the Causal Pathway of Cardiovascular Disease. The American Journal of Cardiology. 2016;118(1):138-45.
44. Chait A. Hypertriglyceridemia. Endocrinology and Metabolism Clinics of North America. 2022;51(3):539-55.
45. Arnett DK, Blumenthal RS, Albert MA, Buroker AB, Goldberger ZD, Hahn EJ, et al. 2019 ACC/AHA Guideline on the Primary Prevention of Cardiovascular Disease: A Report of the American College of Cardiology/American Heart Association Task Force on Clinical Practice Guidelines. Circulation. 2019;140(11).

Click here to access/download
Supporting Information
prisma checklist.docx
